
JOURNAL INFORMATION
==============================
NLM Title Abbreviation: Innov Aging
Journal ID: Innov Aging
NLM Journal ID: 101703706
Journal ID: innovateage

Innovation in Aging

EISSN: 2399-5300
Publisher: Oxford University Press

ARTICLE INFORMATION
==============================
PMCID: PMC11692925
DOI: 10.1093/geroni/igae098.4391
Article ID: igae098.4391
Article version: 1
Subjects: Participant Index, AcademicSubjects/SOC02600

Participant Index

Publication date: 2024 Dec
Electronic publication date: 2024 Dec 31
Volume: 8
Issue: Suppl 1
Issue title: Program Abstracts from the GSA 2024 Annual Scientific Meeting, “The Fortitude Factor”
First page: 1378
Last page: 1427
Copyright: © The Author(s) 2024. Published by Oxford University Press on behalf of The Gerontological Society of America.
Copyright year: 2024
License: This is an Open Access article distributed under the terms of the Creative Commons Attribution License (https://creativecommons.org/licenses/by/4.0/), which permits unrestricted reuse, distribution, and reproduction in any medium, provided the original work is properly cited.
License URL: https://creativecommons.org/licenses/by/4.0/

Page count: 1

==============================

### Participant Index

Arabic numbers refer to session numbers.
Duplicate numbers indicate the participant is listed as an author on multiple abstracts within a session.

**A**
Østbye, Truls: 2065, 3025, 7205
Abadir, Michael: 8000
Abadir, Michael P: 8000
Abadir, Peter: 1580, 2245, 4005, 8000, 8015
Abaei, Elnaz: 7355, 9045
Abbate, Lauren M: 7155
Abbing, Jens: 1120
Abbott, Katherine M: 1160, 3380
Abdallah, Lisa: 2440
Abdel Magid, Maryam S: 7425, 7905
Abdelmageed, Mofida: 8000
Abdelrahman, Nadir: 8015
Abdulrahim, Sawsan: 1155
Abe, Takumi: 7275, 8015
Abi Nassif, Alexandra: 1155
Ables, Gene: 8000
Abraham, Alison: 7285
Abrams, Leah: 4445
Abramson, Corey: 3175, 4450
Abramson, Tobi A: 7695
Abreu, Taiane: 7745
Abu-Baker, Dania: 3115
Abudahab, Sara: 7715
Abudu-Birresborn, Diana: 4175
Abul, Yasin: 7030
Abul, Zeynep: 7070
Abu-Samaha, Amir: 7065, 7425
Abyadeh, Morteza: 8000
Acevedo-Fontanez, Adrianna I: 2220
Acharya, Jhuma: 2195
Acierno, Ron: 2520
Ackerson, Leland K: 8010
Acosta, Cromwell: 7750
Adamek, Margaret E: 7515, 7540, 7610
Adams, Leah: 2450
Adams, Leslie: 7615
Adams, Swann A: 3175, 7100
Adams, Taylor: 4170
Adams-Price, Carolyn E: 4540
Adaniya, Stephanie: 3510
Adar, Sara D: 2040, 3125, 3145
Adawudu, Emefa A: 8020
Addai-Dansoh, Stephen: 7020
Addison, Odessa: 2345, 7315, 7720, 8010, 8015
Addo-Mensah, Dorothy Ewurabena: 1465, 3300
Adeagbo, Morolake: 7465, 7685
Adekoya, Adebusola: 7630, 7905
Adelnia, Fatemeh: 7010
Adeniji, Dolapo O: 7440, 7505, 7515, 7610, 7925
Adepoju, Omolola E: 3485
Adeyemi, Oluwaseun: 7465, 9020
Adil, Rawaa: 8015
Adjei, Mathias: 7240, 7405
Adjei Gyimah, Akwasi: 7240, 7405
Adjei-Poku, Michael: 8015
Adkins, Daniel E: 1485
Adnan, Maham: 7720
Adolph, Sharada Sarah: 3430
Adorno, De'Lisia: 3235
Aflatoony, Leila: 8000
Agard, Pernilla: 3550
Agboji, Aderonke: 2360
Agbomi, Lidadi: 8000
Agerholm, Janne: 7215
Aghakhani, Elham: 4220
Agmon, Maayan: 1435, 3085, 4075, 7300, 7695, 9040
Agnoli, Alicia: 8010
Agrawal, Sunil: 7750
Agrawal, Yuri: 2080
Agree, Emily M: 7420, 7920
Aguila, Emma: 1320
Aguilar, Genevieve C: 2125
Aguilar, Vanessa: 3240
Aguirre, Alyssa: 1355, 2355, 7040
Aguirre, Annabella S: 3245, 7260
Agyekum, Boadi: 7605
Agyemang, Lillian: 2265, 7155
Ahlbrand Robinson, Amy: 4240
Ahmad, Anam: 7010
Ahmad, Waqar: 8020
Ahmadi, Matthew: 1245
Ahmed, Ali: 2160, 2380, 9025
Ahmed, Ivy: 8000
Ahmed, Linda: 8015
Ahmed, Syeda A Fatima: 7530, 7905
Ahmidouch, Mohamed: 2085
Ahn, Eunhye: 8010
Ahn, Hyochol: 2240, 8010, 8015
Ahn, Jinhyun: 8015
Ahn, SangNam: 7465, 8010
Ahn, Sangwoo: 7005
Ahn, Seon Ah: 1140
Ahn, Seoyeon: 8010, 8015
Ahn, Tae-Hyuk: 8010
Aichele, Stephen R: 7455, 7720, 7925
Ailshire, Jennifer: 1015, 1320, 2040, 2110, 2460, 2515, 3125, 3245, 3540, 7040, 7500
Aishima, Miya: 7510
Ajrouch, Kristine J: 1155, 1495, 3040, 3065
Akabike, Ekenechukwu: 8010
Akbar Nejad Yazdi, Reyhaneh: 8020
Aki, Sugiura: 7330
Akiguchi, Ichiro: 8010
Akinbami, Yewande: 3340
Akiva, Pinchas: 8015
Akre, Ellesse-Roselee: 1170, 9030
Akter, Nahida: 1080, 1160
Akushevich, Igor: 3010, 3085, 7075, 7200, 7300, 7620, 7715
Al Bcherraoui, Myriam: 3040
Al Ghazo, Ali: 7320
Al Juboori, Ruaa: 4080, 7055
Al Saeedy, Dalia: 7715
Al Snih, Soham: 2020, 7205, 7300, 7440, 7700, 8015
Alam, Md Tanjim: 8000
Alam, Rifat: 7145
Alanazi, Eman: 7200
Al-Ani, Mohammad: 1445
Alaux, Alexia: 1100
AlAzzeh, Ola: 7715
Albalawi, Wafi: 9045
Albashayreh, Alaa: 8040
Albayyaa, Mohanad: 7020
Alber, Jessica: 7205
Albers, Elizabeth A: 2015, 3130
Albert, Jeffrey: 1140
Albert, Marilyn: 2525, 3070, 3555, 7015, 7200, 8015
Albert, Steven M: 1290, 2350, 2450, 2480, 3045, 3550, 7300
Alberth, Andrew G: 2225, 3075, 4270, 9000
Albertini, Marco: 3025, 7515, 7915
Albrecht, Jennifer: 1255, 4385, 8070
Alburez-Gutierrez, Diego: 4050
Alcantar, Sandy: 3460
Alcusky, Matthew: 7320
Alderson, Jacob E: 7070, 7115
Aldossary, Heba M: 1490, 7270
Aldridge, Jolaiya: 8000
Aldridge, Melissa D: 1395
Aldwin, Carolyn M: 7030
Alea, Nicole: 7250, 7635
Alejo, Prince Iverson C: 8070
Aleksic, Sandra: 1460
Aleong, Rosanne: 7040
Alessi, Cathy: 7660
Alexander, Bruce: 7665
Alexander, Gregory L: 3090, 8040
Alexander, Karah: 2295
Alexander, Laura: 8000
Alexander, Neil B: 7720
Alexanderson, Kristina: 2365
Alfonso Lopez, Irene: 3150
Alford, Heather: 4440
AlGhatrif, Majd: 8015
Alhabodal, Ashwag Saad: 8015
Alharrasi, Azza: 7420, 7455

© The Author(s) 2024. Published by Oxford University Press on behalf of the Gerontological Society of America.
This is an Open Access article distributed under the terms of the Creative Commons Attribution License (https://creativecommons.org/licenses/by/4.0/), which permits unrestricted reuse, distribution, and reproduction in any medium, provided the original work is properly cited.

Alhasan, Dana: 1045, 9000
Ali, Akilah R: 4390, 7065, 7205
Ali, Eman: 8000
Ali, Mohab: 7325
Ali, Sanna: 8015
Ali, Talha: 2045
Ali, Waad: 7605
Alimohammadbeik, Kourosh: 7630
Alkeridy, Walid A: 1150, 3335
Alladi, Renuka: 7490
Allan, Alexa C: 1250, 2055, 2330, 8000
Allard, Eric S: 4295, 8020
Allemand, Mathias: 8000
Allemann, Alexandrea L: 3425
Allen, Elizabeth M: 1345, 2315, 8020
Allen, Josie R: 7720
Allen, Julie Ober: 1095, 2330
Allen, Kelli D: 2145, 7330, 8015
Allen, Laura D: 7430
Allen, Melissa: 7040
Allen, Nancy A: 2060, 4070
Allen, Rebecca Sue: 1210, 1325
Aller, Ty B: 4435, 7065, 7270, 8020
Allgood, Sarah: 2155
Allison, Caitlin: 4215
Allison, Matthew A: 2220
Allison, Theresa A: 4480
Allman, Richard: 9025
Allore, Heather: 3035, 3520, 7060, 7465, 7680, 7925
Allsopp, Richard: 8000
Almack, Kathryn: 1015
Almeida, David: 1255, 1350, 1525, 2385, 4150, 4180, 4405, 4410, 7255, 7350, 7490, 7675, 8000
Almenar-Queralt, Angels: 7310
Al-Naggar, Iman: 3210, 4350
Alnufeay, Mashael: 7470, 7905
AlOkda, Abdelrahman: 8020
Alotaibi, Taghreed: 8010
Al-Rousan, Tala: 3065
Alshanbari, Mohammed: 7630
Alston, Daniel: 8010
Altenmueller, Eckart: 4490
Alter, Isaac L: 7140
Altona, Janissa: 4345
Alvarez, Loreli: 7425, 7615, 7700, 7930
Alvarez-McNelis, Desiree: 3375
Alves, Gilberto: 7350
Alves da Silva do Nascimento, Mariana: 3155
Am, Linda: 7110
Amadi, Grace: 8010
Amanambu, Peace Nneka: 4295
Amano, Takashi: 4190, 4265, 7630
Amaravadi, Harsha: 7080, 8010
Amato, Francesco: 2240, 7305
Ambrosio, Fabrisia: 2165
Ameer, Fathima: 7210
Amin, Iftekhar: 1485, 7125
Amin, Vikesh: 1280
Amjad, Halima: 1255, 3215, 4100
Amlerova, Jana: 8000
Amoatey, Solomon: 4000
Amor, Corina: 3160
Amponsah, Abigail: 4360
Amy, Byers: 2190
An, Jaesung: 7545
An, Yang: 1255
Anaab, Esme: 7080
Anaya-Carmona, Israel: 7200
Ances, Beau: 7655
Anctil, Reid: 7680
Andel, Ross: 2185, 8000
Andersen, Troy C: 3175
Andersen, Zorana J: 3125
Anderson, Andrea M: 1390
Anderson, Brittany: 7465, 7685
Anderson, Joel G: 2015, 2225, 3190, 7720, 8010
Anderson, Keith A: 2260, 4345, 7725
Anderson, Kennedy: 7200
Anderson, Lindsey J: 7305
Anderson, Nicole D: 1205, 7675
Anderson, Rose: 8020
Anderson, Rozalyn: 8000
Anderson, Ruth: 7535, 9030
Anderson, Stacy: 1025
Anderson, Susan J: 1175
Anderson-Steward, Kuren A: 8000
Andrade, Flávia Cristina Drumond: 3030, 7135, 8020
Andrade Rabello, Gilciney: 4465
Andreoletti, Carrie: 2440, 3515
Andres, Lanvin: 1470
Andrews, Hailey: 8000
Andrews, Heather: 7355
Andrews, Ryan: 4380
Andrilla, C. Holly: 2500
Ang, Chi Shuen: 2100, 7455, 7925
Ang, Shannon: 1095, 2065, 3405
Angel, Cybele: 4195
Angel, Jacqueline L: 2155, 3030, 8020
Angellini, Aude: 1420
Anglin, Emerald: 8070
Angus, Payton: 7430
Anjum Ashrafi, Sadia: 7145
Annunziata, Kathy: 8010
Anquillare, Elizabeth: 7225, 7500
Ansai, Juliana Hotta: 7225, 7655
Ansie, Vera: 7335
Anstey, Kaarin J: 3185, 7610, 8020
Antenor, Angelo: 7010
Anthony, Mia: 7015
Antic, Mina: 4375
Antonakakis, Antigone: 1505
Antonucci, Toni C: 1045, 1155, 1495, 3040, 3540, 9015
Anupindi, Meenakshi: 7070
Aouad, Marion: 7080
Apostolou, Hannah: 7070, 7455, 7610, 8015
Apostolova, Liana G: 8000
Applebaum, Robert A: 1315, 4510, 7295
Appleton, Allison: 8010
Apsley, Abner T: 7010, 7490
Apuan, Victoria N: 2405
Arai, Tetsuaki: 8015
Arai, Yasumichi: 7115
Aranda, Maria P: 1320, 1335, 3030, 3280
Arata, Louis: 8020
Araujo, Lia: 7745
Araujo-Menendez, Carlos EE: 7000, 7455, 7540
Aravena, Jose M: 4235
Arbeev, Konstantin: 1025, 7075, 7210, 7715
Archambault, Dennis: 1295, 4265
Archer, Hannah: 7000
Arciniega, Adrian M: 7010
Ard, Jamy D: 2505
Ardekani, Azam: 7030
Ardon Lobos, Marcos: 8015
Aree-Ue, Suparb: 7205, 7440
Arellanes, Isabella C: 7630
Arensberg, MaryBeth: 8015
Arias, Dalmina: 8015
Arigoni, Danielle: 2460
Arivudainambi, Aishwarrya P: 4530
Arling, Greg: 7510, 7915
Armando, Antonio: 8010
Armendariz, Marina: 1495
Arms, Tamatha: 8015
Armstrong, Grace: 1490
Armstrong, Naomi: 8015
Armstrong, Nicole: 8015
Arnette, Shannon M: 1145, 3510, 8010
Arnold, Michelle: 7140
Arnold, Moriah: 7310
Aronow, Wilbert: 9025
Arora, Neeraj: 3340
Arora, Shambhavi: 8020
Arp, Patrick C: 7000
Arpawong, Thalida Em: 1455, 3220, 4375
Arreguin, Berty: 1245
Arteaga, Ignacia: 3175, 4450
Arterburn, David: 1265, 9020
Arthanat, Sajay: 3185
Arthur, Elizabeth K: 2445
Aruscavage, Nancy: 3500, 7425, 7905
Arya, Shipra: 3435
Aryoyudanta, Bayu: 8015
Asante-Danso, Anthony J: 8000
Aschenbrenner, Andrew J: 4515
Ash, Kelly: 2450
Ashcraft, Alyce S: 7080
Ashcraft, LauraEllen: 9025
Ashe, Maureen C: 8020
Ashebir, Sophia: 1095, 7065, 7090
Ashford, Nathanial K: 8000
Ashida, Sato: 2185, 3345, 3485, 4220, 7465, 7685
Ashiqueali, Sarah A: 8000
Ashirifi, Gifty D: 7515, 7610
Ashok Kumaar, Prasanna Vadhana: 8000
Ashrafi, Jaya: 7015
Asiedu, Charlotte: 7700
Askim, Torunn: 7060
Aslan, Ilker: 7160
Asraf, Kfir: 1435

Assadi Khalil, Samar: 7300
Assi, Lama: 3275
Assi, Sahar: 2525, 3275
Assumpção, Daniela de: 8015
Asthana, Sanjay: 3225
Aswani, Truphosa: 2015, 3025
Atak, Selen: 7455
Atayee, Rabia: 1435
Atchison, Chandler: 4160
Atherton, Olivia E: 4480
Atif Mohamed Yousif, Mohamed: 8015
Atkin, Andrew: 1245
Atoyebi, Oladele: 7155
Attuquayefio, Tuki: 3185
Atul Patil, Vaishnavi: 7430
Au, Daisy: 3200
Aubry, JP: 2420
Audley, John: 3340
Auriemma, Catherine L: 8015
Austad, Steven N: 4210
Autar, Kaveena: 7425, 7630
Au-Yeung, Wan-Tai M: 3285, 8020
Avendaño Villaseñor, Abigail: 7465
Avent, Elizabeth S: 2001
Avesani, Carla Maria: 3330
Avidon, Irina: 7320
Avila, Jaqueline C: 1405, 2205, 8010, 8015
Avis, Fan: 7490
Awata, Shuichi: 7350, 7490, 7640, 8015
Axelrod, Jenna: 7495, 7930
Axelson, Eric: 7735
Ayala, Alba: 4045
Ayalon, Liat: 1190, 2460, 7420, 8010
Ayers, Lindsay R: 7640
Azar, Ariel: 1110, 3410
Azhar, Gohar: 7010, 7210, 7465, 8000, 8015
Azhir, Alaleh: 1575
Azuero, Andres: 7285, 7700

**B**

Ba, Fang: 7465, 7925
Bárbara, Rita Cássia: 8010, 8015
Bédard, Marie-Ève: 3320
Bünning, Farina: 8010
Baars, Jan: 1180
Babenko, Natalia: 7070
Babitzke, Jennifer: 2500
Baby, Eliza: 7660
Bachner, Yaacov: 3530
Bacon, Cindy: 7275
Bacsu, Juanita R: 3175, 8010
Badal, varsha E: 4180
Badawi, Dina T: 8015
Bae, Myeongjin: 7655
Baek, Daniel: 4355
Baek, Jihye: 1295, 2260, 4140, 4265, 7290, 7630
Baek, Jonggyu: 7320
Baek, Sehyun: 8010
Bagley, Olivia: 1025
Bahti, Melanie: 8015
Bai, Chen: 1445
Bai, Chyi-Huey: 7735
Bai, Xue: 8010, 8020, 8050, 8090, 8095
Bai, Yaru: 4055
Baier, Rosa R: 3240
Baik, Dawon: 7360, 7660
Baik, Sol: 1045
Bailey, Dionne: 3130, 4445, 7685
Bailey, Donald: 9030
Bailey, Olivia: 8020
Bailey-Taylor, Melissa: 8010
Baillargeon, Emma M: 2510, 4215
Baim-Lance, Abigail: 8010, 8010
Bain, Catherine: 7420
Baird, Lola: 2385
Baird, Sara: 7080
Bak, Katherine: 7225
Bakare, Jemimah: 7260
Baker, Christina: 8000
Baker, Hamilton: 2355
Baker, Jillian: 1365
Baker, Kristin: 7740
Baker, Natalie R: 1200
Baker, Shelby: 3450
Baker, Suzanne: 9000
Baker, Tamara A: 2110, 4000, 7700
Baker, Zachary G: 3130, 9000
Bakk, Louanne: 2265, 7155
Balaam, Kori: 2330
Balachandran, Arun: 4550
Balaji, Anisha: 8035
Balasundaram, Rohit Baal: 9050
Balbim, Guilherme M: 7455, 7720
Baldinger, Samantha: 7695
Baldizar, Deborah: 7415
Baldyga, Kathryn: 7450, 8000
Bales, Diane: 3575
Ball, Karlene: 4490, 7455
Ball, Sarah: 1490
Ballew, Shoshana: 3570
Ballreich, Jeromie: 7470, 7905
Balmuth, Alexa: 7065, 7090
Balogun, Saliu: 4000
Balogun, Zainab: 1070
Balsa, Jonah: 7010
Balser, sarah: 1060
Baltasar-Fernandez, Ivan: 8020
Balubaid, Afnan: 4095
Bamdas, Jo Ann M: 3190
Bamonti, Patricia: 7720
Banarjee, Chitra: 7205, 7640, 8015, 8020
Banaszak-Holl, Jane: 1550
Bancks, Michael P: 3180
Banco, Julia: 8020
Banday, Muneeb Ul Lateef: 3315
Bandeen-Roche, Karen J: 1130, 1205, 2230, 7620
Bandholm, Thomas Q: 4075
Bandi, Sriya: 7215
Band-Winterstein, Tova: 3015, 7265, 8090
Bandyopadhyay, Attrayee: 1485
Bangen, Katherine J: 7005, 8010, 8015
Bangerter, Lauren R: 1270, 7640, 8020
Baniassadi, Amir: 2040
Banisalman, Islam: 1465
Bankole, Ayomide Okanlawon: 3300, 7440, 7925
Banks, Jack: 2075
Banks, Russell: 7425, 7635, 7930
Banner-Lukaris, Davina: 2360
Banno, Ryoichi: 7315
Bannon, Sarah M: 3250
Banskota, Nirad: 7210, 7305
Bao, Dapeng: 4535
Bar, Rachel J: 7040
Barada, Fatmeh: 1155
Baral, Stefan: 8015
Baralt, José: 3265
Baranes, Guy: 1245, 2240
Barbakoff, Daniel: 1005
Barbee, Harry: 1095, 1170, 9030
Barber, Claire: 8010
Barber, Emma: 1130
Barbera, Gina: 2130, 8015
Barclay, Ruth: 7300
Barclay, Terry: 7455
Bard, Skylar: 7360
Bardales, Rhonda L: 3275
Bardo, Anthony R: 1480
Barha, Cindy: 3335, 7720
Barinas-Mitchell, Emma: 7625
Barker, Sandra: 8020
Barneclo, Marcella A: 8015
Barnes, Deborah E: 3010, 3175, 4230, 7005
Barnes, Lisa: 2110, 2215
Barnes, Mary E: 7225
Barnes, Stacy: 7480
Barnett, Melissa A: 8020
Barnett, Michael: 7750
Barnhart, Emma M: 7505
Baron, Aliza R: 7250
Barooah, Adrita: 3065
Barr, Ashley: 7225
Barragan, Cassandra L: 1530
Barragan, Noel: 7015, 7200
Barral Rodriguez, Sandra M: 1025
Barrett, Anne: 1010, 7160
Barrett, Tyler D: 3010
Barron-Vaya, Yolanda: 4360
Barrus, Hannah: 2505
Barry, Lisa C: 3420, 7740
Bartels, Stephen J: 2315, 3250
Bartke, Andrzej: 8000
Bartlett, Paige: 7415, 7900
Bartlett, Robin M: 3450
Bartlett, Sean: 3485
Bartley, Jenna M: 1275
Barton, Kelli N: 3565
Barton-Ort, Kelly: 7315
Basaran Sahin, Duygu: 7050
Basaria, Shezhad: 7285
Bashian, Hannah: 2385, 8020
Basic, Ajla: 7505, 7935
Basrai, Zavera: 2500
Bass, David M: 1030, 1265
Bass, Judy: 7615
Bassola, Barbara: 7245
Basu, Disa: 8000
Basu, Kaustuv: 8000
Basu, Rashmita: 7200, 7425, 7905
Batamack, Yvann: 8000
Batchelor, Melissa: 8015, 8035
Bate, Angela: 4255
Bateman, Randall: 8015
Bates, David: 7425, 7930
Batista-Malat, Eleanor: 2150, 2375
Batsis, John A: 2505, 3580, 3585, 7275, 7535
Batura, Josey M: 2065, 7065, 8020

Baucco, Christina M: 8020
Bauer, Scott R: 4350
Bauman, Alexandra: 7350
Baumann, Sara E: 7300
Baumgart, Matthew: 3000, 4235, 7005, 7200
Bayer, Thomas: 7200
Bayliss, Elizabeth: 2085
Bayly, Melanie: 7135
Bayne, Alycia: 1425
Beach, Brian: 2365
Beach, Douglas: 1265
Beagley, Lindsey: 4320
Beam, Christopher R: 1215, 3220, 7725
Beamer, Brock: 7440
Bean, Jonathan F: 3335, 4215
Beans, Jeffrey: 7315
Beare, Richard: 4100
Beatriz Simões Pereira, Ana: 7655
Beattie, Zachary: 3285, 7020, 7735, 8020
Beatty, Jennifer F: 8020, 8080
Beauchamp, Marla: 2170, 7655, 7720
Beaudreau, Sherry A: 7235
Beauregard, Lisa: 7295
Beavers, Daniel: 2505
Beavers, Kristen: 1410, 2505
Becares, Laia: 1110
Bechek, Bonnie: 3175
Beck, Emorie D: 4515, 7455
Becker, Clemens: 2170
Becker, David J: 3255
Becker, Heather: 7145, 7435
Becker, Jacqueline H: 7630
Becker, Marigrace: 3510
Becker, Todd D: 1165, 4080, 7020, 8010
Beckner, Tara: 1390
Bedard, Michel: 3185
Beeber, Anna: 7535, 8035
Beech, Bettina M: 3475, 7700
Beehler, Melanie: 7650
Beerman, Isabel: 3325
Behrend, Abigail: 4295
Behrens, LIza L: 1080, 4290, 4320
Behrman, Jere R: 1280, 7455, 7925
Bei, Eva: 7515, 7915
Beiglary, Shevvaa: 7200, 9015
Beiser, Alexa: 7500, 7930
Bejjani, Rachele: 7640
Bekelman, David: 2180
Bektas, Arsun: 7210
Belalcazar, Kimberly: 7045
Belanger, Chris: 1010
Belanger, Emmanuelle: 3535, 7295
Belazis, Laura: 1190
Beld, Matthew: 8025
Belin de Chantemele, Eric: 7285
Bell, Bethani: 2555
Bell, Janice: 2200, 7145, 8010
Bell, Mallory: 7535
Bell, Tyler: 8020
Bellelli, Giuseppe: 4315, 7010
Belleville, Sylvie: 4490
Belmonte, Juan Carlos Izpisua: 2310
Belsky, Dan: 4550
Beltran, Susanny J: 7055
Belvederi Murri, Martino: 3035
Belza, Basia: 2115, 2450, 3140, 4235, 7415, 7465, 7610, 7900, 8015
Bendapudi, Arati: 8015
Bender, Alexis: 8020
Bender, Alexis A: 8020
Bender, Andrew R: 8055
Bender, Anna E: 1490
Bene, Michael R: 7305, 8000
Benfer, Jessica: 1070
Benge, Jared: 7040
Benjamin, Cynthia: 7005
Benjasirisan, Chitchanok: 7440, 7500, 8000
Bennett, Antonia: 4080
Bennett, David: 1235, 4005, 8000
Bennett, Henrietta Armah: 7040
Bennett, Katherine A: 4470
Bennett, Stephanie: 2295
Bensenor, Isabela G: 1320
Bensimon, Moshe: 2001
Benson, Clark: 3425, 7200
Benson, Jacquelyn J: 7490, 8000, 8010
Benton, Donna: 2375, 7665, 8010
Benton, Leilani: 8020
Benyassine, Karim: 9000
Benzel, Heather: 1395
Berg, Kristen: 7690, 8000
Berg, Russell: 1400
Bergeman, Cindy S: 7255
Bergh, Marissa: 8010, 8035, 8070
Berghoff, Ashley: 4095
Bergstrom, Jaclyn: 1435, 2235, 4170, 7010, 7425, 8000
Berg-Weger, Marla: 4080, 8020
Berian, Julia R: 3335, 4530
Beridze, Giorgi: 3330, 7060
Berish, Diane: 3230, 3380, 7040, 7410, 7455, 8015, 9015
Berkman, Cathy: 2225
Berkowitz, Jennifer: 8010
Berkowsky, Ronald W: 7640
Berlinger, Nancy: 2245, 8065
Berman, Brian: 3565
Berman, Jackie: 7695
Bernacki, Gwen M: 7205
Bernal, Darren: 8020
Bernal-Alonso, Amaya: 4045
Bernard, Dominique D: 7225
Bernard, Marie A: 3400
Berner, Jon: 8000
Berner, Karl F: 8000
Bern-Klug, Mercedes: 8010
Bernstein, Isaac A: 2080
Bernstein Sideman, Alissa: 3065, 7325
Berridge, Clara: 1345, 2125, 2355, 2565, 3065, 3315
Berry, Donna L: 8015
Berry, Sarah: 1275
Berry-Stoelzle, Maresi: 2385, 7665
Berta, Whitney: 3170
Bertram, Lars: 7030, 7210
Bertrand, William: 8020
Besnier, Florent: 4490
Bessen, Sarah: 2035
Besser, Lilah M: 4430, 8020, 8025
Best, John: 7720
Best, Latrica E: 4000
Best, Ryan: 8000
Best, Tristin: 4300
Bethell, Jennifer: 8015
Bethune, Tyrone: 3430
Betley, Wendy: 7480
Bettencourt, Amie: 7710
Betz, Elizabeth: 7275
Betz, Joshua R: 1440
Beydoun, May A: 1250, 2330, 8000, 9020
Beydoun, Safa: 4005
Bhagianadh, Divya: 3115
Bhalla, Nalini S: 8015
Bhandari, Mira: 4005
Bhansali, Rebekah A: 8000
Bhasuran, Balu: 1355
Bhat, Aarti C: 7490
Bhat, Ajay: 4005
Bhatta, Tirth: 1140, 7660
Bhattacharjee, Anindita: 4170
Bhattacharyya, Kallol Kumar: 7355
Bhattarai, Pratik: 3055
Bhattarai, Preeti: 3055
Bhatti, Sumaya: 4465
Bherer, Louis: 4490
Bhowmick, Pallabi: 3530, 8020, 9025
Bhurtel, Shikha: 2200, 8010
Bibbo, Jessica: 7405, 7470, 8020
Bibriescas, Natashia: 7425, 7615, 7930
Bidwell, Julie: 8010
Bieber, Heather: 7320
Bielak, Allison A M: 4185, 7225
Bielsten, Therése: 4010, 4335, 8015
Bierman, Arlene S: 1205, 4160, 8030
Biernot, Kelsey M: 8020
Biesmans, Guido Anna Maria Carolus: 7295
Big Eagle, Tosha R: 7530
Biggerstaff, Matthew: 1065
Bilimoria, Karl: 1130
Binder, Ellen F: 1275
Bing-Canar, Hanaan: 8020
Binns, Malcolm: 7675
Birati, Yosefa: 8020
Birchander, Ellen: 3495
Birditt, Kira: 4085, 4525, 7660, 8020
Birkeland, Robyn W: 3130, 8020
Biruk, Cal: 7750
Bise, Christopher: 7330
Bishop, Alex: 1385, 7115, 7150
Bishop, Christine E: 1230, 1315
Bishop, Lauren: 4475
Bishop, Nicholas J: 3035, 3540, 8010
Bishop, Sonia K: 1480, 7200
Bishop, Teddy: 1080, 7250
Bitto, Alessandro: 7210
Bivas-Benita, Maytal: 8015
Björk, Jonas: 1285
Bjarnadottir, Ragnhildur I: 8015
Bjelka, Ava T: 1010
Black, Kathy: 7415
Black, Sheila: 8000, 8020
Blacker, Deborah: 1545
Blackshear, Chad: 1000

Blackwell, GinaMari: 8010
Blackwood, Jennifer: 7420, 7655, 7920
Blain, Hubert: 2170
Blake, Jason A: 7285
Blake, Jason: 8020
Blancato, Bob: 2390
Blancato, Robert: 8015
Blanchette, Caroll-Ann: 4490
Blank, Sabrina: 1540
Blanken, Anna E: 1325
Blaskewicz Boron, Julie: 7485, 7675, 8000, 8020
Blaum, Caroline S: 2075, 7465
Bleijlevens, Michel HC: 4255, 7295, 7685, 8015
Blind, Melissa: 2335, 2395
Blinka, Marcela D: 4300
Block, Laura: 2550, 3425, 7200, 8010
Block, Robert: 7420, 7920
Blodgett, Joanna M: 1245
Blonquist, Traci: 8000
Bloom, David: 7275
Bloom, Rachel F: 1165, 2305
Bloomberg, Mikaela: 3095
Blount, Amber: 7735, 7750, 8000, 8015
Blow, Frederic: 7490
Blozis Villareal, Shelley A: 7700
Bluck, Susan: 7020
Blue, Benjamin: 7010
Bluestein, Daniel: 4510
Blumen, Helena M: 7750
Boa Sorte Silva, Nárlon Cássio: 7455, 7720
Boafo, Jonelle: 1395
Boakye, Alfred: 8010, 8015
Boardman, Charles: 1125
Boateng, Isaac Asirifi: 2490, 7250, 7480
Boateng, Josephine: 7135, 7695, 7700
Bobitt, Julie: 3115, 7545
Bock, Jarrod: 7200
Bockting, Walter: 3075
Boddie-Forbes, Ra'Sheda: 4040
Bodner, Ehud: 1565, 2030
Boerner, Kathrin: 7040, 7065, 7660
Bogdan, Ryan: 8010
Bogdewiecz, Taryn: 8010
Boger, Jennifer: 2410, 2530
Bohlke, Kayla: 8000
Bohnen, Nicholaas: 2510, 8000
Bolkan, Cory: 1190, 2180, 2325, 7350, 7530
Bollin, Salli: 8020
Bolling, S. Alison: 8000
Bollinger, Rebecca: 7655, 8015
Boltz, Marie: 1080, 2140, 3130, 3230, 7040, 7410, 7420, 8015
Bolz, Fabio: 7230
Bona, Matthew: 9040
Bonds Johnson, Kalisha: 4165
Bong, Ji Yae: 9010
Bongiorni Meadows, Jill: 8000
Bonin Pinto, Camila: 8015
Boockvar, Kenneth: 8020, 8055
Booker, Emma: 2370
Booker, Staja: 8010
Boon, Jeffrey T: 1265, 7075
Boone, Anna: 7275
Boot, Walter: 1040, 2355, 3060, 3415, 4130, 4420, 7360, 8000, 8020
Booth, Richard: 4270
Borate, Samruddhi Nandkumar: 7200
Bordes, Piper J: 2300, 2330, 7045
Borgards, Kaila: 4525
Borgogna, Joanna-Lynn C: 7210
Borisoff, Jaimie: 7155
Boron, Julie B: 4205
Borrelli, Belinda: 8015
Borrero, Lisa: 8010
Borrero De Jesus, Jane: 7410, 7900
Borson, Soo: 1430, 7325
Bortey, Dennis: 4175
Borth, Laura: 8015
Boscardin, John: 7005
Bose Brill, Seuli: 2415
Boström, Anne-Marie: 1050, 7215
Bosworth, Brooke: 8010
Botana Gronek, Melissa P: 8015
Botoseneanu, Anda: 3035, 7455, 8010
Botwe, Paul: 8000
Bouaouina, Zineb: 7645
Bouchard, Lauren: 3195
Boucher, Nathan A: 2090, 2485, 2500
Boucher, Sarah: 7135
Boudreaux, Madison T: 3275
Boudreaux, Michel: 8010
Boulay, Marc: 4085
Bouldin, Erin: 2545, 3100, 3320, 3430, 4390, 4520, 7335, 7630, 7730, 7740
Boulougoura, Afroditi: 8000
Bouranis, Nicole: 4130, 4340
Bourassa, Kyle J: 8060
Boustani, Malaz: 4230
Boutilier, Justin J: 7510
Bouton, Lauren J: 1110
Bove, Allyn M: 3400, 7330
Bowblis, John R: 3255, 4305, 7295
Bowditch, Corinne: 7465
Bowen, Holly: 8020
Bowen, Lauren M: 3310
Bower, Emily S: 1020, 4120, 4180, 7630
Bowers, Barbara J: 4465
Bowes, Aaron: 8000
Bowland, Sharon E: 8010
Bowles, Kathryn: 3105
Bowles, Kathryn H: 4550
Bowman, Caitlin R: 7000
Bowman, Grace I: 4430, 7420
Boyd, Cynthia: 2085, 4055
Boye, Alyssa: 7455
Boyle, Julie: 2370
Boynton, Harrison: 7750
Brach, Jennifer S: 4215
Bracy, Danny: 7440, 7925
Bradley, Dana B: 3120, 7430
Bradley, Lisa D: 8010
Bradt, Joke: 9010
Brady, Brooke: 7610, 8020
Brady, Loretta: 2130
Brady, Samantha: 7050, 7065
Brage, Shelby: 8010
Bragg, Alexa: 7700
Bramajo, Octavio N: 1170, 4455, 7230, 7300
Brandas, Benedetta: 1235
Brandis, Leah M: 8010, 8020
Brandt, Anita: 4550, 7205
Brandt, Martina: 2025
Brankaert, Rens: 3425
Brannock, M. Daniel: 1370, 2150, 3270
Brashear, Jessica S: 7080
Brasher, Melanie S: 2130
Brasky, Theodore M: 2445
Brassolotto, Julia: 4445
Bratches, Reed WR: 4045
Bratsberg, Bernt: 2425
Bratzke, Lisa: 8065
Brauer, Simon G: 3040
Brault, Matthew: 8015
Braun, Kathryn L: 4015, 7055, 8000, 8020
Braunstein, Joel B: 7425, 7930, 8070
Bravo, Adrian: 7065
Brazg, Tracy: 4470
Breakwell, Susan: 7480
Breckling, Meghan: 7325
Breder, Kelseanne P: 3075
Brekke, Bailee: 1315, 3275, 4510
Brenes, Gretchen A: 7490
Brenick, Alaina: 2175
Brenman, Noah: 8010
Brennan, Daniel KM: 2010, 3150, 4420
Brennan-Ing, Mark: 3490, 4225
Brenowitz, Willa D: 8015
Brett, Jeanne: 7490
Breunig, Ian: 1015, 7420
Brewer, Judson: 2315, 3250
Brewer, Julie: 2315
Brewer, Robin N: 3315, 7030
Brewster, Glenna S: 4165, 7270
Breyfogle, Rachel: 3215
Briceno, Alexandra: 7540, 7735
Brickman, Adam: 7350
Brickner, Carlin: 4550
Briggs, Emily: 7690
Brightman, James D: 3055, 7025, 7735
Brinkley, Tina E: 7710
Brinkley-Rubinstein, Lauren: 3420
Brinton, Daniel: 1075
Bristol, Alycia: 2060
Britt, Katherine C: 2055, 2190, 8020
Britton, Karysa: 4515
Brock, Kaye: 3085
Brocklebank, Laura Ann: 3050, 4500
Brodrick, Marisa-Francesca: 3110
Brody, Abraham A: 2415, 3010, 4360, 7645
Brody, Abraham: 8045
Broen, Tiana M: 7695, 7720, 8020
Broese Van Groenou, Marjolein I: 1120
Brook, Juliette: 8010
Brooks, Holly: 7025
Brooks, Jada L: 8015, 8020
Brooks-Smith-Lowe, Sharifa: 7325

Brothers, Allyson: 3515, 3575, 4320
Brotman, Rebecca: 7060, 7210
Broughton, Susan: 3060
Broussard, Kimetha L: 2550
Brown, Cait: 7080
Brown, Candace: 7425, 7930
Brown, Casey L: 3355
Brown, Casey: 8020
Brown, Cynthia J: 4075
Brown, Diane K: 7475, 7900
Brown, Ellen L: 4060
Brown, J. Scott: 1505, 1535
Brown, Karl J: 3435, 7205
Brown, Kevin: 2505
Brown, Lana M: 7480
Brown, Lauren: 8020
Brown, Leacey E: 7725
Brown, Lynn: 7275
Brown, Maria T: 7100
Brown, McKenzie: 7450
Brown, Monique J: 2215, 4105
Brown, Nancy L: 7250
Brown, Rebecca T: 7290
Brown, Rebecca: 9025
Brown, Ryan L: 1570
Brown, Sarah E: 7060
Brown, Susan H: 1175, 3085, 7205, 7610
Brown, Susan L: 3065
Brown, Sylvia: 3150
Brown, Teneil: 4460
Brown, Todd T: 7285, 8010
Brown-Huges, Travonia: 8000
Browning, Wesley R: 1510, 2215, 7040
Broyhill, Tatum: 3115
Brubaker, Malaak: 8020
Bruce, Marino A: 3475, 7700
Bruckenthal, Patricia A: 3265
Brunet, Anne: 2310
Brunker, Cherie: 8020
Brunkert, Thekla: 4255
Brunt, Christopher: 3255
Bryan, Angela D: 3115
Bryan, Robert N: 3180
Bryant, Ashley L: 8035
Bryant, Brittni: 3150
Bryant, Elizabeth A: 1165
Bryant, Gillian: 7250
Bu, Feifei: 4440
Buchanan, Jeff: 7510, 7605
Buchanich, Jeanine M: 3375
Buchheit, Katie: 7400
Buchman, Aron: 1235
Buck, Harleah: 2370, 8010
Buckley, Kiera: 8020
Buckley, Matthew: 2310
Buckley, Nina: 8010
Buckwalter, Joseph A: 7205
Bucy, Taylor I: 4445
Buddendorff, Lauren: 7210
Budhathoki, Chakra: 8000
Budnick, Andrea: 7110, 8010
Budreau, Josyaah: 2395
Buehrer, Tabea: 1220
Buell, Susan: 1015
Buenning, Mareike: 2025
Bueno, Michael: 2340
Buffel, Tine: 1180, 3200
Bulanda, Jennifer Roebuck: 1505
Buljac-Samardzić, Martina: 7445
Bullard, Misty: 8020
Bullock, Karen: 2415, 3275, 4360
Bumbalova, Katerina: 1165
Burack, Orah R: 8010
Burant, Christopher J: 7265, 7660
Burch, Brittany: 4205, 7720
Burden, Sorrel: 2530
Burdick, David C: 2275, 3515, 7425, 7905
Burgdorf, Julia G: 3105, 4360, 4460
Burgess, Elisabeth O: 8020
Burgos Zuniga, Claudia C: 7250, 7480
Burgos-Melendez, Yesenia: 4475
Burkart, Britney J: 7240
Burke, Christopher: 7625
Burke, James: 2090
Burke, Robert E: 9025
Burke, Shanna L: 7540, 7735
Burkhardt, Summer: 7300
Burk-Leaver, Erin Y: 3080, 7285
Burman, Bonnie: 8020
Burnaevskiy, Nikolay: 7010
Burnes, David: 2150, 4365, 7405
Burnett, Jason: 2520, 4355
Burnette, Denise: 1140
Burningham, Zachary: 7135
Burns, Edith: 4100, 8010, 8015
Burns, Jeffrey: 8000
Burns, Madison: 2180
Burns, Reilly: 7000
Burns, Shane D: 4000, 4455
Buron, Bill: 2550
Burr, Jeffrey A: 1360, 1405, 2205, 3545, 7270, 7350, 8015
Burrage, Rachel: 7055
Burrows, Brett: 8015
Burton, Jordan B: 3045
Burton, Melanie: 8015
Burton, Shandra D: 3500
Bush, Noah: 7720
Buta, Brian: 1205
Butler, Rachel A: 7110
Buto, Peter T: 1545
Butt, Maayra I: 8015
Butters, Meryl: 7640
Buxbaum, Channa M: 7040
Buxton, Michael R: 1210, 7070, 7455, 7610
Buxton, Orfeu M: 1250, 2290, 7210, 8000
Buys, David R: 3575, 4040
Buysse, Daniel J: 2290
Byambaa, Altan-Ochir: 7205
Bybee, Sara G: 2225, 2480, 3400, 3500, 7425, 7905
Byerley, Joella: 8015
Byerly, Laura: 8010
Byers, Amy: 3010
Bylund, Carma: 7020
Bynum, Julie: 3000, 8015
Byrd, DeAnnah R: 2190
Byrd, Goldie S: 1315
Byrd-Craven, Jennifer: 8020
Byun, Eeeseung: 7735, 8000
Byun, Jun Y: 7285

C

Caban-Holt, Allison: 1315
Cabral Junior, João: 7350
Cabrera Lozoya, Daniel: 1485, 7145
Cabrero Castro, Jose Eduardo: 4550, 7230
Cacchione, Pamela Z: 3555
Caceres, Maria: 8020
Cachioni, Meire: 8015
Caefer, Jacqueline: 2450
Caggiu, Giulia: 7275
Cagle, John: 1070, 4195, 7055, 7110
Cai, Catherine: 8000
Cai, Gege: 1230
Cai, Huaibin: 8000
Cai, Shubing: 8020, 8055
Cai, Wenjie: 4380, 7275
Cai, Xinxin: 8020
Cai, Xiuting: 3270
Cai, Yurun: 7720
Cain, Stephen M: 7205
Cala-Garcia, Juan: 4170
Calamia, Matthew: 7045
Calasanti, Toni: 8080
Calderón-Larrañaga, Amaia: 3035, 3330, 4045, 7010, 7060, 7215
Calderon, Russell J: 1535, 2465, 3335
Caldwell, Clover: 8020
Caldwell, Jessica ZK: 8055
Calfee, Kristin: 7130, 7735
Calhoun, Katie: 1295, 2070
Calkins, Margaret: 4325, 8015
Callaway-Lane, Carol: 2280
Calligaro, Hugo: 7015
Callow, Daniel: 3070
Calo, William: 1080
Calver, Jen: 3010, 8010
Camacho, David: 3280
Camacho, Karen: 7660, 7735, 8015
Cambara, Celine: 7540, 7735
Camell, Christina: 3160
Cameron, Erinn C: 2040
Cameron, Kathleen A: 1005
Cammer, Allison: 4195, 4440
Camp, Alyssa: 1010
Camp, Cameron: 1070
Camp, Lauren A: 8015
Campbell, Kenna: 3255, 7545
Campbell, Leah: 2265
Campbell, Matthew D: 7075
Campbell, Sarah: 2340
Campetti, Randi: 1145, 4355
Campione, Joanne R: 4450
Campos, Annalis: 2505
Campos, Baltazar: 8010
Campos, Jennifer L: 1070, 2410, 4300, 7225, 7635
Campos, Matthew: 1195, 8010
Can, Reyyan: 4020
Candrian, Carey: 3490
Canham, Sarah L: 1295, 2395, 2560, 4445, 7445
Cannell, Brad: 4355
Canning, Shelley E: 7465
Cannon, Melissa: 3560
Cannon, Rachel M: 1030
Cantave Burton, Cassandra: 3015
Cantrell, Sarah: 4530
Cantu, Phillip: 3030, 8020
Cao, Ji: 7610
Cao, Jiawei: 1215, 2030
Cao, Katy (Qiuchang): 1280
Cao, Meng: 7440
Cao, Rui: 7225
Cao, Xuemei: 3025

Cao, Yumeng: 2160, 7735, 8010
Cao, Yunqi: 3270
Cappelletti, Monica: 7660
Cappi, Carolina: 7350
Cappon, Davide: 7355
Caprio, Thomas: 8010, 8035
Caraballo, Christina: 3090
Carbajal-Madrid, Pedro: 7695
Carballea, Denise: 8010
Carballo-Casla, Adrián: 3330
Carbuccia Abbott, Maryan: 8010
Carder, Paula: 2185, 3535, 4510, 8010, 8020, 9045
Cardoso-Arias, Rafael: 2125
Carey, James: 2245
Carlile, Robert M: 8070
Carling, Stacy: 7440, 7490
Carlozzi, Noelle: 7065
Carlson, Chalise: 3110, 7135, 7235
Carlson, Michelle C: 1240, 2045, 3375
Carlston, Kelsey: 4035
Carmiol-Rodriguez, Priscilla: 2115, 7415, 7735, 7900
Carnahan, Jennifer L: 7465
Carnavali, Fernando: 7630
Carneiro Alves de Oliveira, Bruno: 7350
Carney, Maria: 8015
Carnie, Martha B: 3340
Carolan, Kelsi: 1105
Carpenter, Brian: 1325, 4000, 7055, 8000, 8010
Carpenter, Joan: 1080, 1160, 8010
Carpenter, Joan G: 8015
Carpenter, Matthew: 7655
Carpenter, Rebekah: 4140
Carpentieri, JD: 2365
Carpio, Rubi: 7000
Carr, Dawn C: 1280, 3380, 3540, 3555
Carr, Deborah S: 3410, 4030, 4330, 4505
Carr, J Jeffrey: 2220
Carr, Lucas: 7735
Carranza, Erick: 1210, 7070
Carrera, Robert: 7750
Carrero, Juan-Jesús: 3330, 7060
Carrico, Catherine: 7440, 7490
Carrillo, Audrey: 8000
Carrington-Lawrence, Stacy: 1340
Carrion, Carmen I: 1495
Carrion, Iraida V: 2445
Carroll, Isabella: 1345
Carroll, Judith: 2005
Carson, Erin: 2500
Carson, Paul: 7090
Carstarphsen, Kathy J: 3275
Carstensen, Laura: 1575, 3355, 4420, 7255, 8000, 8020
Cartledge, Anne: 2395
Carwile, Hope: 3240
Casanova, Ramon: 7015
Casati, Fabio: 1140
Case, Donna J: 7155
Case Fankhouser, Rebekah: 7035
Casey, Joan A: 4035, 4375
Caskie, Grace I L: 7055, 7475, 7605
Cassidy, Jessica: 4105, 7155, 7340
Cassidy, Terri: 7160
Cassidy-Eagle, Erin L: 1135
Castañeda-Avila, Maira A: 7060
Castaneda, Moraima: 8025
Castro, Hugo: 4235
Catlow, Jodi: 7495
Catt, Wade: 8000
Cauble, Christina: 2535
Cauble, Heaven: 8000, 8020
Cauley, Jane A: 1365, 7060
Cavalli, Stefano: 1220
Cavanagh, Lucia: 2020
Cavanaugh, Robert: 4225, 8015
Cavanaugh, Sheamus: 2335
Cawthon, Peggy M: 1090, 1240, 1435, 2080, 2220, 4005, 4260, 4310, 7060, 7640, 8000, 8020
Ceide, Mirnova: 8000
Cejtin Rosen, Francesca W: 4370
Celestin, Michael David: 8020
Cellarius, Karen: 8010
Cepukenas, Emma: 7200
Cerino, Eric S: 1525, 4165, 4180, 4405, 4410, 7225, 7255, 7455, 8000, 8020
Cerino, Lauren: 7050, 7610
Cernohous, Inessah: 3450
Cerruti, Minyoung: 7155
Cervantes, Maria: 7320
Cetnarskyj, Roseanne: 8010
Cha, Hyungmin: 1015, 3245, 3370, 7040, 7500
Cha, Jun Hyung: 8015
Cha, Kyeongin: 8010, 8015
Cha, Seung Eun: 7720, 8000
Chaaya, Monique: 1155
Chachlani, Preeti: 7030
Chadwick, Darren: 1015
Chae, Hwa Young: 1140
Chae, Joosuk: 8010
Chae, Kacey: 7710
Chahine, Lana: 2510, 7640
Chai, Hye Won: 7735
Chakraborty, Ruchira: 7730
Chakravarty, Esha: 7020
Chakurian, Daphne: 8010
Chalise, Hom Nath: 4065
Chamberlin, Elizabeth S: 3110, 7135, 7490
Chamberlin, Emma: 2175
Chamberlin, Keran W: 2525, 7060, 7090, 7630
Chambers, Kelly: 4005
Chameli, Thomas: 7640
Chamut, Steffany: 4225
Chan, Alex Siu Wing: 7530
Chan, Angelique: 3290, 3545, 7145, 8015
Chan, Athena Chung Yin: 7005, 7265
Chan, Chin Pang Ian: 7510
Chan, Ding-Cheng (Derrick): 7275
CHAN, Francis KIN TUNG: 4195, 8015
Chan, Ka Po: 4195, 8015
Chan, Lili: 7630
Chan, On Fung: 3200, 8010
Chan, Sonia Kin Lai: 4050
Chan, Wai Chi: 7725
Chandler, Chelsea: 2355
Chandler, Julie: 8010
Chandler, Kelly D: 4280
Chandola, Tarani: 7640
Chandra, Tamir: 3325
Chandran, Aruna: 8020
Chang, Arthur: 7000
Chang, E-Shien: 1060, 1270, 2045, 4355, 7065, 7405, 8020, 8055
Chang, Grace: 7300
Chang, Han-Yun: 8015
Chang, Hui: 7355
Chang, Leah: 7010
Chang, Ling-Hui: 8010
Chang, Pei-Fen J: 7360
Chang, Po-Yen: 7400
Chang, Wen-Pin: 7205
Chang, Ya-Han: 7240
Chang, Yuanqing: 8000
Chang, Yu-Ping: 7245, 7360, 7510, 7695, 7910
Chang (Gudjonsson), Milan: 2040, 2240, 7305
Chanquin, Edward: 8020
Chanti-Ketterl, Marianne: 3280, 8015, 8020
Chao, Ka Kei: 7540
Chao, Linda L: 3010
Chao, Shiau-Fang: 7510
Chao, Shirley: 8015
Chapman, Brian G: 2175
Chapman, Chelsea: 2265
Chappell, Richard J: 4300
Chapple, Hilary: 2395
Charitou, Lauren Pizzulli: 7490
Charles, Cara I: 7000
Charles, Minard G: 2235
Charles, Susan T: 1350, 4180, 7030, 7255
Charlesworth, Georgina: 8000
Charlton, Judith L: 3455
Charness, Neil: 1040, 3205, 3480, 4130, 7360, 8000, 8020, 8020
Chasteen, Alison L: 1010, 7605
Chataut, Akankshya: 7485
Chaudhari, Diptaraj: 8000
Chaudhuri, Asish: 4170
Chaudhury, Habib: 1555, 3205, 4345, 7155, 7510, 7915
Chauhan, Shekhar: 3540
Chaves, Paulo HM: 3330, 7080, 7125
Chavez, Lorely A: 9050
Chavez Granados, Heily: 9020
Chavira, Laura: 7325
Chavkin, Nick: 3325
Chawrun, Isabella: 7630, 7905
Chaytor, Naomi S: 4515
Che, Sok Leng: 7540
Che, Yan: 7205
Chee, Wonshik: 3370, 7270
Chek, Carmen: 7455, 7540
Chekuri, Srujana: 7485
Chellappa, Rama: 8000, 8015
Chen, Amber X.: 7610
Chen, Annie T.: 2530, 8015
Chen, Bangwei: 7080
Chen, Brian H: 4170
Chen, Casandra WY: 8095
Chen, Chen: 8015
Chen, Chixiang: 1535, 3035, 3330, 4385
Chen, Chun: 7010
Chen, Chunfang: 8020
Chen, Diefei: 7225
Chen, Duan-Rung: 7035, 7460, 7925

Chen, Enna: 8000, 8020, 8020
Chen, Feinian: 3360
Chen, Feng: 1560
Chen, Feng: 8015
Chen, Frank Youhua: 8000
Chen, Gong: 3050, 7680, 8010, 8085
Chen, Haiying: 1390
Chen, Hongbo: 7305
Chen, Honglei: 2525, 4440, 7060, 7090, 7630, 8015
Chen, Hongxiu: 1355
Chen, I-Chen: 8015
Chen, I-Ling: 7080
Chen, Jen-Hao: 1540
Chen, Jiachen: 2160, 7735, 8010
Chen, Jiayu: 7515
Chen, Jingyi: 7710
Chen, Jinyu: 1000
Chen, Jufu: 1235
Chen, Ke: 7235, 7630
Chen, Ko-Xin: 8015
Chen, Kuan-Hua: 3285, 8040
Chen, Lei: 7510
Chen, Li: 7695
Chen, Liangchi: 7690
Chen, Lijing: 2465
Chen, Lili: 7425, 7930
Chen, Limei: 2370, 2470, 7095, 8010
Chen, Lin: 1195, 7300
Chen, Lingming: 3245, 7260, 7695, 7935
Chen, Liu: 8000
Chen, Li-Ying: 7070
Chen, Lu: 4030
Chen, Natalie M: 7040
Chen, Qiaoxi: 3135
Chen, Qingru: 8000
Chen, Qize: 7050
Chen, Ruijia: 1545, 2045, 8045
Chen, Shiyu: 8010
Chen, Shiyun: 7005
Chen, Shuangzhou: 1560
Chen, Siting: 3540, 7300
Chen, Siyu: 4310
Chen, Stella: 8055
Chen, Tuo Yu: 7150, 7270, 7735
Chen, Will: 1455, 4045
Chen, Xi: 2020, 2120, 3165, 3370, 7055, 7465, 7490, 7630, 7925, 8055
Chen, Xi: 7040
Chen, Xi: 7635
Chen, Xiaodong: 1365, 2500
Chen, Xiaotong: 7235
Chen, Xiayu Summer: 1115, 2265, 2355, 3030, 4270, 7135, 7155, 7505, 7540, 7660
Chen, Ya-Mei: 7510, 7525, 7915, 8010
Chen, Yanjun: 4300
Chen, Yan-Yan: 8000
Chen, Ya-Yun: 8020
Chen, Yi-An: 8010
Chen, Yi-Fan: 7080
Chen, Yi-Hua: 7735
Chen, Ying: 4550
Chen, Yishu: 4535
Chen, Yu-Chih: 4190, 7120
Chen, Yu-Chun: 7280
Chen, Yuling: 7215
Chen, Yunxiang: 8020
Chen, Zhengyi: 2015
Chen, Zhirui: 7045
Chen, Zijing: 2255, 2500, 7330, 7470
Chen, Ziyan: 8010
Chen, Ziyuan: 7255
Chen, Zizhuo: 7605
Cheney, Amanda: 3430, 7630
Cheng, Catherine J: 4310
Cheng, Clio Yuen Man: 1115, 7080, 7150, 7640, 7695, 7750, 8040
Cheng, Evien: 7010
Cheng, Greta Jianjia: 3375
Cheng, Hu: 8000
Cheng, Huai Y: 7430, 7610
Cheng, Jie: 7260
Cheng, Joyce: 7490, 7935
Cheng, Kent Jason G: 7470, 7925
Cheng, Mengling: 4455, 7535
Cheng, Mengyuan: 8010, 8015
Cheng, Qiyuan: 8010
Cheng, Rong: 1025
Cheng, Sheung-Tak: 8020
Cheng, Yu-Qi: 7110, 7400, 7490
Cheng, Yu-Shan: 7440
Cheng, Yutian: 7750
Cheong, Allen M. Y.: 7725
Chern, Alexander: 7140
Cherry, Debra: 8055
Cherry, Katie E: 2300, 2330, 7045, 7225, 8020
Chertkow, Howard: 7675
Cheslock, Megan: 8015
Chesney, Margaret A: 1245, 3010
Chess, Eric: 7495
Chesser, Stephanie A: 4000
Cheung, Ethan Siu Leung: 1105, 7420, 7455
Cheung, Pui Yin: 7230
Chew, Justin: 8015
Chhim, Srean: 8015
Chi, Nai-Ching: 7040
Chi, Peggy: 3170
Chia, Jia Qian: 8015
Chidebe, Runcie CW: 2445, 4000, 7350, 7475, 7600, 8065
Chien, Shao-Yun: 7465
Chih-Liang, Wang: 8000
Childress, Kristen: 8010
Chimehi, Bahareh: 3285
Chin, Janecca: 4050
Chin, Jessie: 4420
Chin, Laurie: 1325
Chin, Meejung: 8020
Chin, Savuth: 8015
Chin, Shu Yee: 1095
Chingui, Luciano: 8010
Chintapanyakun, Teepatad: 7205
Chiow, Jobina: 7100
Chippendale, Tracy: 8085
Chiriboga, David A: 3440, 3525
Chisholm, Latarsha: 8010
Chisolm, Cindy: 7210
Chitwood, Skyla: 1425
Chiu, Ching-Ju: 7610, 8015
Chiu, Chi-Tsun: 3290
Chiu, Chung-Yi: 7425, 7910
Chiu, Herng-Chia: 7670
Chiu, Wan-Yu: 3255, 8010
Chiu, Yu-Wen: 8020
Cho, Aeyoung: 7645, 7750
Cho, Al: 3080
Cho, Emma: 1290, 7145
Cho, Eunae: 7270
Cho, Eunhee: 7295, 7640, 7655, 7715, 7750
Cho, Hannah: 7145, 7630, 7665
Cho, Hyeonji: 8020
Cho, Hyeonmi: 7360, 7640, 7750, 8010, 8015
Cho, Hyunjin: 8000, 8010
Cho, Hyunjin: 8015, 8015
Cho, Jinmyoung: 4130, 4340, 7275, 7680, 8020
Cho, Joonyoung: 3040, 7030, 7420
Cho, Jungwon: 7655, 7715
Cho, Kyoungmin: 8000
Cho, S.M.: 8010
Cho, Seungjong: 2260, 7150
Cho, Seyoung: 7005
Cho, Tsai-Chin: 2515, 3145
Cho, Young I: 8090
Cho, Youngmin: 7535, 8035
Cho, Youngtae: 4070
Chodos, Anna: 1430
Chodosh, Joshua: 3010, 7465, 9020
Choi, Ahreum: 7275
Choi, Bokyoung: 7275
Choi, Boyun: 8015
Choi, Eunhee: 7275
Choi, Eunju: 7005
Choi, Eunyoung: 1015, 2040, 2460, 3125, 3245, 3540
Choi, Gayoung: 8045
Choi, Ha Young: 1570
Choi, HwaJung: 1450, 1495, 3145
Choi, Hwan: 8015
Choi, Hyojin: 7610
Choi, Jae Woo: 3545
Choi, Jean: 3095
Choi, Jeongeun: 8010, 8015, 8015
Choi, Jeungok: 8020
Choi, JiYeon: 7205, 7440, 7645, 7680
Choi, Jung-Yeon: 8015
Choi, Mi Sun: 8000
Choi, Minhyuk: 1545
Choi, Mona: 7360, 7750
Choi, Moon: 7155
Choi, Namkee G: 2520, 7080, 7655
Choi, S. Emily: 7350
Choi, Sang D: 8010
Choi, Sarah: 7660
Choi, Seo-Eun: 2020
Choi, Seongmi: 7205, 7440, 7680
Choi, Seongsu: 7430
Choi, Seo-Yun: 8000
Choi, Shinae L: 2420, 7495
Choi, Won: 3410
Choi, Y.Joon: 7030
Choi, Yejun: 8015
Choi, Yeon Jin: 1015, 3245
Choi, Yong Kyung: 7645, 8015
Choi, Yoon Saeng: 7705
Choi, Yoonseok: 7725, 8000
Chong, Weiguo: 7670
Chopik, William: 1570, 3405, 4525, 7455
Chosed, Renee: 7000
Chou, Kee Lee: 8010, 2520
Chou, Lin-Na: 1075
Chou, Pi-Ling: 7625
Chou, Wan Yun: 8010, 8015
Chou, Yu-Ting: 8010

Choudhury, Renoa: 7205, 8020
Choun, Soyoung: 7030
Chow, Bryan: 7750
Chow, Christine: 8020
Chow, Denise: 7270
Chow, Julian Chun-Chung: 7030
Choy, Jacky C. P.: 8020
Christensen, Janelle J: 3045, 4530
Christensen, Kaare: 7010
Christensen, Keri: 1065, 3510
Christian, Thomas: 2180
Christiansen, Cory L: 7155
Christopher, Emma: 7000
Christopher, Jennifer: 7480
Christopher, Jennifer M: 8020
Christopher, Theresa R: 7480, 8020
Chrzanowski, Brandi: 7440
Chrzanowski, Lauren K: 3385, 7065, 7425, 9015
Chu, Charlene H: 4175, 4465
Chu, Chien-Fang: 7510
Chu, Jing: 2465
Chu, Jun: 8010
Chu, Li: 1575, 4420, 8020
Chu, Sang Hui: 7205, 7440
Chua, Jolin: 1095
Chubinski, Jennifer: 8045
Chui, Hiu-kwan: 3200, 8010, 8035
Chui, M. K. Kirsten: 2530, 8000
Chukwuorji, JohnBosco C: 4000, 7150
Chulak, Jamy: 2325, 7440
Chun, Gloria Ka Ming: 7460
Chung, Edwin Ka Hung: 4390, 7745, 8000
Chung, Jane: 4205, 4420
Chung, Misook: 7005
Chung, Ray-Yuan: 8015
Chung, Seohyeon S: 7275
Chung, Soondool: 1140, 3440, 8000
Chung, Thomas: 8010
Chunga, Richard E: 7040, 7065, 7660, 8020
Chung-Do, Jane: 8020
Chur, Marcus: 7640
Churchill, Linda C: 7260
Chusyd, Daniella E: 8000
Cicero, Caroline: 2440
Cicero, Ethan: 2015, 7100
Cichy, Kelly E: 4280
Cidav, Tom: 7200, 7260
Ciemins, Elizabeth L: 3215
Ciesla, Marissa: 7635
Cieslik, Katarzyna A: 1420, 8000
Cigliana, Jill: 8020
Cigolle, Christine: 7320
Cihlar, Volker: 1100
Cilluffo, Silvia: 7245
Cimarolli, Verena R: 7040, 7065, 7135, 7660
Ciolfi, Mary Lou: 3090
Cipher, Daisha: 1105, 7265
Ciro, Carrie A: 7330
Cirone, Victoria: 7720
Clair, Catherine: 2230, 8000
Clancy, Erin: 8000, 8020
Clapham, Charlotte: 7620
Clapp, Joshua: 7440
Clardy, Aisling: 1145
Clari, Marco: 7245
Clark, Alexandra L: 1495, 2045
Clark, Carolyn: 4060
Clark, Jeanne M: 7710
Clark, Kate: 3170
Clark, Melissa: 3535
Clark, Valerie: 3240
Clarke, Heather: 3215
Clarke, Philippa: 1045, 1115, 3540, 7140
Clay, Olivio: 4490, 8020
Clayton, Jordana: 3500, 7425, 7905
Clayton-Jones, Kate: 2360
Cleland, Claire L: 3125
Clem, Sarah: 7020, 8010
Clevenger, Carolyn K: 1125, 2295, 7220, 8015, 8020
Cleverdon, Amy C: 4045
Click, Mary V: 8010
Clifford, Adeline: 2130
Cline, J. Mark: 3045
Cline, Krista: 7420
Cloud, Leslie: 8010
Clouse, Kate: 9050
Cloyes, Kristin G: 2395, 4330
Clutter, Jill E: 7265
Coakley, Amanada: 4235
Coates, Martha C: 7020
Coats, Heather: 7065
Coats, Jacquelyn: 3235
Cobb, Ryon J: 3180
Coccia, Kathryn W: 4080, 7520, 7545, 7905
Cochran, Josh: 7735
Cochrane, Barbara: 4470
Coco, Laura: 7455
Coe, Norma B: 7000
Coelho, Luisa: 7310
Coen, Paul M: 1240, 1410, 2080, 2220, 4310, 8020
Coffman, Cynthia J: 2145, 8015
Cofie, Leslie: 7530
Cogliati, Tiziana: 1035, 4310
Cohen, Alan A.: 7010, 8020
Cohen, Ann D: 3375, 7720
Cohen, Beth: 8010
Cohen, Deborah: 4160
Cohen, Emma: 7350
Cohen, Joshua E: 2045, 2315, 3250
Cohen, Marc: 4305, 7670
Cohen, Steven A: 4105
Cohen, Susanna: 1565
Cohen Quay, Lisa: 3200
Cohn-Schwartz, Ella: 3530
Coker, Karen: 8015
Coldiron, Allyson M: 7750
Cole, Rebecca A: 8040
Cole, Steve W: 1060
Cole-Hunter, Thomas: 3125
Coleman, Carissa: 1530, 7635, 7650
Coleman, Rachel: 2265
Coletta, Giulia: 4260
Coley, Yates: 3010, 4230
Collette, Christopher: 7285
Colley, Alexis: 4045
Collier, Natasha: 7490
Collins, Amanda J: 3075
Collins, Amber: 1070
Collins, Sandra K: 7425, 7910
Collins-Pisano, Caroline: 7640, 8020
Colobong, Romeo: 4445, 7040, 7055
Colombowala, Fatema: 1020, 4120
Colopietro, Kelley J: 7155, 7435
Colverson, Aaron: 4285
Colvin, Meaghan E: 1185, 2130
Comer, Cozette: 8010
Comery, Alastair: 2365
Como, Dominique H: 8015
Compernolle, Nell: 3405, 8010
Condon, Gerald: 7630
Coney, Rachel: 4510
Cong, Zhen: 7045
Cong Vu, Nguen: 7265
Congedo, Benjamin: 2345
Conklin, Jamie: 1465, 3300
Connell, Cathleen M: 3145
Connelly, Denise: 4270, 7510
Conner, Laneshia: 3275
Conner, Norma: 8015
Conniff, Joshua: 7425
Connolly, Maureen: 4235
Connolly, MT: 2150
Connor, Dylan: 8000
Conroy, David E: 1545, 8020
Contreras, Cameron M: 4325
Conway, Mike: 1485, 7145
Conwell, Yeates: 8035
Cook, Diane J: 2360
Cook, Paul: 7360
Cook, Wendy: 1365
Cook-Daniels, Loree: 4495
Cooke, Alicia K: 7445, 7935
Coombs, Lorinda A: 8035
Coon, David W: 2190, 4320, 7260
Cooney, Alissa M: 2535
Cooper, Brittany: 2180
Cooper, Julie B: 7720, 9020
Cooper, Kalvry: 1185
Cooper, Lujira: 4445
Cooper, Tara R: 8010
Cooper-Williams, Jasmine: 1075
Cooper-williams, Jasmine K: 8065
Cope, Marie T: 2300
Corazzini, Kirsten: 2000, 2380
Cordes, Dietmar: 8055
Coresh, Josef: 3570
Corley, Robin P: 4095, 7225
Corley, Robin: 8020
Corley, Samantha S: 4185
Corman, Jennifer: 7030
Corneau, Emily: 4460
Cornelison, Laci: 3090
Cornelius, Talea: 1345
Cornell, Porita: 3535
Corner, Lynne: 7035
Coromac-Medrano, Juliamaria: 7225, 7500
Correa, Seneca: 2370
Cortes, Constanza: 8000
Cortes, Tara: 8020
Cortes, Tiffany M: 2505
Cosco, Theodore D: 7720
Cosentino, Stephanie: 1025
Cossett, Benoit: 7030
Costa, Amy N: 1135, 1405, 3055, 7500, 7610
Costa Grille, Alba Maria: 3150
Costantini, Helena: 4355
Costello, Kathryn: 7125
Cotten, Shelia: 2355
Cotter, Valerie T: 8000, 8015

Cotton, Quinton: 7640, 7685
Cotton, Samantha G: 2340, 3175, 4470, 7340, 7730
Couch, Elyse: 3500, 7200, 8000
Coughlin, Joseph: 1095, 3245, 7050, 7065, 7090, 7610
Coulourides Kogan, Alexis M: 2495
Couper, David: 2525
Covington, Amy: 7315
Covinsky, Kenneth e: 4480
Cowen, Nannette: 7065
Cox, Brian S: 3240
Cox, Laura: 8000
Cox, Mary A: 8020
Cox, Simon: 3325
Cox, Susan M: 4435
Coxon, Selby: 3455
Coyle, Caitlin: 7490
Cozad, Aisha B: 3015, 7125
Craft, Suzanne: 2235, 7425
Craig, Taylor: 2230
Cram, Peter: 2090
Crampton, Alexandra: 3365, 9045
Crane, Paul K: 3065, 7320
Cranley, Lisa: 4175, 4465
Crawford, Christina: 8015
Craypo, Lisa: 7200
Creevy, Kate E: 8000
Crenshaw, Kirsten: 8010
Crenshaw, Shannon M: 8010, 8015
Crespo, Eric L: 3325
Crews, Deidra C: 2010, 3150, 4420
Crimmins, Eileen: 1455, 2040, 3095, 8060
Crisafio, Mary: 7020
Crisci, Alex: 7610
Crittenden, Jennifer: 2265, 7680
Crocker, Andrew: 1065
Crocker, Jillian: 7460
Croff, Raina L: 3140
Cronin, James: 8020
Cronin, Teresa: 8000
Crook, Jennifer M: 2300, 3055, 7025, 8015, 8020
Crossdale, Rachel: 8010
Crouch, Adele: 8020
Crouch, Elizabeth: 4105
Croucher, Madison: 8015
Crouter, Scott: 7005
Crowder, Jolie: 2280
Crowder, Victoria R: 8035
Crowe, Alyssa: 7020
Crowe, Michael: 1320, 7700
Crowley, Julia: 4475
Cruz, Grace Trinidad: 7205
Cruz, Michelle: 1575
Cruz, Roberta J: 2305
Cruz Erisman, Morgan: 2560
Cruz-Almeida, Yenisel: 1445
Cruz-Carrillo, Yesenia: 4065, 4500
Cryer, Anthony Q: 3140
Crystal, Stephen: 7110
Csallner, Christoph: 7155
Csikai, Ellen L: 7055
Cudjoe, Thomas: 1225, 2010, 2230, 2520, 4190, 4220, 4265, 4520
Cuevas, Heather: 7440
Cuevas, Nicolas: 7660
Cuffaro, Taylor: 1530
Cui, Saishi: 7620
Cui, Xiaoyan: 9000
Cui, Xirong: 8010, 8015
Cummings, Caroline: 9015
Cummings, Maya Rockeymoore: 1180
Cummings, Stephen: 2220, 2510
Cummings, Steve: 1090, 1435
Cummings, Steven R: 2080, 8020
Cummings-Vaughn, Lenise: 8030
Cummins, Phyllis: 7350, 7475
Cunningham, Solveig A: 3530, 7060
Curci-Delgaro, Kathy: 3240
Curiel, Rosie: 8010
Curl, Angela L: 7160
Curl, Angela: 8085
Curran, Sean: 8000
Curriero, Frank C: 3375
Curriero, Samantha: 2010, 3150
Curtan, Shelley: 3340
Curtis, Ashley F: 1135, 1405, 3055, 3360, 7500, 7610
Curtis, David S: 7230, 7420, 7455
Curtis, Laura M: 7630
Curyto, Kimberly: 1070, 1080, 3240, 7695
Cusack, Meagan: 7290
Cutforth, Asia: 7700, 7720, 7935
Cvejkus, Ryan: 2220, 7060, 7625
Czaja, Sara J: 1040, 4130, 4340, 4355, 4370, 7040, 7065, 7080, 7360, 7405, 8020
Czyz, Rebecca: 7405

**D**

Da, Wendi: 4365, 8020, 8050
da Costa Pinaffi Langley, Ana Clara: 8015
da Luz Scherf, Erick: 8000
da Rosa, Grace: 7610
da Silva Pantoja, Patricia: 8000
Düzel, Sandra: 7030, 7210
Dabelko-Schoeny, Holly: 2070, 2195, 3080, 3485
Dabiri, Sanaz: 8000
Dabrowski, Barbara S: 7440, 7490
Daddato, Andrea: 2085
Dadgostar, Porooshat: 2430
Dadson, Deborah A: 7455
Dagne, Mahederemariam: 1300
Dahal, Arati: 7080
Dahal, Roshani: 7080
Daher-Nashif, Suhad: 1155
Dahlqvist Leinhard, Olof: 4310
Dai, Lu: 7060
Dai, Shuting: 8050
Dai, Xiaochen: 7040
Daliparthi, Meghana: 9025
Dalkin, Sonia: 4255
Dallo, Florence: 1045
Dalmer, Nicole: 7750
D'Alonzo, Anthony: 8010
Dalton, Jarrod: 7235, 7690, 8000
D'Ambrosio, Lisa: 1095, 7050, 7065, 7090, 7610
D'Amico, Danielle: 7675
D'Amore, Cassandra H: 7655, 7720
Dams-O'Connor, Kristen: 3250
Dang, Hope: 7010
Dang, Kristina: 2040
Dang, Linh: 3540, 7490, 7695
Dang, Stuti: 2545, 7360, 7675, 7730, 8010, 8070
Dangeti, Reshma Sahithi: 7360
Dangler, Kevin: 4535
Danial-Saad, Alexandra: 8010
Daniel, Gaea: 4165
Daniel, George: 8020
Daniel, Kathryn: 1105, 7265
Danielsen, Chris: 2395
Danielson, Michelle E: 1410
Danilovich, Margaret: 8020
Dannefer, Dale: 1180
Dansereau, Lisette: 4450
D'Antonio, Patricia: 2390, 3260, 3390
Dao, Elizabeth: 1535, 3335
Dao, Jonathan: 7530
Daquin, Jane C: 4060
Darby, Kevin P: 7225
Dark-Freudeman, Alissa: 2325
Darmann-Finck, Ingrid: 2140
Das, Papiya: 2050
Das Bhattacharjee, Sreyasee: 7360
Das Gupta, Debasree: 1235, 7355
Dash, Atreya: 7305
Dassel, Kara B: 3465, 3500, 4520, 7425, 7905, 8020
Dauenhauer, Jason: 2265, 7155
Daum, Christine H: 7630, 7905
Daus, Marguerite: 4460, 8010
Dautovich, Natalie: 7725, 8010
Davatzikos, Christos: 2510, 7015
Dave, Chintan: 8010
Davey, Adam: 3550, 7700
David, Daniel: 1395, 8045
Davidson, Leslie F: 3300
Davidson, Patricia: 7440, 7500
Davidson-Turner, KJ: 3235
Davie, Laura: 2000
Davies, Katharine: 1115
Davila, Heather: 1395
Davis, Andra: 7065
Davis, Carter H: 7235, 7480, 7900
Davis, Ian: 1390
Davis, Jennifer C: 1365, 8015
Davis, Kimberly D: 4470
Davis, Lewis: 1070
Davis, Rachel: 8055
Davis, Scott A: 1520, 8010
Davis, Scott: 8020
Davis, Vincent: 4340
Davis Chilton, Patricia: 8020
Davis-O'Reilly, Cynthia: 4160
Davitt, Joan K: 8010

Davlyatov, Ganisher: 1550, 3255
Davoudi, Anis: 3070
Dawson, Bonnie: 8015
Dawson, Deirdre R: 1205
Dawson, Walter D: 4235, 7440, 8010, 8010, 8020, 8020
Dayama, Neeraj: 7510
Dayley, Amber: 7085
Dayrit, Jessika G: 7490
De, Supriyo: 7210, 7305
de Albuquerque, Lidio L.: 8015
de Boer, Bram: 4345
De Filippi, Francesco A: 7275
De Fries, Carson M: 1185, 4115, 7495, 8020
De Jonge, Karl E: 7640, 8020
De Klerk-Rubin, Vicki: 7250
De Lima, Bryanna A: 3045
De Man, Ruben: 4170
de Medeiros, Kate: 7055
de Paula Couto, Clara: 1375
de Rosa, Cristina: 7245, 7695
De Sanctis, Pierfilippo: 8000
De Vito, Alyssa: 7040
de Vries, Brian: 2540, 8080
De Vries, Erica: 4255
de Vries, Tjard: 7480
de Wit, Emma J: 2255
Deal, Jennifer A: 1055, 2035, 2525, 4300, 4440, 8015
Dean, Devlon: 1530
Dean, Olivia: 1085
DeBell, Karen E: 7695
DeCarli, Charles: 4430, 7500, 7930
Deck, Monica M: 3065
Decker, Autumn E: 1190, 2180, 2325
Deckert, Amy: 7325
DeConne, Theodore M: 4005
Decosimo, Kasey: 7685, 8015
Deelen, Joris: 8015
Deer, Rachel: 7400, 7920, 8015
DeFrancesco, Erica: 1345
Degenholtz, Howard B: 2480, 3255, 7110, 7510, 7545, 7915
DeGennaro, Jennifer: 8010
DeGroot, Lyndsay: 4370
Deichert, Jerome: 7510
Deif, Reem: 1155
DeJohn, Amber: 4430
Dekel, Tal: 3015
Dekhtyar, Serhiy: 3035
Del Bene, Victor: 7285
Delaney, Erin: 7055
DeLange Martinez, Pauline: 8010
DeLany, James P: 2505, 8020
Delaporte, Magdalena: 7455, 7925
DeLaTorre, Alan: 7410
Delgado, Ferdinand: 8015
Delgado, Kimberly: 7510
Delgado, Roxana E: 2295
Delgado, Sara: 3575
Delgado-Ortiz, Laura: 2170
DeLiema, Marguerite: 1370, 2150, 3270
Delitto, Anthony: 7330
Dellefield, Mary E: 8075
Deller, Juergen: 7050
DeLongis, Anita: 4405
DeLouize, Alicia M: 8015
DeMarchis, Emilia: 4160
DeMarco, Mari L: 7720
Demir, Murat: 8070
Demircan, Kamil: 7210
Demirer, Ibrahim: 1285
Demiris, George: 1290, 2085, 4220, 7145, 7270, 7665
Demuth, Ilja: 7030, 7210
den Ouden, Mirre D: 4255
Denfeld, Quin E: 7440
Denham, Michael W: 7140
Denison, Paige: 2450
Dennis, Elizabeth A: 7315, 7720
Denno, Benjamin: 4235
Denson, Kathryn: 7480
Denson, Steven: 7480
Dent, Elsa: 2230
Dent, Kallisse R: 7695
Derboghossian, Grace: 7015
Derby, Carol A: 2160
Derenne, Nicole M: 8020
DeRoo, Scott: 7625
Dertinger, Melinda: 2395
Desai, Smit: 4420
Deshmukh, Madhyami: 2255
Desir, Marianne: 2545, 8070
Desjardins, Michael R: 2045, 3375, 4430
Desoria, Maureen G: 7080
Deters, Shana: 7720
Deturbide, Katherine: 3170
Devereaux Melillo, karen: 2440
Devi, Reena: 4255, 7295
DeVincentis, Diane: 8000
Devone, Frank: 4460, 8010
Dewansingh, Neha: 7130
Dews, Denise: 3300
Dey, Judith: 4305
Dey, Nutifafa E Y: 1450, 4065, 4500
Dey, Venance: 4175
Dhakal, Usha: 8020
Dhaliwal, Ravneet: 1020
Dhanekula, Arjune: 7625
Dhar, Nirmala: 8010, 8020
Dharia, Aastha K: 8010, 9050
Dhawan, Aanika: 8000
Dhingra, Radha: 7625
Dhoble, Abhjieet: 3135
Di Bona, Vito: 3010
Di Gessa, Giorgio: 1290, 2365, 3125
Diallo, Idiatou: 3070, 7615
Dian, Larry: 1365
Diaz, Jose A: 7455
Diaz, Joseph: 3115
Diaz, Monica: 8020
Diaz-Asper, Catherine: 2355
Diaz-Valdes Iriarte, Antonia: 3035
DiCamillo, Mark: 7015
DiCarlo, Nicholas: 4025
Dickerson, Anne E: 3185, 7160
Dickinson, Karen: 7650
Dickson, Victoria: 7465
Dieckmann, Nathan F: 7020, 7205
Diederich, Freya: 3550
Diedrich, Jolene: 7425
Diegel, Morgan: 7010
Diehl, Keegan: 4435, 7065
Diema Konlan, Kennedy: 7080
Dieterk, Kristi: 7000
DiFranco, Lacey: 7465, 8020
Dikhtyar, Oksana: 4510, 7295, 7435
Dilanchian, Andrew: 3060
Dilian, Omer: 1435
Dillon, Ellis: 4045, 7740
Dillon, Kayla: 7200
DiMaria-Ghalili, Rose Ann: 7020
Dimeo, Christina: 3460
Dimonte, Valerio: 7245
Dimri, Vrinda: 7455, 7720
Dindo, Lilian: 7205
Ding, Ding: 9000
Ding, Jun: 9015
Ding, Kedong: 7055
Ding, Kehong: 7210
Ding, YaPing: 7520, 7910, 9045
Ding, Yong: 1355
Dionne, Laura A: 1530
Dissault, Nicole: 2180
Distefano, Giovanna: 4310, 8020
DiVall, Sasha: 8015
Divecha, Ayushi: 3250
Dixon, Mandy: 3060
Dixon, Saharra: 2555
Dixon-Gordon, Katherine: 7255
Do, Jonghwa: 8020
Doan, Andrew: 7210
Doan, Lan N: 8045, 8095
Dobalian, Aram: 7045
Dobbs, Debra: 1360, 2095, 2185, 2370, 3365, 3495, 7435, 7455, 8010
Dobish, Heidi B: 4040
Dobrota Lai, Sylvie: 8015
Dodd, Elvira: 7405
Dodge, Hiroko H: 7005, 8000
Doerfler, Deborah: 8015
Doh, Michael: 7480, 7680
Dohan, Daniel: 3175, 4450
Doheny, Megan: 7215
Doherty, Aiden: 3050
Doherty, Fiona: 2195
Dokshina, Darya: 2045
Dolan, Hanne R: 7260
Dolansky, Mary A: 1490
Dolbin-MacNab, Megan L: 1190, 7005, 7265
Dole Center of Excellence, VA Elizabeth: 8010
Doll, Johannes: 3155
Domingo, Divine C: 7150
Dominguez, Briana Alejandra: 8025
Dominigue, Benjamin: 4550
Domnick, Hallory: 7265, 7460
Domoto-Reilly, Kimiko: 1480, 2530, 7200
Donahue, Patrick T: 2045, 3375
Dondero, Kathleen R: 8015
Donega, Stefano: 7210, 7305
Donelan, Karen: 1230
Donelle, Lorie: 4270
Dong, Bigrong: 8015
Dong, Fanghong: 8020
Dong, Jingyi: 2125
Dong, Sihan: 1245
Dong, Wuyi: 7070, 7235, 7350, 7735
Dong, Yanjun: 7155, 8010

Dong, Yu: 1535, 3035, 7440
Dong, Yue: 3480
Donkor, Michael: 8000
Donofry, Shannon D: 4315
Donohoe, Maria: 2185, 7465, 7685
Donorfio, Laura KM: 1185, 2175, 2325
Donovan, Heidi: 4530
Donovan, Nancy J: 4180
Dooley, Erin E: 7720
Dopson, Rasheera: 8000
Dorame, Ashley: 4375
Doran, Kelly: 1070
Dore, Peter: 1275
Doriscar, Jonathan E.: 8080
Doriwala, Hussain: 7750
Dorn, Adrianah: 7200
Dorr, David A: 4160
Dorris, Jennifer: 4540
Dosa, David M: 2185
Dose, Ann Marie: 4415
Dosso, Jill A: 2565, 7430
Dotolo, Danae: 7530, 7905
Dotson, Vonetta M: 1205, 2475
Dougherty, Maura: 8010
Dougherty, Ryan J: 3070
Douglas, Joseph: 7730
Douglas, Natalie: 1160
Dowd, James J: 1300
Dowden, Lauren: 1125
Dowling, Maritza: 3190
Dowling, N. Maritza: 2160
Downer, Brian: 1075, 1170, 1320, 4550, 7230
Downing, Kimberly: 4505
Doyle, Cassidy: 8000
Doyle, Margaret F: 2160, 7735, 8010
D'Oyley, Heather: 7750
Dozier, Mary E: 3575, 4120
Dozier, Stephen: 8000
Drabo, Emmanuel F: 7470, 7905
Draeger, Dagmar: 7110
Dragan, Deanna: 4330, 7695, 8015
Drake, Connor: 7685
Drakes, Josiah: 8015
Draughn, Melissa: 4265
Dravecz, Nikolette: 3060
Draw, Kandis: 4505
Dremann, Andrea: 3175
Drewelies, Johanna: 1350, 7030, 7210
Driscoll, Monica: 7010
Driver, Charles: 4185
Driver, Jane: 8020
Driver, Jane "Jenny": 3425
Drossel, Claudia: 3110
Drost, Jennifer: 7440, 7475, 7900
Drumheller, Jake: 7315
Drummond, Neil: 8010
Dryden, Eileen: 2430, 8010
Du, Pang: 4110
Du, Shufa: 7275
Du, Xuan: 7670
Du, Yan: 8015, 8050
Duan, Hongzhe: 7075, 7210, 7715
Duan, Liping: 7320
Duan, Siyu: 2255
Duan, Yanjun: 4465
Duan, Yinfei: 4100, 4195, 4245
Dubé, Catherine: 8015
Duberstein, Paul: 1380, 7110
Dublin, Sascha: 3010, 4230
Dubose, Kline: 4520
Dubowitz, Tamara: 8015
Dubure, Emmanuel Banbur: 7145
Duchesne, Simon: 7720
Duchow, Olivia: 7205
Duchowny, Kate: 1240, 7350
Duckett, Jacquelyn: 7265, 7735
Duckworth, Alyssa C: 7310
Dueck, Kimberly: 7660
Duehring, Jasmin: 8015
Duff, Kevin: 7200
Dufour, Alyssa B: 2080
Dugan, Elizabeth: 1305, 1405, 3265, 7065, 7135, 7240, 7635, 7695, 7700
Duggan, Katherine A: 1525, 7030
Dukkipati, Haripriya: 2180
Dunavan, John: 1265, 7075
Dunbar-Jacob, Jacqueline: 2140
Dunkle, Ruth: 7420
Dunlap, Ben: 7545
Dunlap, Janette: 7320
Dunlap, Shawn: 2345
Dunnigan, Allison: 7155
Dunsmore, Victoria J: 7610
Dupre, Matthew: 7625
Dupuis, Sherry L: 7055
Dupuy, Emma G: 4490
Duque, Gustavo: 1330, 1580, 2350, 8020
Duque Urrego, Daniel: 7205
Durkin, Maeve: 7205
Dusetzina, Stacie: 3075
Duthie, Edmund H: 2535, 7480
Dutta, Angshita: 7075
Dutta, Chhanda: 2435
Duty, Charlotte: 7215
Dwibedi, Aditya Kumar: 7360
Dwyer, Johanna: 8015
Dye, Maya E: 8035
Dyer, Jamie R: 4040
Dys, Sarah: 1230, 3535, 8010, 8020
Dzwierzynski, Jenna: 8000

**E**

Eagle, Angelica: 7440, 7660
Earles, Julie L: 7225
Easter, Molly: 8000
Eastman, Jennifer: 8010
Eaton, Charles: 4515, 7040
Eaton, Jacqueline: 3465, 4040, 7085
Eberhardt, Kenda: 1135
Ebulum, Genevieve: 7150
Eby, David W: 3455
Echevarria, Klarisse: 7000
Eckstrom, Elizabeth N: 3045
Edahiro, Ayako: 7350
Edelman, Linda S. S: 4520, 8020
Edelstein, Barry: 7115, 7490
Edison, Jos: 7275
Edland, Steven D: 8015
Edwards-Childs, Paulita L: 2480, 3100
Efird-Green, Lea: 1520, 2105, 8010
Ehlers, Diane: 3285
Ehrlich, Joshua R: 1115, 2525, 4380, 4455, 7035
Eich, Teal: 8000
Einstadter, Douglas: 8000
Eisen, Howard: 7620
Eisenberg, Chase: 1505
Eisenstein, Amy R: 2295
Eisikovits, Zvi: 3015
Ejaz, Farida K: 1145, 3340, 7405
Ejiri, Manami: 7205, 7540
Ek, Mary: 7465
Ekerdt, David J: 1375, 4330
Eklund, Nicole M: 4315
Ekoh, Prince C: 8010
El Asmar, Khalil: 8020
El Sammak, Aya: 1155
Elayoubi, Joanne: 7070
Eldadah, Basil: 1340
Eldridge, Corinne: 8025
Elfenbein, Pamela H: 3450
Elftmann, Hanley: 7110
Elhassan, Mohamed: 8015
El-Hay, Tal: 8015
Elhussein, Yassein: 8015
Elias, Maya N: 7735
Eliatamby-O'Brien, M.: 8050
Eliopoulos, Elaine M: 3015
Elisha, Victor: 7740
Elkind, Mitchell S V: 8085
Ellington, Lee: 4330, 4520, 7085
Elliot, Amy E: 3380, 7295
Elliot, Christian: 7005, 7135
Elliott, Emily: 7045
Elliott, Lauren: 1335, 3385, 4435, 7065, 7425, 9015
Elliott, Meghan R: 7030
Elliott, Susan J: 4470
Ellis, Erica J: 4510
Ellis, Jennifer L: 2455
Ellis, Julie: 7070, 7425, 7540, 7930
Ellis, Katrina R: 1060
Ellis, Sydney: 7345
Ellisman, Mark: 7015
Ellison Keeler, Carlyn: 8020
Elman, Alyssa: 4355, 7405
Elmasri, Jeannine: 7425, 7905
Elo, Irma T: 9035
Elvevåg, Brita: 2355
Emery-Tiburcio, Erin: 4470, 7650
Emezue, Chuka N: 3500
Emlet, Charles A: 4495
Emrich, Christopher: 2300, 7025, 8015, 8020
Emrick, Lauryn: 7440
Eng, Janice: 7720, 8015
Engdahl, Bo L: 2425
Engdau, Shelly: 8010
Engeland, Christopher G: 1250, 7210, 7455, 8020
Engell, Katia: 7055
Engelman, Michal: 3225
Engelmann, Corinne D: 4300
Engle, David W: 4100
Engleman, Michal: 3225
English, Tammy: 3355
Enguidanos, Susan: 4080, 8010
Enroth, Linda: 2460
Ensrud, Kristine: 4055, 4385, 7630
Enterria-Morales, Daniel: 7210
Entwistle, Amy: 8000
Eo, Yugyeong: 3545
Epps, Fayron R: 2295, 4175, 7005, 7700
Epstein, Cynthia: 1380

Epstein, Mara M: 7060
Epstein-Lubow, Gary: 1260, 1270, 2305
Eramudugolla, Ranmalee: 3185
Eraslan, Emre: 8010
Erb, Isabelle: 8010
Erdman, Elizabeth C: 7495
Erickson, Alexander J: 2345, 7660, 7735
Erickson, Kirk: 4315
Erickson, Lance: 2385
Erickson, Olivia: 8015
Erkens, Petra: 7685
Erker, Jayma: 7075
Erlandson, Kristine M. M: 7285, 8015
Ermolenko, Mia: 8035
Ernst, Joy S: 7405
Ernsth Bravell, Marie: 8015, 9015
Ernstsen, Linda: 7005
Ersek, Mary: 1160, 4195, 8015
Ertle, Emilee: 7660
Erus, Guray: 7015
Erving, Christy L: 3235, 3475
Escorcia Encarnación, Carolina: 7400, 7920
Esiaka, Darlingtina K: 1465, 1495, 3220, 3475, 4000, 8015
Espejo, Kimberly: 8015
Espejo, Luis: 7010
Espeland, Mark A: 1390
Espinoza, Sara: 1460, 8015
Espinoza, Sara E: 1410, 1460, 2505, 4210
Esposito, Michael: 3375, 4035, 7350, 8010
Esser, Karyn: 8000
Essien, Ime: 8000, 8015
Estabrooks, Carole A: 4100, 4195
Estapé, Jorge: 2445
Estapé, Tania: 2445
Estes, Katie L: 7425, 7930
Estlein, Roi: 1565
Estrada, Leah: 4480
Eun, Heesoo: 8015
Eustice-Corwin, Alexander: 4400
Evans, Michele: 1250, 2055, 2330, 8000
Evans, Teairra: 8020
Evans, Tracy L: 4340
Evans, William J: 4260
Evelyn-Gustave, Allyson: 4420
Everson-Rose, Susan A: 1265
Eymundsdottir, Hrafnhildur: 2040
Eze, John E: 7150
Ezeihuoma, Obinna: 7150
Ezeokonkwo, Francesca C: 2460

F

Fabbre, Vanessa: 4250
Fabius, Chanee: 3105, 3570, 7700
Factora, Ronan: 7405, 7920
Fadalla, Carol: 3385
Fadel, Meghan: 3100, 3430
Fagundes, Christopher P: 3285
Fahr, Bruce T: 8000
Fain, Mindy: 8015
Falcão, Deusivania Vieira Da Silva: 8015
Falck, Ryan S: 1365, 1535, 3335, 7030, 8015
Fallahi, Afsaneh: 7315
Falvey, Jason: 1075, 1535, 3035, 4385, 8010, 8065
Falzarano, Francesca: 1165, 2305, 7660, 8010, 8020
Famula, Jessica: 2200
Fan, Baoyu: 3330
Fan, Erica K: 3375
Fan, Jason: 2395
Fan, Jiayao: 8015
Fan, Yujun: 8075
Fang, Chao: 2365
Fang, Estelle Xun: 7460
Fang, Fang: 1485, 7145
Fang, Mei: 3060, 8010
Fang, Mei Lan: 1015
Fang, Ya: 1365, 2255, 7305
Fang, Yu: 4270
Fang, Yuan: 8015
Fanning, Jason: 2505, 7710
Farago, Fanni: 3015
Farina, Crystel L: 8015
Farina, Mateo: 1280, 2515, 3030, 3095, 3370, 4400, 7040, 7485, 7925, 8060
Farmer, Heather R: 3075, 4015
Farmer, Justin: 3035, 4440
Farmer, Matthew S: 8015
Farnham, Trish: 2500
Faro, Jamie: 8015
Farook, Jahoor: 2235
Farrants, Kristin: 2365
Farrell, Tim: 4520
Farris, Kristen M: 8000
Farsijani, Samaneh: 7060, 7710, 8015
Fasching Maphis, Christine: 2460
Faselis, Charles: 9025
Fassi, Janelle E: 1185, 2130, 7265, 7490, 7670
Fastame, Maria Chiara: 1235
Faucheux, Gavin R: 7225
Faul, Anna C: 4470, 7340, 7730
Faul, Jessica: 8060
Fauth, Elizabeth B: 4335, 4435, 7030, 7065, 7270, 7325, 8020
Fawcett, Janet: 8015
Fazio, Sam: 2105, 3000, 3000, 4125, 4200, 7465
Fears, Vanessa: 4475
Featherston, Kyle G: 8010
Featherstone, Katie: 7520
Federman, Alex: 3250, 7630
Fegter, Ollie: 9040
Fehland, Jess: 3425
Fehnel, Sheri: 8020
Fei-Fei, Li: 1575, 4420
Fein, Taylor: 8000
Feldman, Sid: 4445
Feldman, Sue: 3255
Feliciano, Leilani: 7695, 7720, 8020
Felix, Cynthia: 4490, 9000
Felt, John M: 4180
Felts, Adam: 7065, 7610
Feng, Derek: 2020
Feng, Jeff: 7360
Feng, Yali: 2355
Fennell, Charles: 3140
Fenstermacher, Erika A: 7115, 7490
Fenton, Laura: 7495, 7930
Ferdows, Nasim: 4105, 8010, 8015
Ferguson, Anna M: 8000
Ferguson, Dee: 7225
Ferguson, Dian: 3140
Ferguson, Erin: 8015
Ferguson, Giselle A: 4185
Ferguson, Lee Ann S: 1450
Ferguson, Melissa M: 2065
Fernandes, Cecilia: 7660
Fernandez, Mariana: 4050
Fernandez, Otniel: 4420
Fernandez Cajavilca, Moroni: 7085, 8045
Ferraro, Kenneth: 3405, 7535, 8020
Ferreira, Kyra: 8015
Ferreira, Pamela: 9000
Ferrell, Anastasiya: 7440
Ferreri, Jeff: 4490
Ferretto, Marianella E: 7695, 7935
Ferri, Christine: 2275, 7235
Ferrie, Joseph P: 4375
Ferrucci, Luigi: 2510, 4170, 7010, 7210, 7305, 7710, 7720, 8000, 8000, 8000, 8015, 9015
Festus, Eniola Z: 3235
Fete, Zoe F: 1030
Feyh, Lauren: 7265
Fick, Donna M: 2140, 7410, 7645
Fiehn, Oliver: 8000
Fielding, Roger: 2005, 2380, 8015
Fields, Beth: 1455, 2085, 2190, 2415, 4530, 7270
Fields, Bronwyn: 2200
Fields, Cynthia D: 4100
Fields, Eve: 1535
Fields, Maizonne: 8015
Fields, Noelle L: 1105, 2195, 7005, 7265, 8010
Fields, Torrie: 2390
Fifield, Alexis: 7440
Figueroa Gray, Marlaine S: 3065
Fike, Kayla J: 7350
Filippi, Melissa: 7325
Fillingim, Roger B: 1445
Finch, Caleb: 2040
Finchum, Tonya: 1385
Fine, Courtney: 1250
Finegan, Kerry: 4200
Fingerman, Karen: 4085, 4180, 7005, 7045, 7540, 7735
Fink, Howard: 4055, 7630
Finkel, Deborah: 4010,: 8015, 8015, 9015
Finlay, Jessica M: 3375, 4430, 7420
Finley, Erin: 8015, 8050
Finn, Laura A: 2535
Finnigan, Samuel: 4020
Finsel, Julia S.: 7050
Fiori, Katherine L: 4525, 7070
Fiorotto, Marta l: 1420
Fique, Morgan T: 8065
Fiscella, Andrew J: 7480
Fischer, Heidi: 8015
FIshbein, Kenneth W: 7210
Fisher, Gwenith: 4375
Fisher, Miranda: 3485
Fisher, Zachary: 7255
Fishman, Paul: 8010
Fisk, Calley: 1015, 3245
Fiske, Amy: 7000, 7220
Fitch, Catherine: 4035
Fitzgerald, Kathryn C: 7285
Fix, Rachel: 8015, 9025, 9040

Flanagan, Jane: 3230, 4235
Flatt, Jason: 2015, 2225, 3190, 3490, 4025, 7100
Fleet, Alexa: 7135, 7635, 7695, 7700
Fletcher, Jason: 4360
Fletcher, Jason M: 1280, 7660
Flood, Sarah: 4035
Florack, Janina: 1555
Florence, L. Carter: 1190
Flores, Carlos A: 1280
Flores, Efren: 8015
Flores, Emily V: 8000
Flores, Valerie: 4510
Flores-Cruz, Gabriela: 8020
Flores-Lagunes, Alfonso: 1280
Florez Acevedo, Stefani P: 1265, 7720
Floris, Patrizia: 7275
Floyd, Alex: 4195, 7110
Floyd, Alex C: 9040
Flynn, Sheryl: 8020
Fogleman, Connor: 4530
Foley, Olivia: 1130
Fonarow, Gregg: 9025
Fong, Arwen: 8015
Fonseca, Luciana LM: 4515
Fontaine, Leslie: 7660
Fontanese, Matthew: 7750
Foo, Jamie: 3115
Foradori, Megan A: 1490
Ford, Cassandra: 3585, 7700
Ford, James: 7705
Forrester, Sarah N: 3180
Fors, Stefan: 1450, 2060
Fortinsky, Richard H: 1275, 2495, 3210, 7740
Fosnight, Sue: 7440, 7475, 7900
Foster, Brandon: 3150
Foster, Justin: 8015
Foster, Norman L: 3175, 4070
Foster, Thomas C: 1445
Fountain, Brent: 4040
Fountain, William A: 7305
Fowler, Nicole: 4230, 8020
Fowler Davis, Sally: 3060
Fox, Aimee: 7480, 7900
Fox, Jennifer: 1035, 4310
Fox Ludden, Emily: 7465
Foxe, John: 8000
Foy, Capri G: 1315
Fracachan Cabrera, Monica: 8000
Fraiman, Paule Sarah: 8010
Francis, Sarah L: 7350
Francis- Edoziuno, Confidence: 8015
Franck, Maximilien: 7010
Frank, Robynn: 3345
Franzone, Monica S: 3515
Franzosa, Emily: 1395, 2430, 3105, 3485, 4480, 7045, 7420
Fraser, Renee: 7200
Fraser, Sarah: 1010, 2510
Frasier, Pamela: 8010
Fratiglioni, Laura: 3035
Frazier, Cleothia: 3235
Frazier, Robbin H: 2155
Frazier, Trivia: 1500
Freburger, Janet K: 7330
Freddolino, Paul P: 3415, 7745
Frederick, Jared: 7690
Fredolini, Claudia: 7010
Fredriksen-Goldsen, Karen I: 2420, 2540, 4495
Fredriksen-Goldsen, Karen: 8010
Freedman, Vicki: 3165, 7030, 8025
Freeland, Kerri S: 8015
Freeman, Shannon: 2360, 3205
Freer, Elinor: 9010
Freidus, Andrea: 8010
Freitag, Callie J: 3065, 4475, 7120
Freitas, Samuel: 7010
Frenkel Toledo, Silvi: 1245
Friberg-Felsted, Katarina: 3195, 3465, 4040
Fried, Terri R: 2305
Friedland, Hannah: 7460
Friedman, Ari B: 3550, 4220, 8015
Friedman, Ben: 7720
Friedmann, Erika: 3050, 8020
Friis, Marlene: 8020
Frimpong, Eric: 7240, 7405, 7635
Frissen, Laura: 4345
Fristedt, Sofi: 7155
Fritz, Julie: 9010
Froberg, Ruthann: 1145
Frogner, Bianca: 7545
Frohmberg, Eric J: 3100
Fromenthal, Ashley L: 1210
Fromknecht, Catharine Q: 7125
Fruhauf, Christine A: 3265, 3575
Fry, Carrie: 3075
Fry, Liam: 3240
Fry, Noelle: 7410, 7900
Fu, Jason: 7465, 8010
Fu, Liang: 8085
Fu, Lillian X: 3355
Fu, Longdi: 8010
Fu, Sunyang: 3135
Fu, WeiJie: 4535
Fu, Xiaoyu: 7235, 7610
Fu, Yu: 1195
Fudolig, Miguel: 4535
Fugate, Matthew (Brent): 4235
Fugate-Whitlock, Elizabeth: 2325, 3495, 8010
Fujihara, Satoko: 7490
Fujimoto, Kayo: 1105
Fujita, Koji: 7275, 7695
Fujiwara, Yoshinori: 7205, 7540, 7695
Fukuda, David H: 7205, 7640, 7735, 8015
Fukui, Sakiko: 7340, 7510
Fukushima, Kiyoko: 7405
Fukuzawa, Ai: 7360
Fulay, Aarohee: 8015
Fuller, Heather: 3405, 7025, 7070, 7090, 7605, 7745
Fuller, Patrice M: 3140
Fuller, Shannon: 2520
Fuller-Thomson, Elysia G: 7025
Fuller-Thomson, Esme: 7025
Fulmer, Terry: 4355
Fulzele, Sadanand: 7210, 8015
Fung, Chi Tat: 4195, 8015
Fung, Constance: 8015
Fung, Helene H.: 1375, 3480, 4020, 7355, 7610, 8000
Fung, Helene Hoi-Lam: 9035
Fung, Nicole L: 1375
Funk, Laura: 2495
Furht, Borko: 7360, 7610
Furman, David: 2530
Furstenberg, Frank: 7350
Furuya, Alex: 4550
Fusca, Laura A: 8010
Futterman, Andrew: 7150

G

Gaballah, Nada A: 1155
Gaber, Sophie N: 1555
Gabralla, Sarra: 8015
Gabriel, Kelley Pettee: 7720
Gabrio, Andrea: 4345
Gad, Dustin: 8020
Gadbois, Emily: 4460
Gaeta, Laura: 1050
Gaglio, Marisa: 8000
Gagne, Danielle: 3495
Gagnon, Christine: 4490
Gahche, Jaime: 8015
Gaillard, Trudy: 2330
Gaines, Bryan F: 1060, 4010
Galambos, Colleen: 2555
Galanza, William Son: 7155
Galasko, Douglas: 7005, 8015
Galatioto, Jessica: 7140
Galik, Elizabeth: 4090, 8020
Gallagher, Michael: 7040
Gallagher-Thompson, Dolores L: 1135, 8040
Gallant, Kayla O: 7105
Gallistl, Vera: 2565, 3315, 4275
Gallo, Joseph: 3570, 7615, 8000
Galloway, Rebecca: 3340
Galucia, Natalie: 3310, 8010
Galvin, James: 4430, 8015
Gamache, Seana: 2130
Gamaldo, Alyssa A: 1250, 2055, 2330, 7735, 8000, 8020
Gamaldo, Charlene: 7735
Gamble, Julia: 2145
Gamper, Mary Jo: 2085
Gan, Daniel R Y: 3200, 4320, 8010, 8020
Ganbat, Uyanga: 7205
Ganci, Aaron: 2410
Gandy, Stacey: 7250
Gandy, Tyler: 2360
Gangnon, Ronald: 7160
Gangula, Sushma: 7540
Gangupantula, Pratima: 2535, 7530
Gans, Hannah: 1010
Gao, Ashley: 8020
Gao, Bruce: 8000
Gao, Courtney: 8085
Gao, Fengli: 7470
Gao, Haihua: 7670
Gao, Hongxin: 2465
Gao, Oude: 4550
Gao, Shangde: 1445
Gao, Sibo: 7045, 7540
Gao, Siyu: 2150, 3270
Gao, Sujuan: 4440
Gao, Wei: 7030
Gao, Xianqi: 7355, 8015
Gaona, Carlos: 7735
Garbarino, Valentina R: 3020
Garber, Johnathon: 7300
Garberson, Lisa A: 2500
Garbin, Alexander J: 2135, 4215

García, Catherine: 1320, 3235, 4015, 8020
García De La Garza, Ángel: 2160
Garces Torres, Ana Sophia: 7065, 8020
Garcia, Alexandra: 8010
Garcia, Andrea: 8025
Garcia, Ivis: 1295
Garcia, Jose M: 7305
Garcia, Lizbeth: 7005
Garcia, Marc A: 2120
Garcia, Mary Ellen: 8010, 8015
Garcia, Michael A: 1540
Garcia, Reagan E: 1240
Garcia, Thelma: 4170
Garcia Marquez, Grecia Gabriella: 1420
Garcia Marquez, Grecia G: 8000
Garcia Morales, Emmanuel E: 1055, 1440
Garcia-Aymerich, Judith: 2170
Garcia-Chanes, Rosa: 3030
Garcia-Davis, Sandra: 2545, 8010, 8070
Gardener, Hannah: 8085
Gareri, Michele: 7440, 7475, 7900
Gargiulo, Paolo: 2240, 7305
Garner, Charity: 8020
Garner, Kayla M: 7455
Garnett, Anna: 4270
Garrison, Brianna V: 2260
Garrity, Katherine: 7140
Garrow, George: 7410
Gary-Webb, Tiffany L: 3375
Gaskin, Darrell: 3030
Gassoumis, Zachary D: 2001, 2150, 2375, 7405
Gaster, Barak: 1430
Gaston, Harriett: 7690
Gately, Megan: 3395
Gatenio-Hefling, Orly: 1435
Gatz, Margaret: 4375
Gaugler, Joseph E: 1260, 2015, 2155, 2350, 3025, 3130, 3425, 3450, 4235, 7640, 7685, 8020
Gauss, C. Heath: 7360, 7630
Gautam, Ramraj: 1050, 2440
Gawryluk, Jodie R: 7525
Gay, Emma: 1240
Gayda, Mathieu: 4490
Gaye, Angela: 7030
Ge, Huini: 7510
Ge, Mei-Ling: 8015
Ge, Shaoqing: 2190
Gea-Sánchez, Montserrat: 4255
Gee, Nancy R: 3050, 8020
Gehling, Jacklyn A: 1020
Geissal, Erik D: 7350
Geisser, Sophia R: 1070, 1210, 3240
Geldhof, John: 7730
Gelfond, Jonathan: 4310
Gelinas, isabelle: 3185
Gell, Nancy: 2450, 7655
Gelmon, Sherril B: 4235
Gendron, Tracey: 1010, 8000
George, Claudene: 8000
George, Daniel: 3380
George, Kiran: 7000
George, Mollie J: 8000
George, Regina A: 3240, 7065, 7685
George, Rickey E: 8015
Gerald, Melissa: 3420
Gergis, Mary: 7475, 7900
Gerken, Danielle R: 8010
Germain, Cassandra M: 8000
Germine, Laura T: 4515
Gerstorf, Denis: 4065, 4500, 4525, 7030, 7210, 7535, 8020
Gertin, Robin: 7055
Gettel, Cameron: 1130, 8010
Getz, Marci J: 3100
Gharib, Sina A: 7305
Ghazi Saidi, Ladan: 2205, 7465
Ghezzi, Elisa: 3305
Ghiasi, Akbar: 1425, 1550
Ghimire, Saruna: 4015
Ghoraani, Behnaz: 7610
Ghose, Sarah M: 8010
Ghose, Urmimala: 3040, 4525
Ghosh, Bhaswati: 4170, 8000
Ghosh, Neeta: 7530, 7905
Ghosh, Ritam: 7155
Giannotto, Emily L: 7225
Giardino, Elizabeth: 7420
Giasson, Hannah L: 3080
Gibbon, Samuel: 7425, 7930
Gibbs, Lisa: 2340, 7405
Gibson, Allison: 3370, 7545, 7905, 8020
Gibson, Regina v: 8015
Giffuni, Jamie: 7080, 7315, 7720, 8015
Gigi, Amateau: 1010
Gil, Efrat: 1435, 7300
Gil, Heidi: 1345, 4165
Gilani, Owais: 2045
Gilbert, Lauren R: 3485
Gilchrist, Laura: 7000
Gilfix, Talia: 7355
Gill, Amarjot S: 7435, 7915
Gill, Jessica: 8000
Gill, Thomas: 2020, 3340
Gilleard, Chris: 3015
Gillespie-Heyman, Sarah: 7475
Gilligan, Megan: 3055, 3165, 7005, 7265
Gilliland, Jaime: 2295
Gilmore-Bykovskyi, Andrea L: 3425, 4530, 7200, 8010
Gilsanz, Paola: 7535
Gil-Silva, Mauricio: 8000
Gimm, Gilbert: 2415, 7270
Gingerelli, Toni: 3570
Ginggeaw, Sangduan: 4465
Ginn, Brandi: 3175, 4450
Ginoux, Laura: 7635
Ginsberg, Charles: 3330
Giordani, Bruno: 8000
Giordano, Gregory: 3115
Giordano, Stephanie J: 4245
Giorgio, Luciana: 8000
Giraldo Santiago, Natalia: 1345, 2315
Girgus, Joan: 7235
Girling, Laura: 2015
Gislason, Magnus Kjartan: 2240, 7305
Gitelman, Darren R: 7425, 7930, 8070
Gitlin, Laura N: 1260, 3395, 4130, 4340, 7685, 8020, 9010
Gjestsen, Martha: 8015
Glaser, Karen: 1475
Glass, Lauren: 1520
Gleason, Carey E: 2190, 2335
Gleason, Kelly T: 2085
Gleason, Lauren: 2340
Glennie, Kristen S: 1005, 7075
Glickman, Adam: 4290
Glicksman, Allen: 3550
Gliebus, Gediminas: 9010
Glockner, Katherine: 4310
Gloger, Elana M: 9010
Glover, Crystal M: 4025
Glymour, M Maria: 1545, 7535, 9010
Glynn, Jennifer: 8015
Glynn, Nancy W: 1240, 1410, 2035, 7060
Godin, Judith: 8000
Goel, Sanchit E: 4180
Goerzen, Maya: 3465
Gogia, Rhea: 7530
Goldberg, Allon: 7300
Golden, Blair P: 3335, 4530
Goldfeld, Keith: 3010
Golding, CJ: 7250
Goldman, Alyssa: 1140, 3405
Goldman, Dana P: 1390
Goldstein, Karen: 4530
Goldtooth, Amanda: 1525, 8000, 8020
Goler, Timothy: 1140
Golithadka, Kaushik: 1060, 4010
Gomes Goncalves, Natalia: 1320
Göbl, Linda: 7680
Gomez, Melissa: 8010
Gomez, Morgan: 8000
Gomez, Rowena G: 2440, 8000
Gomez, Scarlett Lin: 2020
Gómez Cáceres, Bárbara: 8000
Gonçalves, Rui: 8010, 8015
Gondo, Yasuyuki: 1385, 2470, 7115, 7750, 8020
Gong, Jessica: 4440, 7005
Gong, Ke: 8000
Gong, Ni: 7080
Gong, Zengliang: 1230
Gongloor, Pratik: 7400, 7920, 8015
Gonyea, Judith G: 7215
González, Hector: 7455
Gonzales, Ernest: 2335, 2470, 8000, 8020
Gonzales, Gilbert: 3075
Gonzales, Mitzi: 4210, 7500, 7735, 7930
Gonzales, Samantha: 7540, 7735
Gonzalez, Amanda: 8010
Gonzalez, Anais Claire: 7210
Gonzalez, Andrea: 7000
Gonzalez, Hector: 7540
Gonzalez, Richard: 3540, 8025
Good, Mary K: 1395
Goodman, Claire: 4255
Goodman, Jeri: 4360
Goodman, Octavia K: 9025
Goodpaster, Bret: 1240, 4310
Goodrich, Grant: 2355
Goodwin, Laurance: 7440
Goodwin, Richard: 8000
Gooneratne, Nalaka: 7525

Gopalakrishnan, Nisha P: 8010
Gorbenko, Ksenia: 8010, 8010
Gorbunova, Galina: 7200
Gordon, Barbara: 7340, 7730
Gordon, Dawn L: 3100
Gordon, Emma: 1025
Gordon, Kate: 3565
Gordon, Kristina C: 8020
Gordon, Stacey: 2470
Gordon, Taryn: 3140
Gordon-Larsen, Penny: 7275
Gore, Janelle: 4175, 4390
Gore, John C: 7010
Gore, Shweta: 7720, 8020
Gorelik, Aaron: 8010
Gorla, Vandita: 8000
Goronzy, Jorg: 3160
Gorospe, Myriam: 7305
Gorzelle, Gregg: 7465
Gorzycki, Emily: 1265
Goss, Arianna P: 7200
Gossner, Jacob D: 4435, 7065, 7270, 8020
Gotch, Chad M: 7350
Gothard, Sarah: 1125, 3140, 7425
Gothe, Neha: 7545
Goto, Jun: 7420
Gou, Yang: 7315
Gould, Christine E: 1135, 2280, 3110, 7135, 7235, 7480, 7900
Gourley, Amy N: 7605
Gourley, Keone: 3205
Gouveia, Gillian: 8000
Goyal, Riya: 7425, 7905
Gozalo, Pedro L: 4460, 7200, 7295
Gozalo, Pedro: 8010
Grünjes, Carlotta E: 7535
Grabanski, Morgan: 8000
Grabowski, David C.: 8020, 8055
Grace, Rachel: 7360
Grady, Alexandra: 7100
Grady, James: 2495
Graf, Allyson S: 3450, 7345
Graf, Peter: 8000
Graff, Julie N: 7020, 7205
Graham, Cameron: 7335
Graham, Carrie L: 4510
Graham, David: 7260
Graham, Eileen K: 1570, 4375, 4480, 7455
Graham, Karen L: 2215
Graham, Laura A: 8010
Graham, Wan Chen K: 3545
Graham-Engeland, Jennifer E: 4405, 4515, 7210, 7455, 8020
Gramani-Say, Karina: 7225, 7655
Granbom, Marianne: 1285
Grande, Giulia: 4315, 7010
Grandy, Scott: 8000
Granger, Teresa Ann: 8015
Grant, Claire: 4200, 8020
Grant, Elizabeth: 8015
Grant, Jeremy G: 1495
Grant, Nicole: 4300
Gras, Laura Z: 7330
Graupner, Jeffrey: 2340, 8020
Gravenstein, Stefan: 7030, 8010
Gray, Jullie: 2495
Gray, Shelly: 7320
Green, Ariel R: 2085
Green, Bev: 1265, 9020
Green, David M.: 7205
Green, Dr. Robin R: 1365
Green, Gavin: 8020
Green, Krystal: 7350
Green, Makenna R: 8010
Green, Michael: 7625
Green, Natalie: 2470
Greenberg, Barry: 3070
Greenberg, Jeremiah J: 8015
Greenberg, Jonathan: 2315
Greenberg, Phyllis A: 7250
Greene, Barry: 7635
Greene, Celeste M: 8070
Greenfield, Annabelle: 1165
Greenfield, Emily A: 3340
Greenfield, Jennifer C: 2480
Greenwald, Blaine: 7695
Greenwood-Hickman, Mikael Anne: 1265, 7720, 8010, 9020
Gregg, Jeffrey J: 2280
Gregor, Neil: 7080
Gregorio, Mario: 7465, 8010
Gregory, Sarah: 7425, 7930
Greig, Erin E: 2510
Greiner, Chieko: 8015
Grenier, Amanda: 4275
Grewal, Kiren: 7200
Griffin, Hannah: 8010
Griffin, Joan M: 1475, 4415
Griffin, Rebekah: 7750
Griffith, Candace: 7030
Griffith, Christopher X: 7695, 8020
Griffith, Kathleen: 8000
Griffith, Lauren E: 7030
Griffiths, Leroy: 8015
Griffiths, Patricia C: 8020
Griggs, Stephanie: 2015, 7270
Grigorovich, Alisa: 3315, 4445
Grilli, Matthew: 8020
Grimes, Darla: 7145
Grimm, Kevin: 4065, 4500, 8040
Griner, Stacey: 7700
Grineski, Sara E: 7420, 7455
Grisales, Maria: 7425, 7630
Griswold, Michael E: 1000
Grittner, Alison L: 2395, 2560
Groden, Sheryl: 7155, 7740
Grodsky, Eric: 1095, 7230, 7350
Grol-Prokopczyk, Hanna: 7225
Gronlund, Carina: 2040
Grooms, Jevay: 8000
Groover, Kimberly: 1015
Gross, Alden: 1290, 2515, 3070, 3335, 4380, 4440, 7015, 7225
Grossberg, George: 8020
Groulx, Mark: 3205
Groves, Angela: 1465, 2330
Growney, Claire M: 1485, 1575
Grubbs, Allison: 1130
Grubbs, Brandon: 1265, 7075
Gruber-Baldini, Ann: 2015, 3495, 8010, 8095
Grudzen, Corita: 3010
Grudzien, Adrienne: 7540, 7735
Gruenewald, Tara L: 4375, 4505
Gu, Danan: 7155
Gu, Yian: 1025, 1580
Guan, Ting: 7020
Guan, Weihua: 7010
Gubner, Jennie M: 4480
Gubrium, Aline: 2555
Gudnason, Vilmundur: 2040
Gudzune, Kimberly: 7710
Guerbaai, Raphaelle-Ashley: 4255
Guerra, Gabriella: 2565
Guest, M. Aaron: 2245, 2455, 3080, 3560, 4040, 4320, 8000, 9000
Guevara, Nathalie: 2520
Gugliucci, Marilyn R: 2490, 3195, 8010
Guha, Deyaan: 8000
Guidarelli, Carolyn: 7205
Guidry, John A: 7110
Guilcher, Sara: 8015
Gujjala, Vikas A: 7005
Gumikiriza, Joy Louise: 1190
Gunsalus, Paul: 7235
Gunzler, Douglas: 7235, 7690, 8000
Guo, Crystal: 4340
Guo, Guifang: 1355
Guo, Jia-Wen: 4520
Guo, Man: 4110, 7235, 7700, 8050
Guo, Niting: 4065
Guo, Xiufang: 7425
Guo, Yingqi: 3200
Guo, Yu: 3270
Guo, Yuxiang: 8015
Gupta, Aashish: 9035
Gupta, Avinash: 8010, 8020
Gupta, Ayush: 8015
Gupta, Ravi: 7470
Gupta, Shivani: 1425, 1550, 7510
Gupta, Sonu Kumar: 7210
Gurchiek, Reed D: 8015
Gurlu, Merve: 3050, 8015, 8020
Gurung, Shreemouna: 2560
Gurupur, Varadraj: 8015
Gurven, Michael: 7010
Gurvich, Olga: 1265
Gur-Yaish, Nurit: 4075, 7265, 7400, 7920
Gusmano, Michael K: 1360
Gustafson, Deborah: 8010
Gustafson, Sally: 7455
Gustat, Jeanette: 8020
Gustave-Schelling, Kyla: 7075
Gustavson, Allison M: 3130, 4415
Gustavson, Daniel: 8020
Gutierrez, Angela: 1045, 1495, 3475, 4535, 7535, 7540
Gutierrez, Eduardo: 7000
Gutierrez, Jose: 8085
Gutierrez, Mariela: 1170
Gutierrez, Sirena: 7235, 9000
Gutierrez, Victoria: 8010
Gutierrez-Ramirez, Paulina: 4130, 7270, 7660
Gutman, Gloria M: 7155, 7405
Gutman, Roee: 3240
Gwathmey, TanYa M: 1315

### H

Ha, Jung-Hwa: 1375, 4070
Haas, Jennifer: 8015
Haase, Claudia: 3355

Haber, Abigail: 8000
Håberg, Asta K: 2425, 7060
Haberstroh, Julia: 1555
Hacker, J David: 4035
Hacker, Sarah D: 7080
Hackett, Katherine: 3250, 7630
Haddad, Yara: 1235
Hadidi, Niloufar N: 1265
Hadlandsmyth, Katherine: 7205
Hadley, Lesca: 1065
Hadsell, Killian: 7680
Hadson, Kim: 8010, 8045
Hafner, Camryn: 2175, 7480
Hagaman, Ashley: 8010
Hagemann, Lauren: 4060, 4330
Hagen-Lillevik, Synneva: 2060
Hägg, Sara: 7010
Haggar, Katherine: 3190
Haglund, Kristin: 2555
Hahm, Bridget: 2145
Hahne, Jessica: 7055
Hahn-Goldberg, Shoshana: 8010
Haigh, Sylvia: 7250
Haim, Todd: 1500
Haines, Terry P: 2145, 4100
Hair, Nicole: 1280
Haire, Cameron: 7205
Haitsuka, Kilohana: 8010
Haji Hosseini, Sogol: 2395, 4320, 7155
Hajjar, Emily R: 7020
Hakanson, Megan: 1520
Hakian, Mia: 8000
Halasz, Ildiko: 3335
Halaszynski, Jaime: 8015
Haldar, Srabani: 3165, 7065
Halder, Puja: 3055
Hale, Jo: 3530
Haley, William: 2370, 3495, 7065, 7070, 7140
Halilaj, Eni: 8000
Hall, Charles B: 4185
Hall, Katherine S: 2345, 7080, 7155
Hall, LaToya N: 2155
Hall, Lauren: 2430, 3485, 7045
Hall, Leighanna N: 7000
Hall, Lisa C: 7480
Halloway, Shannon: 7615
Halpern, Scott D: 8015
Halpin, Sean N: 2445
Halvorsen, Cal: 2425, 3310, 4140, 4320, 7355, 8020
Ham, Carolyn R: 4510
Hamada, Fumiko: 8020
Hamann, Kate: 1465
Hamel, Kate: 1050
Hameleers, Niels: 7295
Hamer, Mark: 1245
Hamers, Jan PH: 2140, 4255, 7295, 7685, 8015
Hamill, Randi: 8010
Hamilton, Jill B: 8020
Hamilton, Karyn L: 7075
Hamilton, Lucas J: 4295, 9015
Hamler, Tyrone: 7700, 7935
Hamlin, Abbey M: 9000
Hamm, Jeremy M: 1525, 7030, 7455, 8020
Hammad, Suzanne: 1155
Hammers, Dustin: 8000
Hammill, Bradley G: 7625
Hampton, Marsha: 2055, 8020
Hamrick, Mark: 7285
Han, Chengming: 1170, 7140, 7420
Han, Duke: 1370, 2350, 3220, 7495, 7930
Han, Gang: 4130, 4340
Han, Hae-Ra: 1440
Han, Jayoung: 8015
Han, Jimin: 7275, 8015
Han, Ling: 7060
Han, Sae Hwang: 1405, 4400, 7690, 8020
Han, Shuo: 3470
Han, Soojeong: 8015, 8040
Han, Sunghei: 8010
Han, Xianlin: 4170
Han, Yang: 7030
Hanaoka, Hideaki: 7270
Hancock, Cynthia: 8010
Hancock, David W: 7205, 7405
Hancock, Jeffrey T: 8020
Hancock, Kevin: 2480
Hancock, Laura M: 4300
Hand, Brittany: 4475
Hand, Michelle D: 2370, 4540
Handa, Satoru: 1505
Handique, Swasati: 7005, 8010
Handra, Amy: 7265
Handy, Todd: 7720
Hanes, Douglas W: 3015, 4450, 7100
Hanna, Karim: 1355
Hannan, Marian T: 2080
Hansen, Abigail: 3185
Hansen, Jared: 2545
Hansen, Maria Swennergren: 4075
Hansmann, Kellia J: 7160
Hanson, Angela: 8000
Hanson, Emily J: 8000
Hanson, Laura: 8015
Hanson, Leah R: 7455
Hao, Qiukui: 7655, 7720
Hao, Ying: 8000
Happ, Mary Beth: 2415
Happel, Christine: 2070, 3080
Haque, Sabrina: 7155
Harada, Ann SM: 1390
Harada, Ko: 7260
Harding, Rachel: 8010
Hardy-Smith, Oliver: 2330
Harkins, Kristin: 8015
Harrell, Erin R: 1045, 7750
Harrington, Charlene: 8015
Harrington, Christina: 3315
Harrington, Erin E: 4280, 4515, 7200, 7455, 8020
Harrington, Laura B: 8010
Harris, Christina: 7350
Harris, Daniel: 7030
Harris, Jenny: 2465
Harris, John: 7510, 8010
Harris, Katherine: 9030
Harris, Maude: 7440, 7935
Harris, Rebekah: 3335
Harris, Robin: 7720
Harris, Steven: 1020
Harrison, Sean Lee: 7145
Harrison, Tracie C: 7700
Harris-Speight, Jasmine: 3575
Hart, Dani: 2555
Hart, Julia: 8010
Hart, Laura A: 1435
Hart, Sara E: 3465, 7065
Hartley, Gregory W: 7330
Hartmann, Christine W: 1070, 3240, 7110, 7250, 7420, 7685
Hartnell, Lisa M: 8000
Hartung, Johanna: 4185
Haruo, Emy: 1470
Harvey, Idethia Shevon S: 7440, 7935
Harvey, Kelsey: 3315
Harvey, Shanelle: 7055
Hasche, Leslie: 4115, 7650, 7720
Hasegawa, Nanako: 8015
Hash, Kristina M: 8000
Hashizume, Yumi: 7125
Haskins, Anne: 7010
Haslam, Lynn: 9040
Hasnani, Madison: 7440
Hass, Zachary: 2245
Hassell, Susan J: 4450, 7545
Hassenstab, Jason: 4515
Hasten, Lauren N: 7545, 7905
Hastings, Susan N: 1130, 2145, 2180, 7685
Hasworth, Serena: 3560
Hata, Hiroshi: 8015
Hata, Toshiki: 7275
Hatami, Fatemeh: 3455
Hatanaka, Sho: 8015
Hatch, Maddy: 7110
Hathaway, Wendy: 7730
Hatten, Christina: 7695
Haunch, Kirsty J: 4255, 7295, 7520
Hauptschein, Max: 2310
Hausdorff, Jeffrey: 1235
Haverhals, Leah M: 3485, 8015, 9025, 9040
Hawes, Frances: 3450
Hawks, Cate: 3565
Hawks, Zoe W: 4515
Hawley, Chelsea: 2345
Hawthorne, Timothy L: 2300, 7025
Hayajneh, Audai A: 7435
Hayashida, Cullen T: 7145
Hayat, Yumna: 7465, 7910
Hayden, Kathleen M: 1390
Hayek, Roee: 1245, 2240
Hayes, Kaley N: 7030
Hayes-Larson, Eleanor: 1545, 7535
Haynes, Laura: 1580
Hayslip, Bert: 7265
Hayslip, Natalie: 8000
Hayward, Mark D.: 3370
Hayward, Matt: 8085
Hazelett, Susan: 7440
Hazlewood, Hudson: 7470
Hazuda, Helen: 8015, 8050
He, Chanchan: 7630
He, Daqing: 1355, 2355
He, Haiyan: 2255, 2500, 4055
He, Jasmin: 2150, 4365
He, Leo: 3415
He, Lingxiao: 1365, 2500, 7305
He, Ranran: 7070
He, Rendong: 7695, 8015
He, Xiaona: 7030
He, Xin: 7060
He, Yao: 4070
He, Zhe: 1355
He, Ziyi: 3480
Head, Denise: 7720
Heath, Sarah: 7720
Heaton, Felicia: 2140
Hebblethwaite, Shannon: 3320
Hebert, James: 2080, 7315

Hebpurn, Kenneth: 2295
Hecht, Crystal A: 7605
Heckhausen, Jutta: 4525
Hedayati, Niloofar: 4320
Heffner, Kathi L: 2315, 3045, 9010
Heidenreich, Paul: 9025
Heimler, Stephanie R: 4170
Hein, Maria: 7650
Heinz, Melinda: 1385, 7265, 7350
Helbostad, Jorunn L: 2170
Heldin, Paraskevi: 8000
Heldreth, Courtney: 3315
Helgertz, Jonas: 4035
Helmly, Victoria: 3420
Helsabeck, Nathan P: 7490, 7935
Hemmy, Laura: 1485, 7145
Hemrajani, Girish: 8010
Henderson, Alexandria: 9030
Henderson, Charles: 4060
Henderson, Wendy: 8015
Hendricksen, Meghan: 7420
Hendricks-Ferguson, Verna: 4080, 7520
Hendrix, Cristina: 4450, 7270
Hendry, Anne: 7215
Hengerer, David: 1370
Heniff, Erin M: 8035
Henion, Amy: 7630
Henke, Maria LG: 1005, 2250
Henneman, Lucas: 4035
Henning-Smith, Carrie: 4105
Henricks, Evan: 2535
Henry, Kyle: 7155
Henry, Maureen: 1200
Hensley, Robert B: 7115
Hentges, Rochelle: 4085
Hentnik, Haley B: 8015
Heo, Jinmoo: 3050, 4505, 7070, 7280, 8080
Heo, Seok-Jae: 8015
Hepburn, Kenneth: 1125, 2295, 3045, 4165
Hepple, Russell T: 2080
Herd, Pamela: 1280, 3225
Herman, Hattie: 2570
Hermann, Carla P: 4335
Hermanns, Melinda: 4240
Hernández, Belinda: 8060
Hernández de Jesús, Alma: 3175, 4450, 7325
Hernández Gamboa, Rebeca: 1535
Hernandez, Beatriz: 7715
Hernandez, Bianca: 7425, 7905
Hernandez, Christopher: 4520, 8020
Hernandez, Elise: 3490
Hernandez, Elizabeth: 7410, 7900
Hernandez, Ester: 8000
Hernandez, Fernando: 7470
Hernandez, Frank: 7005
Hernandez, Herb Howard C: 8015
Hernandez, Jessica A: 1510
Hernandez, Jessica: 1510
Hernandez, Raymond: 2465
Hernandez, Rolando: 7210
Hernandez Arciga, Ulalume: 8000
Hernandez Cervantes, Laura E: 7010
Hernandez-Ochoa, Erick: 7305
Hernandez-Tejada, Melba A: 2520
Herpich, Catrin: 7315
Herpst, Jessica L: 7005, 7465
Herreid-O'Neill, Anders: 8010
Herrick, Tamara H: 3565
Herrig, Shelby: 3215
Herrmann, Francois R: 1220
Hertel, Johannes: 7010
Hertzog, Christopher: 7225
Her-Xiong, Youhung: 4445, 7600
Herzog, Monica Anderson: 4060
Heselton, Hyeon Jung (Judith): 8020
Hesgrove, Brandon W: 7545
Heston-Mullins, Jennifer: 7435
Hetherington-Rauth, Megan: 7060
Heuer, Sierra: 8025
Hewitt, Elizabeth: 8010
Hews, Ellis J: 8020
Hewson, Jennifer: 3465
Heyn, Patricia C: 1300, 2455, 3460, 3505
Heys, Jodi L: 7065
Hiatt, Shirin O: 7440
Hicken, Bret L: 2430
Hicken, Margaret: 7350
Hickman, James: 7425, 7630
Hickman, Ronald L: 2015
Hickman, Susan: 4440, 8010, 9040
Hicks Patrick, Julie: 8000
Higbie, Anna: 8020
Highberg, Nels P: 2175
Hilderbrand, Zoey: 8070
Hilgeman, Michelle M: 1070, 1080, 1210, 3240, 7250, 7685
Hill, Carl V.: 3180, 3475
Hill, Cristal: 8000
Hill, Heather: 7120, 8010
Hill, Jordan R: 7005
Hill, Julianna: 7480, 7900
Hill, Kyrsten C: 4405
Hill, Kyrsten: 8000
Hill, Linda: 7080
Hill, Nikki: 1525, 4280, 7630
Hill, Patrick L: 8000, 8020, 8080
Hill-Ashford, Yolanda: 7030
Hilmer, Sarah N: 3520
Hilsabeck, Robin C: 1355, 2355
Hiltz, Sydney: 7720
Himes, Dani: 2185, 8010, 8010, 8020
Himes, McKenzie: 7200
Himmelfarb, Cheryl Dennison: 7440, 7500
Himmelsbach, Ines: 7480
Hinderberger, Brian: 3285
Hinrichs, Raegan: 7000
Hinrichs-Kinney, Lauren: 2135
Hinson, Justin: 3190
Hinton, Allyshia V: 8015
Hinyard, Leslie: 4080
Hirano, Hirohiko: 7205, 7540, 8015
Hirdes, John P.: 7620
Hirsch, Ben: 3485
Hirsch, Bruce: 8010
Hirsch, Jana A: 3375, 8020
Hirsch, Jen: 3370, 8010
Hirschman, Karen: 3300, 3385, 7435, 7535, 8010
Hirsch-Matsioulas, Orit: 3465, 4040
Hisamatstu, Yasushi: 7400, 7920
Hites, Kenneth W: 2175
Hiyama, Atsushi: 8015
Hladek, Melissa deCardi: 2010, 3150, 4420, 8000
Hladyshau, Siarhei: 8000
Ho, Alvin K K: 4390
Ho, Anita: 2565, 4435
Ho, Cheuk Yin: 4195
Ho, Chin Wei: 7155
Ho, Dorian: 2180
Ho, Rainbow Tin Hung: 2520, 7725
Ho, Simon: 8010
Ho, Tuong-Vi: 2205
Ho, Yuen Wan: 7725, 8020
Hoban, Luke: 7290
Hoch, Jackson: 8010
Hochberg, Marc: 7010, 7440
Hochman, Yael: 3465, 4040
Hodes, Richard J: 1340, 2210
Hodgins, Maddison L: 8000
Hodgson, Nancy: 3215, 4200, 7525, 8015, 8020
Hoel, Sydney: 4530, 7005, 7270, 8020
Hoeppner, Bettina B: 2315, 3250
Hof, Patrick R: 8000
Hoffarth, Phillip: 8020
Hoffman, Adar: 1220
Hoffman, Elise V: 2450
Hoffman, Geoffrey: 7030
Hoffman, Jessica: 8000
Hoffman, Rashelle: 2360, 3285, 8060
Hoffman, Yaakov S: 1565
Hofstra, Emma: 8010
Hogan, David B: 3465, 7030
Hohman, Timothy J: 7015
Holaday, Louisa: 7535
Ho-Lastimosa, Ilima: 8020
Holben, Rachel: 7695
Holcombe, Sven A: 1435
Holden, Erika: 9020
Holden, Richard J: 2410
Holland, Alexandra: 7025
Holland, Carol A: 3060
Holland, Diane: 1475, 4415
Holland, John: 3100
Holland, Sydney N: 8000
Hollandsworth Reed, Jenn: 4235
Holley, Lyn M: 1310, 2175, 3345, 3585, 8010
Hollingshaus, Mike: 4520
Hollingsworth, Holly: 7260
Hollis, Linda: 1050
Hollman, Nicholas: 7330
Hollmann, Peter: 7200
Holloway, Jeremy C: 2130
Holm, Johanna B: 7210
Holman, E. Alison: 7030
Holmes, Holly M: 3135
Holmes, Rachel: 7210, 7715
Holmes, Sarah D: 1070, 2000, 3255, 8020
Holt, Nigel: 8015
Holteng, Lise Birgitte: 8015
Holtzer, Roee: 1150
Homann, Jan: 7030, 7210

Homayouni, Ramin: 8010, 9050
Hommel, Erin: 7400, 7920, 8015
Honda, Ayumi: 7030
Honda, Sumihisa: 7030
Hong, Chenlu: 4065
Hong, Dahye: 7080, 7275, 7630 8015
Hong, Dongfang: 7350, 7735
Hong, Keung-Ja: 7460
Hong, Michin: 3545, 7425, 7630, 7935
Hong, Philip: 7545
Hong, Sungjae: 4420, 8020, 9050
Hong, Yinglu: 4535
Honig, Lawrence S: 1025
Honore, Chelsea: 7425, 7630
Hood, Michelle M: 1365
Hoodin, Derek: 1015
Hoogendijk, Emiel O: 2230
Hoover, Julia: 7210
Hooyman, Andrew: 7325
Hope, Peter: 4300, 7150
Hopfer, Elizabeth: 8010
Hopkins, Lauren: 7735
Hopp, Faith Pratt: 8010
Hopper, Shawna: 7720
Hoppmann, Christiane: 3480, 7535, 7725, 8000, 8020
Horbal, Steven R: 1435
Horhota, Michelle: 1010
Horiuchi, Takashi: 8015
Horn, Amanda J: 3170
Horn Mallers, Melanie: 7475
Horne, Kensley M: 4370
Horowitz, Chloe S: 1525
Horstman, Molly: 4340, 4415, 7680
Hort, Jakub: 8000
Horwitz, Shelley: 3265
Hoselton, Jill H: 2560
Hosking, Diane: 3185
Hossain, MD Sarafat: 7055
Hossain, Mohammad S: 7065, 7215
Hossain, Mohammad D: 8075
Hossain, Sayeem: 7000
Hotz, V. J: 4050
Hou, Erin: 7110
Hou, Su-I: 2100, 7460, 7925, 8010, 8015
Hou, Zhaoyi: 7425, 7930
Hougaard, Benjamin T: 8000
Houge, Angela: 7410
Hough, Katrina A: 2520
Houlihan, Daniel: 7510
Houmard, Joseph: 1410
Houston, Denise K: 1390, 1410, 2505, 7710, 8015
Hoverman, Adam: 8020
Hovland, Cynthia A: 7040, 7475, 7900
Howard, Annie Green: 7275
Howard, Brittni: 8010
Howe, Clarissa: 7455
Howe, Judith L: 2280, 2345, 4150
Howe, Matthew: 7040
Howe, Melissa J K: 8010
Howe, Rebecca: 8000, 8010
Howell, Britteny M: 7480
Howell, Cynthia: 3170
Howington, Marshall: 4005
Howlett, Susan E: 3520, 8000
Hoy-Ellis, Charles P: 2420, 4495
Hoyt, Margaret: 8010
Hren, Katie: 7350
Hristidis, Vagelis: 4060
Hsieh, Jenny Yi-Chen: 7625
Hsieh, Jessica: 4365, 7480, 7900
Hsieh, Katherine K: 8015
Hsiung, Ging-Yuek R: 3335, 8015
Hsu, Chun-Liang: 1150, 7720
Hsu, Clarissa: 3010, 4230
Hsu, Erh-Chi: 8010, 8015
Hsu, Fang-Chi: 2505, 7710, 8000
Hsu, Hui-chuan: 7735
Hsu, Jean W: 2235
Hsu, Joanne: 7050
HSU, Sheng-Chin: 8010
Hsu, Wan-Chen: 7540
Hsu, Wanhsiang: 3255
Hsu, Wan-Ling: 7465
Hu, Elise: 8020
Hu, Grace: 7750
Hu, Jing: 8010
Hu, Jinxiang: 7635
Hu, Luxin: 1190, 7265
Hu, Mengyao: 8025
Hu, Mingyue: 1245
Hu, Qijia: 7005
Hu, Rita X: 2305, 2475, 3040, 3400
Hu, Susan C.: 7540
Hu, Ting: 7120
Hu, Xiuying: 1355
Hu, Yi-Ling: 8015
Hu, Yu: 1435
Hu, Yuanyuan: 7145
Hu, Yu-Ching: 7525
Hu, Yue: 3480
Hu, Yuexia: 1090
Hu, Zhan: 7605
Hua, Alice: 8020
Hua, Cassandra: 1535, 2185, 8010
Hua, Yindong: 3415
Huan, Tianwen: 8020, 8055
Huang, Alison: 1055, 2035, 8015
Huang, Alison J: 1245
Huang, Andrew W: 1530
Huang, Bin: 2190, 8015
Huang, Chenshan: 7450, 7930
Huang, Chenxi: 1115
Huang, Chongmei: 2040, 2255, 2500, 3330, 4055, 7440, 7440
Huang, Feng: 7275, 7710
Huang, Helen: 8015
Huang, Hsien-Hao: 8000
Huang, Liming: 4200, 7665
Huang, Min-Hui: 8010
Huang, Nuan-Ching: 7540
Huang, Qiyuan: 7670
Huang, Ro: 1475, 4415
Huang, Rui: 8015
Huang, Siou-Tang: 7670
Huang, Tianyi: 9020
Huang, Weidong: 1355
Huang, Wei-Jia: 7275
Huang, Xianging: 8000
Huang, Xiaoming: 7710
Huang, Yan: 7145
Huang, Yi-Jia: 7280
Huang, Yiqing: 7625
Huang, Zewen: 3480, 7255
Huang, Zideng: 2050
Hubbart, Chase: 4310
Huber, Lesa: 3530
Huberty, Jen: 1345
Hubig, Nina: 2355
Hubner, Sarah: 7675
Huckfeldt, Peter J: 1390
Hudes, Seth: 7720
Huebschmann, Amy: 8020
Hueluer, Gizem: 4185, 4525, 7355, 7535
Hugenschmidt, Christina: 7490
Hughes, Jaime M: 2145, 2475, 7685
Hughes, MacKenzie L: 7225
Hughes, Mark: 2540
Hughes, Michelle: 7440
Hughes, Tiffany F: 1485, 7145
Hughes, Timothy: 2220, 3180, 7010, 7015, 8020
Huh, Jimi: 4375
Huh-Yoo, Jina: 4220
Hui, Katherine: 7320
Huijsman, Robbert: 7445
Huisingh-Scheetz, Megan: 2570, 3150, 7205
Hule, Sunil S: 7430
Hulko, Wendy: 4025, 8010
Humber, Marika B: 3110, 7135
Humbert, Andrew: 7080
Hundza, Sandra: 7260, 7525
Hung, Arthur: 7020
Hung, Lillian: 1115, 2410, 2530, 3205, 7465, 7510, 7750, 7910, 8010, 8015, 8020
Hung, William: 2430, 8010
Hunt, Angela: 8015
Hunt, Lauren: 2020, 4080
Hunter, Heather: 7700
Hunter, Paulette V: 4195, 7055
Hunter, Ruth: 3125
Hunter, Sarah: 3170
Huo, Meng: 7005
Huo, Qun: 8015
Huo, Shutong: 2020
Huo, Zhiguang: 1445
Huot, Suzanne: 7300
Huppert, Theodore J: 2510
Hurd, Laura C: 7300
Hursh, Colleen: 2345
Hurtt, Barbekka: 7720
Huseth-Zosel, Andrea: 7090
Husser, Erica: 7410
Huth, Megan K: 1030
Hutto, Lashawn: 1465, 7350
Huxhold, Oliver: 2025, 4525
Huyen, Do Thi Thu: 7355, 8015
Huynh, Duong: 8015
Huynh, Mia: 7005
Huynh, Stephanie: 8025
Huynh, Tai: 8010
Huynh, Thinh: 7720
Hwan Chung, Tae: 7305
Hwang, Billie: 7625
Hwang, Boram: 7430
Hwang, Jeongeun: 8015
Hwang, Mina: 7235
Hwang, Misun: 7360
Hwang, Phoebe W: 8020
Hwang, Sinwoo: 7715, 7750
Hwang, Ula: 8010
Hwang, Yeji: 7235, 7435, 7630

Hyde, Eleanor R A: 4490
Hyde, Rebecca: 7055
Hyer, J. Madison: 4475
Hyer, Madison: 2445
Hyun, Jinshil: 7145, 7225
Hyun, Kate: 7155

I
Iaboni, Andrea: 4445, 9040
Iacob, Eli: 3500, 4520, 7425, 7905
Ibrao, Miquela: 7055
Idler, Ellen: 7530
Idu, Abisola: 8010
Iemoto, Naoki: 8015
Igarashi, Heidi: 7030
Igawa, Hitoshi: 7095
Igbokwe, Beatrice Chinwe: 4000
Igbokwe, Chima Charles: 4000
Ignácio Sossai, Mariana: 7225, 7655
Ihara, Emily S: 2415, 3510, 4540, 7270
Ihara, Kazushige: 7205, 7540
Iizuka, Ai: 1265, 7680
Ikebe, Kazunori: 7105, 7115
Ikeda, Naoki: 4135
Ikesu, Ryo: 1545, 7535
Ikeuchi, Tomoko: 7145
Iktilat, Khalil: 7695, 9040
Ikuta, Kasumi: 7510
Ilardo, Joan L: 7105, 7640
Iloputaife, Ike: 4535, 7450, 8000
Iluobe, Victory: 7000
Ilyas, Nazish: 4100
Im, Eun-Ok: 3370, 7270
Im, Jennifer: 8010
Im, Sua: 3050, 7280
Imamura, Keigo: 7205, 7540
Imms, Phoebe: 4310
In Der Schmitten, Jürgen: 2255
Inagaki, Hiroki: 7350, 7640
Indran, Nicole: 1425, 3015, 7155
Infurna, Frank J: 1190, 1450, 4065, 4500, 7265
Inger, Jacob: 7680
Ingle, Pilar: 7650
Ingram, Cory: 4415
Ingvalson, Stephanie: 8020
Inoue, Kosuke: 7535
Inoue, Megumi: 2370, 2415, 4190, 7095, 7270
Inouye, Sharon K: 4530
Intrator, Orna: 2430, 2545, 8020, 8055
Iob, Eleonora: 2365
Irani, Elliane: 2015, 7270
Irimia, Andrei: 1580, 4310
Irish, Stephanie: 7425
Isacson, Michal: 7695
Isales, Carlos: 7210
Isenberg, A. Lisette: 8015
Isenberg, Avital: 4530
Ishii, Keiko: 7340
Ishitake, Tatsuya: 7400, 7920
Ishiwata, Eric: 3575
Ishizaki, Tatsuro: 7115
Islaya, Cesz: 7490
Isola, Jose: 4310
Ito, Daisuke: 8020
Ito, Hiro: 4435
Ito, Kae: 7640
Ito, Koki: 7680
Itoh, Sakiko: 7750
Itotani, Keisuke: 7420
Iveniuk, James: 1110, 3410
Ivey, Kimberly: 4510
Iwarsson, Susanne: 1285, 3170, 7155
Iwasaki, Michiko: 7150
Iyer, Gitanjali: 1015
Iyer, Sandhya: 8010
Izumi, Seiko: 7440

J
Jaber, Sausan: 8000
Jablonski, Rita: 2375, 4045, 7525 8000
Jacelon, Cynthia: 7630, 8015, 8020
Jacklin, Kristen: 2335, 2395
Jackson, Ajani: 8020
Jackson, Alexandra: 2295
Jackson, Barbara: 8020
Jackson, Christian: 7350
Jackson, Christie: 4160
Jackson, Christine R: 4040
Jackson, Eva MJ: 7425, 7910
Jackson, Gary B: 4040
Jackson, Grace: 7010
Jackson, Heide: 7040
Jackson, Kathryn: 1570, 3220, 7455
Jackson, Kelley: 7360
Jackson, Kimethria L: 2550
Jackson, Lynn: 3370, 7465
Jackson, Phronie: 7030
Jackson, W. Clay: 8020
Jacob, Alexandra E: 7285
Jacob, Riya: 3010
Jacobs, Jeremy M: 7060
Jacobs, Josephine C: 2190
Jacobs, Leslie: 7425, 7930, 8070
Jacobs, M. Lindsey: 1210, 7070, 7455, 7610, 8015
Jacobs, Moyosoreoluwa: 7630
Jacobsen, Erin: 1485, 7145
Jacobson, Mireille: 7630
Jacova Chenoweth, Claudia: 1020
Jadczak, Agathe Daria: 7275
Jaeggi, Susanne M: 4490, 8000
Jaen, Carlos: 8050
Jafari, Mahtab: 7525
Jahoor, Farook: 2080
Jain, Abhinav: 3160
Jain, Felipe A: 4130, 7270, 7660
Jain, Jennifer C: 8020
Jain, Paavani: 8090
Jain, Shalini: 7210, 8015
Jajko, Shannon: 2450
Jakicic, John M: 1390
Jalil, Tanjina: 3365
Jallorina, Bianca: 7210
Jaman, Rafeeul: 8015
James, Bev: 3200
James, Bryan: 1570, 7205
James, Clara E: 4490
James, Dara L: 3015, 4010, 7285
James, Jennifer: 3420
James, Shirley A: 7480
Jan, Muhammad Tanveer: 7610
Jandric, Jakov: 2425
Janes, Heather: 7480, 7900
Janevic, Mary: 1155, 7030
Jang, Ah Ram: 7095
Jang, HaeJin: 8020
Jang, Heejung: 1525, 7630
Jang, Il-Young: 7305
Jang, Jae Young: 8000, 8010, 8015
Jang, Jeein: 2205, 3545, 7350, 8015, 8020
Jang, Jieun: 3135
Jang, Jinwoo: 7360, 7425
Jang, Jiwon: 8000
Jang, Soeun: 7155, 7340, 8010
Jang, Soog-nang: 7275
Jang, Woo: 8085
Jang, Yuri: 1140, 3440, 7640, 8000, 8020
Janke, Megan: 7545
Jankowski, Catherine: 7360
Jannati, Ali: 7425, 7635, 7930
Jansen, Carl-Philipp: 2170
Jansen, Taylor: 2480, 3265, 4105, 7065, 7240, 7635, 7695, 7700
Janssen, Leah M: 4510
Jantzi, Micaela: 7620
Jao, Ying-Ling: 7040, 7200, 8015, 9015
Jarke, Juliane: 3315
Jarmul, Cynthia: 4330
Jaros, Maria: 7650
Jarrín, Olga F: 1380, 4485
Jarrott, Shannon E: 2130, 2270, 3265, 7510
Jarvis-Powers, Robin: 7425
Jaskson, Christie: 4160
Jasper, Heinrich: 2310
Jasper, Lisa: 8010
Javier, Noelle Marie Coloma: 8010
Jawadi, Sagrika: 1370
Jayakody, Oshadi: 7750, 8000
Jazwinski, S. Michal: 7225
Jeamjitvibool, Thanakrit: 1505
Jean Jacques, Wildjena: 7110
Jeanblanc, Alexandra: 7265, 7660
Jee, Young Min: 8020
Jefferson, Rebeca C: 1505
Jeffroy-Meynard, Anne-Celine: 7200
Jehu, Deborah A: 8015
Jen, Sarah: 4250, 8010
Jenkins, Emerald: 1225, 2010, 2230
Jennings, Lee: 7480
Jennings, Stephen: 7080, 7155
Jensen, Christy J: 4470
Jensen, Leif: 4105
Jensen, Megan: 8015
Jensen-Battaglia, Marielle: 7420, 7920
Jeon, Ashley: 7010
Jeon, Gyeahyung: 4265
Jeon, Jaemin: 7695
Jeon, Sangha: 1350
Jeon, Yein: 8015
Jeong, Chung Hyeon: 7030
Jeong, Haelim: 7440
Jeong, Jihyeong: 7240
Jeong, Mijin: 7630
Jeong, Sol-ah: 8015
Jesmin, Syeda S: 1485, 7125
Jester, Dylan: 2185
Jeung, Jihong: 7035
Ji, Linying: 1250, 2055
Ji, Meihua: 7410
Ji, Minghui: 8000

Ji, Soo-Yeon: 7630, 7935
Ji, Sunghwan: 7305
Ji, Xiayan: 7145
Jia, HongFei: 7520, 7910, 9045
Jia, Jie: 7630
Jia, Lile: 8020
Jia, Shuli: 8015
Jia, Xueqing: 8015
Jiang, Da: 2520, 3480, 7255, 7725, 8000, 8020
Jiang, Jiabei: 7035, 7520
Jiang, Kening: 1055, 2525
Jiang, Lin: 1335, 2355
Jiang, Nan: 3305, 3540, 7235, 7630
Jiang, Nisi: 4310
Jiang, Shuangke: 4490
Jiang, Xiao-han: 1560
Jiang, Xinye: 7710
Jiang, Yan: 7425, 7930
Jiang, Yanping: 1380, 3245, 3440, 4365, 8020, 8050
Jiang, Yun: 7360
Jiang, Zhe: 4060
Jiangqiong, Li: 7465
Jimenez, Andreina: 8035, 8070
Jimenez, Daniel: 7540, 7735
Jiminez, Marcia P: 3180
Jin, Benjamin: 8000
Jin, Guoping: 1170
Jin, Hao: 3270
Jin, Haomiao: 2465
Jin, Liangyi: 3505
Jin, Qiao: 1355
Jin, Shiow: 7060
Jin, Tingan: 8010
Jin, Tongtong: 7265
Jin, Xianglan: 7465
Jin, Xiaotong: 7470, 7905
Jin, Yaping: 7440
Jing, Liwei: 7280
Jing, Xia: 2355
Jivraj, Stephen: 1450
João Forjaz, Maria: 4045
Joannette, Tania Valentine: 7645
Joblin, Celie: 7490
Johanning, Jason M: 3435
Johansson, Linda: 4010, 4335, 8015
John, Karen: 3345
John, Samantha: 7240
Johns, Michelle: 1110
Johnson, Alisa J: 1445
Johnson, Alisha H: 1530, 9025
Johnson, Benjamin B: 7605
Johnson, Eileen: 1090, 1240, 2220
Johnson, Elma B: 1260, 2155, 4235, 7640
Johnson, Florence U: 7665
Johnson, Ian M: 3170, 8080
Johnson, Jada: 1075
Johnson, Julene K: 7085
Johnson, Keith: 7500, 7930
Johnson, Khrystal: 8010
Johnson, Kim G: 7425, 7930, 8070
Johnson, Kimson E: 7630, 8020
Johnson, Maddie: 1260
johnson, mallory l: 8015
Johnson, Melanie L: 1485
Johnson, Michael P: 4460, 8010
Johnson, Nina: 4440
Johnson, Richard W: 2420
Johnson, Shawn M: 7465
Johnson, Theodore M: 1430
Johnson, Thomas: 4215
Johnson, Yuuko M: 2200
Johnson Nicholson, Meneka C: 1385
Johnson-Jennings, Michelle: 4110
Johnson-Toliver, Stephanie: 3140
Johnston, Elizabeth: 7060
Johnston, Joseph: 8010
Johnstone, Sara Lynn: 8010
Joiner, Raquael J: 3165
Jokisch, Mario R.: 7680
Jolliff, Anna: 7135
Jones, C. Allyson: 8010
Jones, Christine D: 4460, 8010
Jones, Clarence: 1265
Jones, Damon: 7145
Jones, David: 7520
Jones, Jennifer M: 7140
Jones, Liz: 4255
Jones, Nancy: 4215
Jones, Paige: 1535
Jones, Patricia: 1340, 2475
Jones, Richard: 3335, 4380
Jones, Sarah E: 4270, 7660
Jones, Shannon: 1295
Jones, Valerie: 4205
Jones Weiss, Deborah: 8020
Jones-Cobb, Brittany R: 2260
Jones-Locklear, Jennifer: 8015, 8020
Jonsson, Oskar: 3170, 7155
Jonsson, Palmi: 2040
Joo, Jinhui: 2315
Joo, Susanna: 7360
Jopp, Daniela S: 1220
Jordan, Aydan: 8000
Jordan, Claire: 1575
Jordan, Jacqueline: 1230
Jordan, Kerry J: 7040
Jor'dan, Azizah: 4535
Jordre, Becca: 8015
Joseph, Rodney P: 8020
Joshi, Sama: 8020
Joya, Mikaela: 7210
Ju, Catherine H: 7115, 7490
Ju, Wonkyu: 7015
Juang, Wang-Chuan: 7110, 7400
Juckett, Lisa: 7435
Judd, Suzanne: 8015
Judge, Katherine S: 1265, 4200, 8020
Juhasz, Audrey: 4435, 7270
Jump, Alayna: 7425
Jump, Robin: 2345
Jun, Hee-jin: 7300
Jun, Jung Sim: 7020, 7235
June, Andrea: 2440
Jung, Bock-Young: 8015
Jung, Dukyoo: 7005
Jung, Hailey H: 4495, 8010
Jung, Heeeun: 8000, 8010, 8015
Jung, Inhye: 7155, 7410, 7535
Jung, Lanee: 4515
Jung, Mi Sook: 7265, 8000, 8010, 8015
Jung, Miyeon: 8000
Jung, Wonkyung: 1365, 2010, 8015
Jung, Yeongin: 7470, 7905
Jung, Yunkyung: 7275
Junghaenel, Doerte U.: 2465
Jurczak, Michael: 8000
Juris, Jill: 1185, 2130
Jurivich, Donald: 8020
Jurkowski, Elaine T: 7480
Justice, Jamie N: 1330, 3045
Jutkowitz, Eric: 3535, 4245, 4340, 8010
Juturu, Preeti: 7510
Jylha, Marja: 7530
Jylhävä, Juulia: 7010

### K

Kaapu, Kristi: 4110
Kaasalainen, Sharon: 4195, 7055
Kabalan, Mayssan: 1155
Kabaya, Sonoko: 8015
Kabil, Gül G: 7205
Kabir, Ameera: 4300
Kabo, Felix: 4325
Kabua, Philmar M: 8000, 8015
Kackley, Madison: 8000
Kadi, Selma: 2060
Kado, Deborah: 1575
Kadowaki, Laura: 7740
Kaeberlein, Matt: 7010
Kafle, Bharat: 3055
Kahana, Eva: 1070, 1140, 4430, 7660
Kahsay, Eskira: 3235
Kai, Ichiro: 7490
Kaiksow, Farah A: 3335, 4530
Kaileh, Mary: 7210
Kailley, Jaya N: 7430
Kain, Nichole: 2460, 8010
Kainradl, Anna-Christina: 2320, 7085
Kaizi-Lutu, Marc: 1255
Kajitani, Shinya: 7130
Kaka, Baasil M: 3475
Kakara, Ramakrishna S: 1235
Kakulavar, Avinash: 7530
Kakuta, Momoho: 7115
Kalantari, Saleh: 3060
Kales, Helen C: 4130, 4340
Kalesnikava, Viktoryia A: 3245, 7695
Kaluna, Lia: 3140
Kalvesmaki, Andrea F: 2415, 2545
Kalyani, Rita: 7010, 7710
Kamalyan, Lily: 3065
Kamanga, Foster: 7660
Kamath, Vidyulata: 4440, 7285
Kameyama, Mayumi: 8015
Kamide, Kei: 7115, 8020
Kaminski, Margi: 4325
Kaminski, Naftali: 4170
Kan, Denise: 7440
Kananen, Laura: 2460
Kane, Alice E: 3520, 7275
Kane, Kevin: 7695, 7935, 8010
Kane, Tanya: 1155
Kaneshiro, Jasmine: 2530
Kang, Bada: 7080, 7275, 7630, 7640, 8015
Kang, Chaeeun: 7275, 8015
Kang, Chaeryon: 4315
Kang, Hun: 7680
Kang, Jee eun: 4405, 4515
Kang, Ji young: 4265
Kang, JungHee: 3320
Kang, Miseon: 7155, 7410, 7535
Kang, Ning: 3050, 7680, 8085
Kang, Rui: 1215, 8010
Kang, Sarah: 8010
Kang, Simeon C: 7480, 7900

Kang, Sophia: 3350
Kang, Suk-Young: 7360
Kang, Sungwan: 7430
Kang, Sunny: 7515
Kang, Yixin: 2240
Kang, Youjeong: 8000
Kang, Yu Jin: 1230
Kanter, Jeremy: 7070
Kao, Hsueh-Fen S: 1175
Kapadia, Farzana: 4225
Kapilan, Kaya: 2510
Kaplan, Celia C: 1245
Kaplan, Daniel B: 3175
Kapoor, Akshat: 7360
Kappler, Caitlin B: 2145, 7685
Karakida, Maki: 2205, 8010, 8015
Karakose, Selin: 7245, 7350
Karam, Rita: 7350, 7475
Karas, Marta: 8015
Karasik, Rona J: 2490, 7480
Karawani, Hanin: 3085
Karbakhsh, Mojgan: 7405
Kårelind, Fanny: 4010
Karczewski, Hannah A: 7420, 7920
Karel, Michele: 1080, 7695
Karere, Genesio: 8000
Karikari, Thomas K: 3375
Kariya, Hitomi: 3065
Karki, Dinesh: 7485
Karlawish, Jason: 7205, 8015
Karliner, Leah S: 3010, 4230, 7325
Karlsen, John: 8010
Karmacharya, Isha: 4015
Karon, Sara: 4305
Karr, Daniel W: 7665
Kartoz, Connie R: 1505
Karve, Aadit: 7210
Karvonen-Gutierrez, Carrie: 1365, 1435
Kasahara, Sachiko: 7600
Kasarskis, Edward J: 7245
Kasenetz, Whitney: 7610
Kaskie, Brian P: 3115, 3310, 3495, 4395, 8075
Kasler, Kamryn: 1160
Kaspar, Roman: 1285
Kasperovich, Natalia: 8010
Kassay, Abraham: 7540
Kassem, Moustapha: 7010
Kastner, Keri: 2480, 3255, 7510, 7915
Kasuga, Ayaka: 8020
Katagiri, Keiko: 2100, 7030, 7540
Katare, Bhagyashree: 4115, 7660, 8015
Kats, Allyson: 4055, 7630
Katsumata, Asako: 4135, 7405
Katz, Ben: 4315, 7615, 8020
Katz, Mindy J: 2160, 7225, 7455, 7630
Katz, Ruth: 4040
Katz, Stephen: 2265, 4275
Kauenhowen, Cornelia: 1480, 2440
Kaufman, Brystana G: 1475, 2180, 4415
Kaufmann, Christopher N: 1255, 1445, 2290, 7225
Kaur, Jagbir: 7465
Kaushal, Navin: 3050, 3235
Kautz, Tiffany: 7735
Kavanaugh, Melinda S: 2415
Kavcic, Voyko: 8000
Kawai, Hisashi: 1505, 7205, 7540
Kawakami, Keilyn L: 7055
Kawakami, Shinpei: 8015
Kawasaki, Teruaki: 8010
Kay, Denis G: 8015
Kaya, Alaattin: 7005, 7210, 8000
Kaye, Jeffrey: 3140, 3285, 7020, 7735, 8020
Kaye, Lenard W: 7415
Kayser, Jay: 8020
Keane, Connor Thomas Francisco: 8090
Keating, Norah: 1120
Keatings, Margaret: 4255
Keaulana-Scott, Samantha: 8020
Kebbede, Liyat Y: 8000
Keefe, Bronwyn: 2455, 7475, 7650
Keefe, Janice: 2495, 7420
Keefe, Kate: 1345
Keenan, Teresa A: 1085
Kehne, Theresa H: 1480, 7200
Kehrer-Dunlap, Abigail: 7655
Keisari, Shoshi: 8010
Keiser, Cassie: 1160
Kelekar, Uma: 1235, 3505
Keleman, Audrey: 7655, 8015
Kelle, Nadiya: 2025
Keller, Abiola: 2555
Keller, Michelle S: 7320
Keller, Valerie: 2320
Kellett, Kathy G: 7740
Kelley, Amy S: 1340, 2210, 3145
Kelley, Heather: 8020
Kelley, Jessica A: 1180, 2350, 3065, 3385, 3425
Kelley, Johnna L: 7695
Kelley, Lynette: 2430
Kellogg, Dean Lundt: 3350
Kelly, Christine: 4450
Kelly, Christopher M: 2275, 4290, 7510
Kelly, CJ: 7010
Kelly, Kathleen: 2200
Kelly, Megan: 8015
Kelly, Monica R: 7735
Kemp, Blakelee: 2120
Kemp, Candace L: 4290, 8010, 8020, 8070
Kemp, Elizabeth C: 2485
Kendall, Jaiden: 7300
Kendzor, Darla E: 7330
Kennedy, Katherine A: 1080, 4460, 7250, 7545
Kennedy, Kimberly A: 1410
Kennedy, Meaghan: 2430, 8010
Kennedy, Quinn: 7715
kennedy, Scott: 7075
Kennedy, Teri: 2495
Kennedy, Winston: 8015
Kennedy-Malone, Laurie: 7275
Kennel, Victoria: 8015
Kent-Thomas, Kaylin: 7350
Keny, Christina: 4045
Kenyhercz, Michael: 7235
Kerac, Marko: 4455
Keri, Ben: 8000
Kern, Kristopher C: 1325
Kerper, Shaye A: 3045
Kerr, Haiming: 7305
Kersten, Alan W: 7225
Kessler, Claudette M: 1250
Kestle, Dorothy: 2395
Ketcher, Dana: 2335, 2395
Ketel, Christian: 9050
Key, Kent: 3170
Kezios, Katrina: 1545
Khadeer, Mohammed: 8000
Khadka, Aayush: 2020
Khairallah, Ramzi J: 8000
Khakharia, Anjali: 7220
Khalighi, Mehraneh: 3435
Khalikov, Marla: 8010, 8015
Khalil, Mohammad: 7000
Khalilian, Mohammad: 1480
Khamzina, Madina: 8000
Khan, Aishah: 2355
Khan, Maham: 4110, 4315, 8010
Khan, Mushira M: 1215, 4015, 7505, 7935
Khan, Nadia: 7665
Khan, Nibraas: 7155
Khan, Sadiya: 8000
Khani, Saber: 1280
Kheroua, Sirine: 8020
Khetir, Imene: 7645
Khoo, Shuna S.: 8020
Khoo, Shuna Shi Ann: 7155
Khor, Ern Chern: 7155
Khov, Brian: 7015
Khundmiri, Syed: 7000
Khvatova, Elizabeth: 2145
Kiani, Shirin: 1175
Kidd, Bethany: 7155
Kidner, Madison: 2325
Kiefer, Jamie: 8000
Kieffer, Elana F: 2460, 3485
Kiel, Douglas P: 1275, 2080
Kiely, Kim: 3185
Kietzman, Kathryn G: 7510
Kiiti, Krungkraipetch: 7460
Kilaberia, Tina R: 1270, 7145
Kilcrease, Amanda K: 1060
Kilgore, Zach: 1295, 4265
Kilkey, Majella: 3550
Killian, Emily A: 2185, 3485
Killian, Tim S: 2550
Kilmer, Greta: 7065
Kilpatrick, Abbey: 7435
Kilpatrick, Abigail: 7155
Kilpela, Lisa: 7720
Kim, Andy J: 7225
Kim, Boah: 7740, 8010
Kim, Boeun: 3140, 7445, 7465, 7935
Kim, BoRin: 1295, 7030, 7265, 7290, 7630, 7665, 8010
Kim, Chang-O: 7275
Kim, Dae: 2080
Kim, Dae Hyun: 3135, 4225, 7080, 7275, 7280, 8015
Kim, Daejin: 4325
Kim, DaEun: 8000
Kim, Dahee: 2300, 7025, 8000, 8015, 8020
Kim, Dahye: 3545
Kim, Daniel: 9030
Kim, Do Yun: 7545
Kim, Dongmi: 3370, 7270
Kim, Dylan: 7750
Kim, Eric S: 1545, 4550
Kim, Eunbea: 4060, 4095
Kim, Eunhye: 7030
Kim, Eunjung: 3055
Kim, Eunkung: 7150
Kim, EunKyo: 7295, 7715
Kim, Eunsaem: 7275, 8015
Kim, Eunyoung: 7310

Kim, Gee: 7470
Kim, Giyeon: 7225, 7275, 7535
Kim, Gwang Suk: 7260, 7275, 7290, 7295, 7680, 8015
Kim, Ha Neul: 2265, 7745
Kim, Haesung: 8010
Kim, Haeun: 8000
Kim, Haklyoung: 7430
Kim, Haneul: 3270
Kim, Hansol: 7725
Kim, Heewon: 8010
Kim, Hokon: 7440
Kim, Hyejoong: 7720
Kim, Hye-Mi: 7460
Kim, Hyosin: 1380, 4485
Kim, Hyun Jung: 7705, 7720, 8020
Kim, Hyungjin Myra: 7490
Kim, Hyun-Jun: 2540, 4495, 8010
Kim, Hyunmi: 8010
Kim, Jaehyun: 8015
Kim, Jaein: 3370
Kim, Jasmyn: 8080
Kim, Jay: 8015
Kim, Jayeong: 7435
Kim, Jeehoon: 7355, 7625
Kim, Jennifer Ivy: 7080, 7275, 7630, 8015
Kim, Jenny: 1140
Kim, Jeongsuk: 3440
Kim, Jeung Hyun: 3030, 8020
Kim, Jeungkun: 7360
Kim, Jinhee: 8015
Kim, Jinho: 3360
Kim, Jinyoung: 4535
Kim, Jiyeon: 1395
Kim, Julie S: 7405, 7660
Kim, Jung Hyun: 9010
Kim, Jung Ki: 1455
Kim, Jung Yeon: 7645
Kim, Junhyoung: 7750, 8015, 8015
Kim, Kahyun: 7005
Kim, Keunyoung: 7015
Kim, Kiyeon: 7310
Kim, Korinek: 4050
Kim, Kwang-il: 8015
Kim, Kyeongmo: 7630, 7935
Kim, Kyungmin: 1360, 2065, 3545, 7005, 7460, 7720, 8000
Kim, Layoung: 7260, 7275, 7290, 7680
Kim, Manjun: 2265
Kim, May: 7280
Kim, Meeryoung: 7095
Kim, Miji: 7275, 8000, 8010, 8015
Kim, Min Hee: 2020
Kim, Min Hee: 2020
Kim, Min Jung: 8015, 8020
Kim, Minsub: 8015
Kim, Nahyun: 2100, 7460, 7540
Kim, Namhee: 7275, 7290, 7680
Kim, Nani: 7145
Kim, Nayeon: 7040
Kim, Oksim: 7440
Kim, Patricia S: 1395
Kim, Richard: 8015
Kim, Sangkyu: 7225
Kim, Sarah: 8000
Kim, Seo Young: 8010
Kim, Seohyeon: 8020
Kim, Seokmin: 1475
Kim, Seon: 7535, 7630, 7935
Kim, Seoyoun: 2425, 7355, 8020
Kim, Seoyoung: 8010
Kim, Sewon M: 7270
Kim, Seyoung: 8000
Kim, Sohee: 2375
Kim, Sohyun: 1455, 3400, 4045
Kim, Soo Hyun: 7055
Kim, Steffi M: 3005, 3345, 3585, 4015, 4320, 8070
Kim, Su Hyun: 7705, 8085
Kim, Su-Hwan: 3125
Kim, Suk-hee: 2455
Kim, Sukyung: 7275, 8015
Kim, Sun Ju: 7220
Kim, Suyoung: 8020
Kim, Taeeun: 7265
Kim, Tasha: 4420
Kim, Yeonju: 7360, 7750
Kim, Yeri: 8010
Kim, Yesol: 7360, 7750
Kim, Yijung K: 7640, 8020
Kim, Yongseop: 7750
Kim, Yoon Chung: 1070, 2000, 3565
Kim, Youngran: 3135
Kim, Yubeen: 8015
Kim, Yulri: 1070, 4060
Kimball, Patricia: 2150
Kim-Knauss, Yaeji: 1375
Kimmel, Jacob C: 2310
Kimmel, Susan: 8020
Kimpel, Christine C: 9050
Kimzey, Michelle: 4240
Kincaid, Reilly: 7265
Kincaid, William: 3340
Kindratt, Tiffany B: 3055, 7530
King, Abby C: 4000
King, Angelique: 1190
King, Anne E: 4160
King, Barbara J: 4075, 8015
King, Christina D: 3045, 4170
King, Deborah: 4230
King, Jessica: 3570
King, Natalie: 4255
Kinkade, Emily E: 7605
Kinnear, Lana B: 7255
Kinney, Jefferson: 4535
Kinoshita, Ayae: 8010
Kinosian, Bruce: 2545, 4535
Kinugawa, Arisa: 1115
Kinzig, Elizabeth: 8020
Kiosses, Dimitris: 7630
Kirby, Mackenzie E: 7475
Kirch, Matthias: 1085
Kircher, Julie A: 7255
Kirchner, Kathleen A: 7005, 7465
Kirk, Jeanette W: 4075
Kirkland, Jim: 8000
Kirsch, Jaclyn: 2195
Kirschner, Kristina: 3325
Kirsh, Jaclyn: 1105
Kirvalidze, Mariam: 4045, 7215
Kishimoto, Kyoko: 7480
Kissell, Paul E: 7640
Kitago, Moe: 7680
Kitamura, Akihiko: 7275
Kitamura, Koji: 1505
Kitayama, Atsushi: 7420
Kitayama, Jun: 7095
Kitchen, Courtney: 1130
Kitchen Andren, Katherine: 7490
Kitchens, Katherine: 2195, 4400
Kittle, Krystal R: 2015, 2225, 3190
Kittleman, Cory: 7530
Kitt-Lewis, Erin: 2140
Kitto, Elizabeth S: 4005
Kitzman, Dalane: 7710
Kiwak, Eliza: 7085
Kizony, Rachel: 7300
Klaiber, Patrick: 4405, 4410
Klank, Christina: 7480
Klasen, Lisa Joanne: 2025
Klatt, Maryanna D.: 7490, 7935
Klenczar, Brittany: 2225, 3490
Klenk, Jochen: 2170
Klepacz, Laura M: 1525, 7030, 7455
Kleszynski, Keith: 7480, 8015
Kleykamp, Bethea A: 9040
Kliegel, Matthias: 4490
Klimstra, Marc: 7260
Kline, Caitlyn: 7625
Kline, Chelsea: 3000, 7005
Kline, Christopher E: 1365
Klinedinst, Tara C: 7330
Klinger, Christopher: 7480, 7900
Klokgieters, Silvia: 1120
Klopack, Eric T: 3095, 8060
Klug, Marilyn: 8020
Klunk, William: 9000
Knauft, Katherine M: 7540
Kneale, Dylan: 1110
Knebl, Janice: 1065, 3510
Knecht-Fredo, Jennifer: 7410
Knickerbocker, Kelly J: 1265, 7075
Knight, Erik L: 7210
Knoefel, Frank: 3285
Knoepke, Christopher: 3320
Knox, Sara: 1075
Knutson, Kristen: 2290
Ko, Eunjung: 7660, 8020
Ko, Fred C: 3435
Ko, Jung Eun: 3440
Ko, Linda K: 1140, 1480, 7200
Ko, Sunghyun: 8010
Ko, Woorim: 4070
Ko, Young: 8010
Koçer, Rüya: 2565
Kobayashi, Lindsay: 1290, 2515, 3145, 4380, 7140, 7420
Kobayashi, Tetsuro: 7360
Kobayashi-Cuya, Kimi E: 1265
Kobylarz, Fred A: 4485
Koch, Jana: 7610
Koch, Mary Kate: 7020
Kociolek, Judyta: 2300
Koehn, Sharon D: 8010
Koenigshoff, Melanie: 2435
Koeuth, Sokha: 4340
Koffer, Rachel E: 1350, 3080, 4010, 8020
Koffer, Rachel: 8000
Koffski, Maya J: 4235
Koget, Jennifer: 8015
Koh, YoungBin: 8010
Kohl, Gianna: 8000, 8035
Kohlbrenner, Briana L: 7495
Kohler, Hans-Peter V: 1280, 2425
Kohler, Iliana V: 1280
Kohli, Amarpreet S: 7680
Kohli, Maulika: 7675
Kohli, Nidhi: 3490

Kohlrausch, Estela: 3155
Kohon, Jacklyn: 2185, 4510, 8010
Koike, Teruhiko: 7315
Koios, Daniela: 7110
Koirala, Binu: 7440, 7500
Koizumi, Naoru: 2370
Kojima, Narumi: 8015
Kojis, Daniel: 7500, 7930
Kokinakos, Angela: 8010
Kolanowski, Ann M: 2140
Kole, Debarati: 3405, 7070
Koletar, Susan L: 7285
Kollasserry, Felix Joy: 9000
Kolpakov Nikitin, Stanislav: 7200
Kolpina, Lola: 3465, 4045, 8020
Komalasari, Renata K: 4315
Komisar, Harriet: 1085
Kong, Dexia: 4115, 7035, 7290, 8020
Kong, Jooyoung: 2001, 3225, 7660
Kong, Weijuan: 7640
Konlan, Kennedy Diema: 8010
Kono, Ayumi: 7080, 7105
Kontor, Okyere D: 8000
Kontos, Pia: 4445, 7040, 7055
Koon, Lyndsie: 8020
Kooshiar, Hadi: 8015
Kopera-Frye, Karen: 3345, 3585
Koppel, Sjaan: 3455
Kops, William J: 1480, 2440
Koren, Chaya: 3465
Korer, Burton: 8015
Korfage, Ida J: 2255
Korinek, Kim: 1485, 8020
Korlat, Selma: 2300
Kornadt, Anna E: 4020, 7610
Koronowski, Kevin: 7000
Kosar, Cyrus: 1230, 8075
Koss, Catheryn: 1145
Kossowska-Kuhn, Dorota: 8000
Kostelic, Amy: 1480
Kosteniuk, Julie: 7135
Koster, Annemarie: 1245
Koszalinski, Rebecca S: 2300, 7610
Kotecho, Messay G: 7540
Kothari, Meeraj: 4550
Kotkowski, Kornelia: 4255
Köttl, Hanna: 3425
Kotwal, Ashwin: 2020, 2520
Kou, Minghao: 7460, 7715
Kouame, Gail: 8010
Koufacos, Nicholas: 2280
Kouts, Holly: 1490
Kowal, Paul: 8015
Kowalchik, Kalei H: 1080, 8090
Koyama, Toshihiro: 7260
Kozlov, Elissa: 7110, 7360
Kozlov, Elissa K: 8035
Krämer, Michael D: 1215
Kraemer, Brian C: 7310
Kraemer, Kyle: 8020
Krafft, Jennifer: 3575
Kramer, B Josea: 2280, 7730
Kramer, Jenna: 7350, 7475
Kramer, Joel: 8020
Kramer, Philip A: 2080, 4005
Kraschnewski, Jennifer: 1080
Krassikova, Alexandra: 4255, 8015
Krauss, Melissa: 7260, 8015
Kravchenko, Julia: 3010, 3085, 7300, 7620
Kravljaca, Nikolina: 4295
Kreimer, Simion: 8000
Krekula, Clary: 2425
Krenek-Schneider, Brittany: 9050
Kreps, Gary L: 7360
Kriebernegg, Ulla: 2320, 3320, 4285, 7085
Kringle-Baer, Kirsten: 2325
Kritchevsky, Stephen: 1090, 1240, 1410, 2080, 2220, 2505, 3045, 4005, 7015, 7060, 8000
Kroenke, Kurt: 4440
Krok-Schoen, Jessica L: 2445, 3220, 7265
Kroplewski, Renee: 8020
Kropp, Denise: 7475, 7900
Krueger, Tillmann HC: 4490
Krull, Drew A: 8085
Krysko, Alyssa: 7140
Krywe, April: 8060
Ku, Hok Bun: 7530
Ku, Te-Lien: 7705
Ku, Vivian: 7535
Kuai, Zheng: 1435
Kuan, Yi-Chun: 7735
Kuan, Yun-Han: 8015
Kuang, Jiahao: 7710
Kubzansky, Laura: 4440, 4550, 7275
Kucharska-Newton, Anna: 2525, 4440, 7060, 7630, 8015
Kuchel, George A: 1275, 2495, 3210, 4350
Kuehn, Kevin: 7660
Kuhl, Melanie: 7360
Kuhl, Olivia: 8015
Kühn, Simone: 7210
Kuipers, Allison L: 7010, 7625
Kulkarni, Snehal S: 4055
Kullgren, Jeff: 1085
Kulminski, Alexander: 1025
Kuluski, Kerry: 8015
Kum, Cleopatra: 1465, 3455
Kum, Crescentia: 3455
Kum, Flavius: 3455
Kum, Mirabelle: 3455
Kuma, Mischa: 4370
Kumar, Amiritha: 8020
Kumar, Amit: 1075, 4385
Kumar, Chandrika: 7475
Kumar, Premranjan: 1420, 2080, 2235
Kumar, Sandeep: 7210
Kumar, Vivek: 7210
Kumari, Neha: 1195
Kumthekar, Nihar: 7680
Kuniholm, Mark: 8010
Kunik, Mark E: 4340
Kunkel, Miranda: 1160
Kuntajak, Premgamon: 7725
Kunzel, Carol: 8010
Kuo, Chun-Tung: 7460
Kuo, Fang-Lin: 4110, 7655
Kuo, Han-Yueh: 7080
Kuo, Tony: 7015, 7200
Kuo, Tsuann: 7040, 7055, 7070, 7280, 7335
Kuo, Yong-Fang: 2090, 4095, 7405, 8010
Kupferschmidt, Anthony, L.: 8010
Kurth, Maria: 2385, 4405, 7030
Kuryk, Kaitlyn: 2495, 7420
Kurzman, Alissa: 7325
Kusmaul, Nancy: 2000, 2470
Kuspinar, Ayse: 7655
Kutney-Lee, Ann: 1160, 4195
Kuy, Kakada: 4050
Kuzmik, Ashley: 3230
Kvale, Elizabeth: 2090
Kwak, Jacqueline R: 1440
Kwak, Judy J: 8020
Kwak, Jung: 2090
Kwak, Min Ji: 3135
Kwiatek, Susan: 8015
Kwiecien, Robert: 8015
Kwoh, kent: 8010
Kwok, Jojo: 2520
Kwon, Ester J: 7310
Kwon, Eunsun: 4265, 8010, 8010, 8010
Kwon, Ilan: 7695
Kwon, Jenny: 7295
Kwon, Simona C: 8095
Kwon, Soonhyung: 3440, 4365
Kwon, Sooyoung: 8015
Kwong, Katherine: 3515

L

Labbé, Angéline: 8020
Lachman, Margie E: 1525, 4065, 4500, 7455, 7610
Lachmann, Mark: 7530
Lachs, David: 7205
Lachs, Mark: 1270, 2150, 4365, 7205
LaCoss, Jennifer: 8010, 8015
LaCroix, Andrea Z: 8010
Ladiges, Warren: 7075, 8000
Laffer, Alex: 7475
Laflamme, Maeva: 7475
Lafontant, Kworweinski: 3055, 7640, 7720, 7735, 8000, 8015
Laforest, Justin: 7720
Lagacé, Martine: 3320
Lahar, Cindy J: 7415
LaHue, Sara C: 2530
Lai, An Chi: 7040
Lai, Daniel W.L.: 2050, 2150, 8050
Lai, Hsiu-Yun: 7080
Lai, Patrick HL: 2555
Lai, Patrick Ho Lam: 4140, 7355
Lai, Philip: 3550
Lai, Wenhua: 3165, 7065
Lai, Wen-Hua: 2015
Laird, Barrett: 7680
Laiton Jimenez, Laura: 7455
Lajambe, Lexie: 7720
Lakatta, Edward G: 8015
Lake, Jordan: 7285
Lakomý, Martin: 3360
Lakomy, Martin: 3190
Lakshmanan, Sarada S: 8020
Lalani, Nasreen: 4115, 7660, 8015
Lalwani, Anil K: 7140
lam, Jamie: 8015
Lam, Julia: 4100
Lam, Justin W: 3415, 8015
Lam, Michael: 7015
Lam, Michelle: 8015
Lam, Phillip: 9025
Lamba, Saanvi: 7360, 8010, 8070
Lambrou, Nickolas: 2335
Lampe, Nik M: 3075, 9030

Lampenfeld, Nathan: 2480
Lampkin, Cheryl L: 1085
Lanba, Asheesh: 7680
Landman, Bennett A: 2510
Landvoigt Schmitt, Brenda: 8000
Landwehr, Samantha: 8015
Landy, Ania: 3370
Lane, Nancy: 4005
Lane-Nelson, Nia R: 8080
Lang, Frieder R: 1375
Langa, Kenneth: 1290, 2515, 3145, 4380, 7350, 7490, 9010
Lange-Maia, Brittney: 1235
Langsetmo, Lisa: 4055, 7630
Langton, Lynn: 1370, 2150, 3270
Langworthy, Benjamin: 4245
Lanzi, Alyssa M: 3000
Lanzkron, Sophie: 8000
Lapane, Kate: 7060, 7275, 7320, 8015
Lapidus, Jodi: 7300, 8000
LaPierre, Tracey: 2500
Lapins, Allison: 7220
Lapointe, Mia: 8020
Lariviere-Chartier, Julien: 3285
Larkin, Marni J: 7330
Larkina, Marina: 7525, 7630, 7725
Larnyo, Ebenezer: 7020, 9000
LaRocca, Thomas: 7075
LaRowe, Lisa R: 9020
Larsen, Matthew W: 8015
Larson, Carol: 1575
Larson, Eric B: 3065
Larson, Janet L: 7435
Laskow, Thomas: 1205
Lasseter, Amanda: 8000
Latham, Amanda: 7075
Latham-Mintus, Kenzie: 3065, 8000
Latimer, Abigail: 3320, 7215
Lau, Terence K: 7695
Lauber, McKenzie: 7270
Lauck, Jen: 8020
Lauderdale, Diane S: 1110
Laukka, Erika Jonsson: 4315
Launer, Lenore: 2040, 9020
Laura, Bradbury: 7640
Laura Oliveira Dias, Ana: 7225
Laurienti, Paul J: 1150
Lavangu, Vaishali: 3235
Lavari, Kavin: 7270
Lavoie, Patrick: 7645
Law, Makenna: 8000
Lawrence, Aneesah: 8020
Lawrence, Joanna: 7750
Lawrence, Molly: 4515, 7040
Lawton, Alexander: 2520
Lawton, Dylan RY: 7080
Lay, Jennifer: 7725, 8000, 8020
Layman, Shannon: 2415, 7270
Le, Bao Ngoc: 8025
Le, Christine: 7005
Le, Elaine T: 4430
Le, Minh Quan: 7455
Le, Qun: 3245, 7260, 7695, 7935, 8010, 8050
Leach, Bethany: 8010
Leach, Carrie: 4320
Leach, Susan J: 7330, 8015
Leath, Seanna: 8080
Leavey, Gerard F: 2055
LeBrasseur, Nathan: 2005, 2435, 4005
Ledermann, Thomas: 7350
Lee, Adrian KC: 1175
Lee, Ahrim: 8010, 8015
Lee, Alexandra K: 3330
Lee, Boram: 9030
Lee, Chae Man: 3265, 7065, 7240, 7740
Lee, Chaejeong: 2260
Lee, Chaiwoo: 7065
Lee, Chanam: 7040
Lee, Cheryl A: 7525
Lee, Chioun: 1540
Lee, Chiyoung: 8010, 8015
Lee, Christopher S: 7440
Lee, Chung-Sheng: 7275
Lee, Daehyun: 8000, 8010, 8015, 8015
Lee, Dahyeon: 8015
Lee, Ellen E: 4180
Lee, Eun Jeong: 1140
Lee, Eunju: 3545, 7005
Lee, Gina: 1385, 3225, 3405, 4095
Lee, Haerim: 7265
Lee, Hajin: 8020
Lee, Hanna: 7205, 7440
Lee, Harold H: 1545, 8045
Lee, Heather: 8010
Lee, Hedwig E: 7350
Lee, Hee Y: 8020
Lee, Hi-Woo: 1140
Lee, Hyangkyu: 8010, 8015
Lee, Hye Soo: 3415, 8000
Lee, Hyeyeon: 7275
Lee, Hyo Jung: 7095, 7155, 7265, 7410, 7535
Lee, Hyunjoo: 3200
Lee, Hyunmin: 8000
Lee, Inheok: 7280
Lee, Insuk: 8015
Lee, Insup: 7145
Lee, Jae Jun: 7260, 7275, 7290, 7680
Lee, Jaesung: 7155
Lee, Jane: 2470
Lee, Jean Mui Hua: 8015
Lee, Jennifer A: 3010
Lee, Jenny Jinyoung: 8010
Lee, JeongEun: 1070, 1165, 1335, 3575, 4060, 4095, 7145
Lee, Ji Hyun: 1495, 7355
Lee, Ji Yeon: 7260, 7290, 7640, 7680, 7750
Lee, Jieun: 2145
Lee, Jinkook: 2040, 2515, 3125, 3335
Lee, Jongwon: 8000
Lee, Joseph H: 1025
Lee, Juhee: 7310
Lee, Juhyeong: 7225
Lee, June: 7145
Lee, Juneyoung: 1460
Lee, Jung-Ah: 1165, 2340, 3460, 7300, 7660, 7705
Lee, Jungjoo: 7750
Lee, Kathy: 7155, 7340
Lee, Kyoung Hag: 7020, 7235
Lee, Kyuho: 7695
Lee, Kyung Eun: 4505, 7070
Lee, Kyung Hee: 7295, 7420, 7645, 7750
Lee, Kyungmi: 8010
Lee, Kyuri: 2550, 7005, 7245
Lee, Lewina O: 4375
Lee, Lewis: 7440
Lee, Matthew: 8045
Lee, Mi-Jeong: 7625
Lee, Mina: 7545
Lee, Minyong: 8010
Lee, Mitchell: 7010
Lee, Myungeun: 7295, 8015
Lee, Paul: 4300
Lee, Pei-An: 4215
Lee, Rokbit Sanghee: 7280
Lee, Sang E: 3545, 7425
Lee, Sei J: 3330, 4230
Lee, Seolah: 7155, 7265, 7410, 7535
Lee, Seonhwa: 3190, 8085
Lee, Seungkeun: 8020
Lee, Shinduk: 2060
Lee, Soo-Kyoung: 8015
Lee, Soomi: 1255, 1350, 2290, 3275, 3525, 7130, 7455, 7735, 8000, 8020
Lee, Sophia: 7265
Lee, Sun Ah: 7255, 7360
Lee, Sung-Jin: 8010
Lee, Sungkyu: 8010
Lee, Tae-Ho: 8020
Lee, Ya-Wen: 7510, 8010
Lee, Yeong Ju: 7430
Lee, Yeonjung: 1100, 1195, 7605, 8000, 8010, 8020, 8045
Lee, Yeonjung J: 4445, 7600, 8000, 8000
Lee, Yeonjung Jane: 1310
Lee, Yeon-Shim: 7425, 7500
Lee, Yeonwoo: 8015
Lee, Yoojin: 8020
Lee, Yookyong: 8010
Lee, Youjung: 7410
Lee, Young-Shin: 7300, 7705
Lee, Yunhwan: 7275, 8015
Lee, Yura: 7070, 7425, 7540, 7930, 8090, 9010
Leedahl, Skye N: 1185, 2130
Lees Haggerty, Kristin: 1260, 1270, 4355
Lee-Turner, Meosia: 7030
Leff, Bruce: 8010
Leger, Kate A: 7255
Leggett, Amanda N: 2015, 3165, 7030, 7065
Lehning, Amanda: 1295
Lei, Lianlian: 7490
Leiderman, Alexis: 7140
Leinbach, Carla B: 8000, 8010
Leis, Aleda: 1435
Leiser, Scott F: 4005
Leist, Anja K: 9010
Lekan, Deborah: 7275
Lekhak, Nirmala: 1140, 7660
Leland, Natalie E: 4100, 7080
Lemack, Bergen: 1065
Lemonde, Manon: 3010
Lemus, Armando: 7000
Lenchik, Leon A: 2505
Leng, Xiaoyan I: 1410, 4005
Lentz, Jeffrey: 8020
Leonard, Chelsea: 8010
Leonard, Delaney: 8020
Leong, I. Tek: 1380
Leopold, David: 7680
Lepore, Michael J: 1315, 2000, 2350, 3565, 4465
Lero, Christi M: 7310

LeRoy, Angie: 9030
Lervick, Dana: 3115
Lester, Barbara R: 8010
Leung, Angela: 7680, 7695
Leung, Lok Man: 4365, 8020
Leung, Peggy: 8015
Leung, Vania: 7270
Leutwyler, Heather C: 1530, 2530
Levan, Darius T: 8040
Levasseur, Melanie: 1100, 3320, 4270, 8020
Levchenko, Yuliana: 1230
Levenson, Robert W: 3285, 3355, 8020, 8040
Levin, Michael: 4435, 7270, 8020
Levine, David: 8010
Levine, Meredith: 8010
Levine, Stacie: 7250
Levinsky, Michal: 2025
Levitan, Eric: 2485
Levy, Becca R: 2305, 3165, 4235
Levy, Cari: 8010, 9040
Levy, Helen: 7490
Levy, Sorah: 3090, 3255, 4090, 8020
Lewis, Charity T: 7140
Lewis, Frances M: 8015
Lewis, Hannah M: 7605
Lewis, Jordan P: 2335, 3005, 3345, 3585, 4015, 4320
Lewis, Jordan: 8070
Lewis, Lisa M: 7440, 7925
Lewis, Nathan A: 8000
Lewis, Nicole: 7660
Lewis, Stuart: 2150
Lewis Thames, Marquita: 4480, 4370
Leykum, Luci: 2545, 8070
Li, Aaron C: 8010
Li, Ashlee: 7425
Li, Brookelle: 3175
Li, Chen: 2500
Li, Chenxi: 2525, 7090, 7630
Li, Chien-Ching: 7080, 8020
Li, Chihua: 4115
Li, Chunxiao: 2040, 2500, 7080
Li, Dan: 7430
Li, Daniel: 7285
Li, Donna: 7315
Li, Fan: 3370
Li, Han: 7075
Li, Hong: 7450, 7630, 7930
Li, Hongyu: 1355
Li, Huiling: 9030
Li, Jiajia: 8010, 8085
Li, Jianwen: 8020
Li, Jiaying: 8015
Li, Jing: 3145
Li, Jingxi: 3090, 7510, 7705
Li, Jingyi: 2125
Li, Joyce: 3520
Li, Juan: 2465, 7630
Li, Judy: 7085
Li, Junxin: 1535, 2465, 3335, 4010, 7525, 7695, 8015
Li, Ke: 1290, 3440, 7030
Li, Lingsheng: 4045
Li, Linyan: 8035
Li, Lun: 1100, 4445, 7445, 7740, 8010, 8045
Li, Lydia: 1105, 2260, 3440
Li, Meiyi: 2030
Li, Melody: 8015
Li, Mengchi: 2465, 3335, 4010
Li, Mengting: 8050
Li, Mingyang: 7455
Li, Na: 7275, 7710
Li, Niying: 2065, 7200
Li, Pingnan: 8010
Li, Polly Wai Chi: 7005, 7040
Li, Qi: 1245
Li, Qianyuan: 1235, 2255, 2465
Li, Qing: 7725
Li, Ruixi: 4310
Li, Shuang: 7400, 7920, 8015
Li, Shuwen: 7265
Li, Tao: 7080
Li, Tianyuan: 2030, 3480, 4065, 7610
Li, Wei: 7285
Li, Wenjun: 3245, 7260, 7695, 7935, 8010
Li, Xiaoang: 7320
Li, Xiaoli: 7200, 7425, 7910
Li, Xiaoqian: 7455, 7925
Li, Xiaoyouxiang: 8020
Li, Xinyue: 8020
Li, Xiuli: 7425, 7930
Li, Xuena: 7080
Li, Yaming: 4195
Li, Yan: 3540
Li, Yaxin: 7080
Li, Yiang: 3410
Li, Yichao: 7230
Li, Ying-Chun: 7110, 7400, 7490
Li, Yingru: 8015
Li, Yixia: 3010
Li, Yongmei: 8010
Li, Yu S: 3355
Li, Yuanbin: 7440
Li, Yuanyuan: 3270
Li, Yuekang: 7535
Li, Yuelin: 7275, 7640
Li, Yun: 1090
Li, Zhijiang: 7630
Li, Zhuochun: 1355
Li, Ziyi: 8000
Liang, Bo: 8010
Liang, ChangPu: 7735
Liang, Jersey: 7265
Liang, Jiaming: 1335
Liang, Jianling: 7615
Liang, Nana: 7670
Liang, Peichao: 3320
Liang, Shiwei: 7320
Liang, Wukaiyang: 7075
Liang, Yanjing: 2125, 7415, 7610, 7900
Liao, Gerald Y: 7075
Liao, Hsiao-Wen: 8000
Liao, Jinhui: 4005, 7275
Liao, Yanru: 8020
Liao, Yo-Jen K: 7040, 7200, 8015, 9015
Liao, Zih-Yong: 4110, 7655
Liberalesso Neri, Anita: 8015
Lichtenberg, Peter A: 2155, 3270, 7630, 8000
Liebegott, Ryan K: 8010
Lieber, Sarah B: 7080
Liebman, Michael: 3550
Liebzeit, Daniel: 8020
Lien, Zoe H: 8010
Lifshitz, Rinat: 3530
Light, Michael: 7215
Lighthall, Nichole R: 4420, 7640, 8000, 8015
Liljas, Anne: 7215
Lill, Christina: 7210
Lim, Aaron C: 7495, 7930
Lim, Angelica: 2410, 2530
Lim, Arum: 7440, 7500
Lim, Celestine: 8015
Lim, Emily: 8015
Lim, In-Taek: 8010
Lim, Jun Pei: 8015
Lim, Junyub: 8000
Lim, Kelvin O: 1485, 7145
Lim, Kian Lee: 7150
Lim, Mihye: 7095
Lim, Nahyun: 8000, 8010, 8015
Lim, Wee Shiong: 8015
Lim, Yejin: 4070
Lim, Youn-Hee: 3125
Limaye, Seema: 7250
Lim-Soh, Jeremy: 1255, 2065, 3025, 3545
Lin, Chia Jou: 7040
Lin, Ching-Huang: 7275
Lin, Feng Vankee L: 1135, 7015
Lin, Feng-Chang: 3585, 7535
Lin, Frank R: 1055, 1440, 2525
Lin, Gloria J: 4390
Lin, Haiqun: 1380, 4485
Lin, Hongjing: 7330
Lin, Hui-Yi: 8020
Lin, I-Fen: 4050
Lin, Kueiyu Joshua: 3135
Lin, Ming-Jen: 7510
Lin, Qing: 7000
Lin, Rong: 7450, 7930
Lin, Rose: 9010
Lin, Shih-Yin: 7645, 8020
Lin, Siyang: 7275, 7710
Lin, Vankee: 7450
Lin, Vivian: 8015
Lin, Xin Yao: 2355, 3415, 4420, 7360
Lin, Yen: 7125
Lin, Yi-Hsiu: 7280, 7335
Lin, Yunann: 8000
Lin, Zhao Xiang: 7025
Lin, Zhixuan: 9035
Lin, Zhiyong: 7145
Lin, Zhuoer: 2120, 3165, 7055, 7465, 7925, 8055
Linares, Jackeline: 3190
Lincoln, Karen D: 1060, 4010
Lindauer, Allison: 1125, 3045, 3285, 7425
Lindberg, Brian W: 2245, 2390, 3260, 3390, 4395
Lindberg, Jimmy: 8015
Lindeman, Cliff: 8010
Linden, Wolfgang: 8020
Lindgren, Aleksandra L: 3275
Lindquist, Lee: 4480, 7005, 7420, 7490
Lindsay, Rebecca: 7030
Lindsay, Ryan: 7490
Ling, Shari M: 4230, 8030
Linge, Jennifer: 4310
Lingham, Jayanthi O: 3550
Lingler, Jennifer: 7200
Link, Benjamin: 2200
Linton, Elizabeth: 8000
Lipat, Ania: 1445
Lipsitz, Lewis: 2040, 2080, 2350, 4535, 7450
Lipson, Debra J: 1315
Lipton, Richard B: 2160, 7225, 7455
Liskovec, Leona: 9030
Liston, Dorion: 7715

Lithgow, Gordon: 7010
Little, Kevin: 1490
Little, Natalie: 7300
Littleton, Tenesha: 7535
Litzelman, Kristin: 4095, 7610
Liu, Bingyang: 7315
Liu, Bochu: 4430
Liu, Chang: 7620, 7655, 7720, 8015
Liu, Chiung-ju (CJ): 7205
Liu, Chun: 2080, 2235
Liu, Chunyu: 1255, 7360, 8015
Liu, Fangyu: 8015
Liu, Guangtian: 7280
Liu, Huan: 7510, 7915
Liu, Huangqin: 7670
Liu, Hui: 1110, 3410, 4455
Liu, Jingwen: 7030
Liu, Jingyun: 7275
Liu, Jinming: 7470
Liu, Jinxiu: 7425, 7930
Liu, Jinyu: 1105, 4030, 7055
Liu, Jue: 7350
Liu, Kehan: 2040, 2500, 4055
Liu, Li: 1355
Liu, Lili: 7620, 7630, 7905
Liu, Liyun: 2120
Liu, Longjian: 7620
Liu, Mengjing: 3415
Liu, Mengyu: 8010
Liu, Michelle: 1535
Liu, Min: 3050, 7680
Liu, Minhui: 1235, 1365, 2040, 2255, 2465, 2500, 2530, 3330, 4055, 7080, 7155, 7440
Liu, Pi Ju: 1200
Liu, Qian: 1355
Liu, Qianqian: 4310
Liu, Qimin: 3165
Liu, Rui: 8010
Liu, Ruotong: 7270
Liu, Sofia: 1535, 2465, 3335
Liu, Tao: 7430
Liu, Tianyin: 7640
Liu, Wen: 2550, 7005, 7205, 7245
Liu, Xiaohui: 7670
Liu, Xiaoye: 2030, 3480
Liu, Xiaoyu: 7200, 7425, 7910
Liu, Xinran: 7300
Liu, Xueying: 1245
Liu, Yaqian: 7680
Liu, Yating: 3330
Liu, Yi: 7655, 8015
Liu, Yichun: 3255
Liu, Yifan: 1535, 3570
Liu, Yin: 2065, 7005, 7030, 8020
Liu, Yuanyuan: 7035, 7265, 7520
Liu, Yuchen: 7275, 7280
Liu, Yujin: 1355
Liu, Yuqi: 3200
Liu, Zeyu: 2300
Liu, Zhilin: 4430
Liu, Zhiwei: 3330
Liu, Zhong-Xu: 7455
Liu, Zuyun: 8015, 8055
Liu Woronko, Susan: 4320
Liu-Ambrose, Teresa: 1150, 1365, 1535, 2510, 3335, 7030, 7455, 7720, 8015
Livingston, Melvin: 4355
Livingston, Rachel: 8020
Livingston, Raechel: 1525, 8000, 8020
Lkhamsuren, Zuchi: 1140
Llaneza, Danielle H: 7360
Lloyd, Althea: 3110
Lloyd, Amanda J: 8015
Lloyd, Patricia: 8015
Lo, On-Yee (Amy): 4215
Lo, Temmy L. T.: 7725
Lo-Ciganic, Wei-Hsuan: 2145
Löckenhoff, Corinna: 8020
Locklear, Jessica: 8015
Locklear, Keenya: 8020
Lockman, Jeffrey: 7690
Lodha, Neha: 7630
Loeckenhoff, Corinna: 7690
Loerzel, Victoria: 4420, 7260
Loewenstein, David: 8010
Loewenthal, Julia: 3150
Logan, David: 3455
Logue Cook, Rachel N: 1175, 3085, 7205, 7610
Loh, Kah Poh: 7420, 7920
Lohman, Matthew C: 1535, 7315, 7425, 7930
Lohmar, Sabine: 7115, 7220, 7490
Lomeli, Sindy: 7725, 8010
London, Andrew S: 2385
Long, Chengxu: 1475
Long, Erin: 3565
Long, Monica: 2340, 3175, 7250
Longley, Hannah: 7680
Loomer, Lacey: 7295
Loosen, Julia: 2085
Lopes, Mario: 8010, 8015
Lopes De Oliveira, Thais: 8015
Lopez, Bruna: 2555, 4015, 4140
Lopez, Janet: 8010, 8015
Lopez, Katherine I: 1480
Lopez, Maria J: 4485
Lopez, Ruth Palan: 4045, 8015
Lopez-Ortega, Mariana: 3030
López Anuarbe, Mónika: 1045
LoPilato, Danielle M: 3245, 7695, 7935
Loranger, Elisabeth: 4490
Lord, Justin: 1550, 3255
Loskot, Taylor: 4120, 4180
Lotufo, Paulo A: 1320
Lou, Christine Wei-Mien: 7030
Lou, Qian: 7655, 7720
Lou, Vivian Weiqun: 1115, 4115, 7080, 7150, 7235, 7290, 7640, 7695, 7750, 8020, 8040
Lou, Yifan: 1105, 2305, 4030, 4505, 7055, 7245
Louis-Charles, Willencia: 8000, 8010, 8020
Lourie, Michael: 7470
Louwagie, Katie W: 3130
Lovasi, Gina S: 3375
Love, Jill W: 7325
Love, Madeleine: 7720
Lowers, Jane: 1200, 4395
Lox, Dennis M: 7410
Lu, Chuan: 7655
Lü, Jiaojiao: 4535
Lu, Jiaying: 8000
Lu, Jiehua: 8000
Lu, Lingyi: 7015
Lu, Minjie: 7355
Lu, Peiyi: 4115, 7035, 7290
Lu, Shiyu: 3200, 8000, 8010, 8020, 8035
Lu, Siyao: 4505, 7485
Lu, Xiaomin: 2495
Lu, Yi: 7690
Lu, Yifei: 1000
Lu, Yu: 7660
Lu, Yu-Lan: 7280
Lu, Zhiyong: 1355
Lubben, Jim: 1225, 2055, 4190
Luborsky, Mark R: 7440
Lucas, Iaah: 8015
Lucas, Ruth: 8015
Lucca, Kaitlyn: 1125, 7220
Lucero, Louis: 8020
Lucero, Robert J: 8015
Luchetti, Martina: 7245, 7350
Luchsinger, José: 3180
Luci, Katherine S: 4060, 7695, 8015
Luehrsen, Samantha: 8010
Lui, Li-Yung: 4005
Luiz de Melo, Mariana: 7225, 7655
Lujintanon, Sita: 7540
Luke, Miles: 9040
Luk-Jones, Susanna: 1065
Lum, Hillary: 4215
Lum, Terry Yat Sang: 3200, 8010
Luna, Catherine: 2360
Lunardini, Melissa: 2410
Lunetta, Kathryn: 2160, 7735, 8010
Lunger, Judith: 2310
Luo, Hao: 1455
Luo, Huabin: 3305
Luo, Jing: 1570
Luo, Liying: 2120, 7350
Luo, Minxia: 4185
Luo, Wei: 7430
Luo, Yanan: 4065
Luo, Yuqian: 1365, 2530, 7155
Luo, Yuting: 7630
Luo, Zhehui: 2525, 7090, 7630
Luo, Zhimeng: 1355, 2355
Luong, Gloria: 7255
Lusardi, Michelle M: 7330
Lusignani, Maura: 7245
Luskey, Valerie E: 7070, 7455, 7610
Luth, Elizabeth A: 3220, 3500, 4360, 4550, 8035
Luther, Stephen: 2145
Lutsey, Pamela: 4440
Luu, Britney: 8010, 8015
Luxton, India: 3515
Luy, Luis: 2295
Luz, Helena: 7465
Luzum, Geske: 7060
Lv, Yalin: 2465, 7630
Lwere, Kamada: 1190
Ly, Timothy K: 1325
Lydon, Elizabeth A: 4270, 7015, 7630, 7660, 8020
Lynch, David H: 3585, 7275, 7535
Lynch, Doug: 8015
Lynch, Erin: 9040
Lynch, Morgan: 3220, 7725
Lynch, Scott M: 7625
Lyndes, Kathryn: 8020
Lynn, Freda: 2185
Lyons, Jason: 2085
Lyons, Karen S: 2555, 3285, 4165, 7020, 7245
Lyons, Sophia: 7005

Lyu, Benshuai: 7430
Lyu, Jiyoung: 7275, 7460

M
Ma, Chenjuan: 4360
Ma, Flora: 1325
Ma, Guoyong: 3270
Ma, Hao: 7460, 7715
Ma, Jessica E: 2180
Ma, Jiahui: 7330
MA, Ke: 8010
Ma, Kris (Pui Kwan): 7490, 7935
Ma, Lina: 3085, 3350, 7205
Ma, Mengyao: 7510
Ma, Mingyue: 7690
Ma, Stefan: 3290
Ma, Xinlin: 4430
Ma, Yan: 7060
Ma, Yu: 7515
Ma, Zhihao: 8020
Mülleder, Michael: 7210
MacArthur, Rodger: 7285
Macchia, Lucía: 4440
Macdonald, Birthe: 4525
MacDonald, Elizabeth: 7695
MacDonald, Geoff: 1215
MacDonald, Stuart WS: 7260, 7525
Mace, Ryan A: 2315, 3250
MacGillivray, Thomas: 7425, 7930
Mach, John: 3520
Machinga-Asaolu, Rujeko O: 3275, 7405
Mack, David L: 7075
Mack, Melanie: 4490
MacKay-Dunn, Marion: 7750
MacLeod, Tim: 7325
Macnaughton, Eric: 7040
MacNeil, Andie: 4365, 7025, 7405
MacPherson, Andrew: 2390, 3260
MacTavish, Erica: 7055
Madden, Kenneth: 1365, 7535, 7720, 8020
Madhavan, Sidharth: 4170
Madrigal, Caroline B: 2550, 3240, 8075
Maeda, Harumi: 1480
Magaziner, Jay: 1275, 1410, 7010
Magder, Laurence S: 8010
Magid, Kate: 4460, 8010
Magnani, Jared W: 7330
Magnotta, Vincent: 7735
Magrath, Priscilla A: 8020
Magrini, Mitchel: 8060
Mahajan, Bhushan: 7360
Mahanna, Allexis: 7480
Mahapatra, Gargi: 2235, 7010, 7425
Maher, Brion: 4005
Mahmood, Atiya: 2395, 2560, 4320, 8000
Mahmoud, Kafayat: 4000, 4330
Mahmoudi, Elham: 1495, 8075
Mahmoudi, Parsa: 7640
Mahoney, Jane E: 2155
Mahoney, Jeannette R: 2525
Mai, An: 7200
Mai, Chunyan: 7610
Maiden, Robert J: 2250, 3495
Mailhot, Tanya: 7645
Maillard, Pauline: 4430
Mair, Christina F: 3375
Mair, Christine A: 4050, 4190, 8010, 8020
Majón Valpuesta, Dolores: 1100, 4270
Majid, Tabassum: 3340
Major, Chloe': 7000
Mak, Jonathan K. L.: 7010
Mak, Wingyun: 8010
Makaroun, Lena K: 4195, 8015
Makeen, Mutasim: 4530
Makowski, Maryam L: 1135
Malatyali, Ayse: 7200, 7260
Malcolm, Paulina M: 7040
Malesevic, Nebojsa: 7155
Malheiro, Grace E M: 1070
Malhotra, Rahul: 1095, 1255, 2065, 3025, 3290, 7205, 7300, 8015
Mallender, William: 7750
Mallett, Christopher A: 7040
Malone, Carolyn A: 2305
Maltsev, Alexander V: 8000
Mameno, Tomoaki: 7105
Mami, Gwendolyn: 8020
Mamo, Sara K: 7140
Mandrekar, Jay M: 4415
Manduca-Barone, Alessandro: 4445
Mandujano, Melissa: 2520
Manheim, Chelsea: 8015, 9025, 9040
Mani, Sneha Sarah: 4380, 9035
Manini, Todd M: 1445, 8015
Manly, Jennifer J: 4375, 7350
Mann, Catherine M: 7055
Mann, Jim: 2410, 2530, 7465, 7750, 7910
Manna, Martina: 7275
Mannen, Delia: 7005
Mannheim, Ittay: 3315, 3425, 3530
Manning, John: 8000
Manning, Lydia K: 3450
Manning, Mark: 1495, 9000
Manning, Wendy D: 3065
Manocha, Gunjan: 8020
Manor, Brad: 2040, 4215, 4535
Mansfield, Tyler: 1090
Mansion, Natalie: 4205
Mansoor, Marrium: 7615
Mansour, Amer: 1425
Manternach, Celine M: 7265, 7460
Mao, Hui-Fen: 8010
Mao, Jieyu: 8055
Mao, Junyan: 1355
Mao, Weiyu: 3305, 3505, 8010
Mao, Ziling: 7060
Mar, Amanda B: 7005
Maraganore, Demetrius M: 7425, 7930
Maras, Jessica C: 7255
Marcel, Solange M: 7600
Marchiano, Joseph S: 7440
Marcinek, David J: 1240, 7075, 7210, 7625
Marcus, Michael S: 4545
Marcus, Robin L: 1275
Marcus, Steven: 8055
Mardini, Mamoun: 1445
Marengoni, Alessandra: 7060
Marfeo, Elizabeth: 7135, 7490
Margot-Cattin, Isabel: 1555
Margrett, Jennifer: 8020
Margulis, Brianna: 8010
Marie, Damien: 4490
Mariko, Hawa: 1060
Marin, Omar A: 7215
Marini, Christina: 2385, 7070
Marino, Francesca R: 8015
Marino, Victoria R: 4375
Mark, Katrina: 7060
Markaki, Adelais: 8000
Markides, Kyriakos: 1495, 4070, 7035
Markot, Michael: 3335
Marks, Suzette: 7040
Markwardt, Sheila: 8010
Marnfeldt, Kelly A: 7520
Marottoli, Richard: 7085, 7410, 7900
Marquez, Stephanie Lyn: 8020
Marriott, Deanna J: 7435
Marron, Megan M: 7710, 8000
Marrone, Nicole: 1440
Marrone-Lloyd, Kenzi: 7000
Marrs, Sarah: 1145, 1270, 3510
Mars, Lori: 2001
Marshall, Barbara: 3315
Marshall, Shawn: 3185
Marsiske, Michael: 4490
Martens, Lindsey: 8020
Martens, Nancy L: 8020
Martey, Ebenezer: 8010
Martignetti, Lucas: 3010, 8010
Martikainen, Aleksiina M E: 2365
Martin, Daniel: 2085
Martin, Diane: 8015
Martin, Jennifer: 7660, 7735, 8000
Martin, Jessica: 4060
Martin, Melanie: 8020
Martin, Mike: 1220
Martin, Peter: 1385, 2255, 4095, 7115, 7355, 7610, 8000, 9045
Martin, Summer: 3465
Martin Ginis, Kathleen: 2525
Martinchek, Michelle: 2345
Martindale-Adams, Jennifer L: 4060
Martinez, Adrian: 7210
Martinez, Angelica: 3175
Martinez, Ariza: 8015
Martinez, Daniel E: 8010
Martinez, Erica: 7205
Martinez, Leah: 8000
Martinez, Leilani S: 2520
Martinez, Margarita: 4165, 8000, 8020
Martinez, Nicolina: 8015
Martinez, Suzanna: 8025
Martinez, Vanessa: 1185
Martinez Amezcua, Pablo: 2035, 2525, 7540
Martinez Lujan, Lazaro: 7720
Martinos, Fadi: 3115
Martins-Klein, Bruna: 7255
Martire, Lynn: 4405, 7145, 7455
Marts Skoglund, Eva: 7010
Maruki-Uchida, Hiroko: 8015
Marx, Hannah: 1020

Marx, Katherine A: 4130, 7005, 7465, 7685
Marx, Ruth: 8000
Mas, Rafael J: 7080
Masaki, Harue: 7400, 7505, 7910, 7920
Masaki, Kamal: 1385, 8000
Masarwa, Malek: 7265
Masdeu, Joseph: 2235
Masella, Norine: 7055
Masliah, Eliezer: 1340
Maslow, Katie: 1030
Mason, Hannah: 1165, 7065
Mason, Stephanie: 7420
Mason, Tyara R: 7080
Masotti, Barbara: 1220
Massart, Mylynda: 3045
Massimo, Lauren: 8020
Mast, Benjamin: 7660
Mastel-Smith, Beth A: 4240
Master, Lindsay: 1250
Masternak, Michal M: 8000
Masters, Julie L: 2490, 8000
Masui, Yukie: 2470, 7115, 8020
Matai, Lavina: 8010
Matarazzo, Richelle: 8010
Mateo-Abad, Maider: 4045
Mateos, Jose T: 4255
Mather, Harriet: 4460
Mather, Mara: 7225
Mathershed, Amor: 8020
Mathias, Vincent F.: 4270, 7660
Mathis, Lindsey M: 1075, 4385, 8010, 8065
Mathis, Michael: 1435
Matlock, Daniel: 8020
Matos-Moreno, Amilcar: 2475, 4050
Matsumoto, Yoshiko: 1480
Matsunaga, Hiroko: 7695
Matsuzaki, Yuriko: 7680
Matta Gallero, Constanza Tamara: 7635
Mattam, Ashwin: 8010
Mattek, Nora: 3140
Matters, Doreen: 4450
Matthews, Alicia: 7080
Matthews, Ian: 8000
Mattie, Johanne: 7155
Matz, Christina: 2555, 4140
Mau, Theresa: 2080
Mauck, Erin: 8020
Mauldin, Rebecca L: 1105, 2260, 4345, 7725, 8010
Maulod, Ad: 1015, 2065, 2185
Maupin, Danny: 2465
Mausbach, Brent: 1335, 7660
Maust, Donovan: 7490, 8010, 8055
Maxfield, Molly: 3015, 4010, 4320, 8020
Maxwell, Cathy A: 1265, 7075, 7155, 7435
Maxwell, Hillary: 3185
May, Jennifer T: 7100
May, Terri M: 7205
May, Jr, Alfred J: 3100
Mayahara, Masako: 2370
Mayeda, Elizabeth Rose: 1545, 4035, 4375, 4380, 7535
Mayer, Jamie: 8010
Maynard, Quentin: 7055
Mays, Allison: 2520
Maza, Valentina: 9030
Mazzola, Paolo: 7275
Mbam, Kingsley C: 7070, 7490
Mbamba, Crispin R: 7335
Mbanefo, Onyinye: 8010
McAdams DeMarco, Mara: 2010, 3150, 4420
McAllum, Kirstie: 7635
McAndrews, Carolyn: 7160
McAtee, Robin E: 7325, 7650
McCabe, Janet: 7295
McCall, Jennifer R.: 8015
McCart, Elizabeth: 7210
McCarthy, Ellen: 3135, 7080
McCarthy, Kate: 1265
McCarthy, Megan: 1165
McCarthy, Michael J: 1525, 4165, 8000, 8020
McCauley, Kathleen: 3300, 7535, 8010
McClay, Joseph L: 7715
McClean, Melissa: 1080
McCleary, Lynn: 4175, 7055
McClellan, Angela: 2205, 3320, 7335, 7740
McClure, Jennifer B: 1265, 9020
McClure, Leslie A: 4340
McConnell, Eleanor S: 2000, 2205
McConnell, Kelly: 2295
McConnell, William R: 3190
McCormack, Brendan: 7215
McCormack, Mary E: 1490
McCorvey, Stephanie: 8010
McCoy, Megan C: 1525, 3080, 4165, 8000, 8020
McCoy, Tessa: 7005
McCoy, Thomas: 7275
McCrae, Christina: 1135, 1405, 3055, 3360, 7500
McCreedy, Ellen: 1115, 1530, 3240, 8010, 8015
McCrory, Cathal: 8060
McCullough, Louise D: 3470
McCullough, Megan: 7685
McCune, Teagan: 7635
McCurry, Susan M: 7735, 8010
McDaniel, Kennedy: 2010, 4420
McDaniel, Laura E: 8000, 8015
McDarby, Meghan: 7055
McDermott, Cara: 7470
McDermott, Kate: 2315
McDevitt, Ross A: 7210
McDonald, Claire: 7065
McDonald, Margaret V: 1395, 3105, 4360
McDonald, Shelley R: 8015
McDonnell, Jenny L: 7530
McDonough, Christine M: 1275
McDonough, John: 4170
McDonough, Meghan H: 3465
McDowell, Cynthia: 7525
McElfish, Pearl: 8000, 8015
McElroy, Jane A: 7100
McEvoy, Sarah: 8020
McFadden, Angela: 8015
McFarland, Malcolm: 2300
McGarry, Kathleen: 3145
McGee, Blake T: 1230
McGee Lawrence, Meghan: 7285
McGhan, Gwen: 3465
McGill, Robert M: 9020
McGilton, Katherine: 4255, 8015
McGinity, Ashley C: 8010, 8015
McGinley, Jacqueline: 7410
McGinty, Patrick: 3585
McGough, Ellen: 7655
McGovern, Justine: 4540
McGovney, Kevin: 1405, 3360, 7500
McGowan, Joseph: 8010
McGrath, Lydia: 8015
McGrath, Ryan: 1525
McGuire, Katrina: 7720
McGuire, Lisa: 3100, 4235, 4390, 7200, 7205
McHale, Jenna R: 7465
McHugh, Molly: 8010
McIntosh, Nathalie: 1080
McIntyre, Madeline: 1145, 3510, 8000, 8010
McIntyre, Thomas: 7630
McKay, Sandra: 8015
McKay, Tara: 1170, 3075, 8080, 9030
McKelvey, Miechelle: 2205
McKenna, Kevin: 2090
McKenzie, Colin: 7130
McKenzie, Michael R: 7465
McKibbin, Christine: 7440, 7490
McKiddy, Sarah: 2115, 2450, 4285, 7415, 7900
McKinley, Catherine: 4110
McKinnon, Monette L: 3215
McKneely, Katelyn: 7045
McLaren, Jaye: 3395, 9020
McLaughlin, Sara: 1105
McLaughlin, Sara J: 8010
McLean, Elisabeth: 1335, 3385, 4435, 7425, 9015
McLin, Maia: 7610
McMullen, Tara: 1315, 3090, 8030
McMurray, Josephine J: 3315
McNally, James: 8090
McNamara, Kelsey C: 8000
McNeish, Brendan: 2220
McNellis, Cynthia: 8010
McNicoll, Lynn: 7070
McPeak, Ashley: 4470
McPherson, Rachel: 4090
McPhillips, Emily A: 8015
McPhillips, Miranda V: 1535, 3335, 7525
McVey, Ava: 1185, 8000
Meadows, Jeffrey: 8020
Mealer, Meredith L: 7155
Meara, Ellen R: 7000
Mechanic-Hamilton, Dawn: 8020
Medcalf, Robyn: 7490
Medders, Laura: 7220, 8015
Medina, David: 8000
Medina, Jose T: 3035
Medlin, Austin: 1455, 2085
Medrano, Valeria: 8015
Meek, Paula M: 7440
Meghelli, Ilan: 8010, 8020
Mehboob, Gul Rukh: 8075
Mehl, Matthias: 8020
Mehrabi, Fereshteh: 1225, 2230
Mehrjerd, Ameneh: 7010
Mehta, Neil K: 4455, 7230, 7300, 8000
Mehta, Rhea: 1535, 3035
Mei, Yongxia: 3330
Meier, Diane: 2520

Meier, Helen CS: 1495, 8060, 9000
Meijers, Judith MM: 4255, 8015
Meinertz, Naomi R: 1165, 3055, 3575
Mejia, Shannon T: 1570, 4420, 7720, 8000, 8020, 9050
Mejia Guevara, Ivan: 4035
Melero-Dominguez, Maria: 4130, 7660
Melgar Castilo, Andrea I: 8065
Mell, Johnathan: 7490
Mellett, Katlyn: 8010
Melo, Rosa: 7465
Melo, Ruth C: 4465, 8015
Memmer, Nicole: 1040
Mena, Emily: 4345
Menard, Alixe: 1010
Menashe, Aviad: 7600
Mendes de Leon, Carlos F: 1155, 3540, 8020
Mendez, Benjamin: 3150
Mendez, Jennifer: 1050
Mendez, Therese M: 7315
Mendez Campos, Barbara: 2555, 3275
Mendoza, Edna Patricia: 9050
Meneilly, Graydon: 7205
Meng, Hongdao: 2370, 3365, 7455
Meng, Lingsong: 1445
Menne, Heather L: 2070, 2495, 3565, 7145, 7295
Menne, Heather: 8010
Mensik, Ashley: 8020
Mentch, Heather: 2480
Mera, Alejandra L: 7215
Mercan, Murat A: 7160
Mergenthaler, Andreas: 1100
Mernitz, Sara: 1540
Merrilees, Jennifer: 3285
Merrill, David A: 7425, 7930
Merrill, Ray M: 8015
Merss, Kristin: 7200
Merten, Natascha: 1000, 4300
Mertz, Kathryn: 7695, 7695
Mertz, Laurel: 7050, 7085
Metcalf, Judith: 8015
Meyer, Ilan H: 1110
Meyer, Kylie: 2375
Meyer, Oanh: 4430, 8025
Meyers, Gabrielle: 7020
Meynadasy, Melissa: 8020
Meynet, Stéphanie: 1100
Meza, Erika: 1495
Mezuk, Briana: 3235, 3540, 7490, 7695
Mgabhi, Philile: 7150, 7735
Mi, Hong: 2185, 3270, 7230, 7295, 7510, 7740, 7915, 8010
Miao, Hongyu: 2240
Miceli, Chelsea: 7340
Michael, Yvonne L: 8020
Michaels, LeAnn: 4160
Michaud, Veronique: 7425, 7630
Micheel, Frank: 1100
Michel Pinto, Antonio F: 7425
Michelson, Lisa: 7275
Mick, Paul: 4300, 7635
Mickus, Maureen A: 7510
Middleton, Laura E: 8015
Miglani, Jasroop K: 8000
Migliaccio, John N: 4545
Migneault, Deborah: 7740
Migovich, Miroslava: 7155
Miguel-Cruz, Antonio: 7620, 7630, 7905
Mijkovic, Iva: 2220
Mikal, Jude P: 1485, 7145
Mike, Leigh Ann: 4470
Mikels, Joseph A: 3355
Milan, Rebecca A: 7420
Milani, Sadaf A: 1495, 4095, 7025, 7235
Milberg, William: 3335
Mildrum Chana, Sofia: 7700
Milidonis, Mary: 3585
Miljkovic, Iva: 2220, 7010, 8000
Millar, Courtney: 2080, 7450, 8000
Miller, Edward A: 1360, 3495, 4305, 7295, 7670
Miller, Emily: 7700, 7935
Miller, Jacqueline: 2265
Miller, Julie: 1370, 2001, 2420, 3145, 4140
Miller, Justin B: 8055
Miller, Katherine: 1170, 1395, 2500, 3570, 4520, 7005, 7470
Miller, Kenneth L: 7330
Miller, Kristin A: 3575
Miller, Lindsay: 3330
Miller, Lyndsey M: 3285, 4160, 4165
Miller, Maggi: 1045, 7425, 7930
Miller, Mary Beth: 1405, 7500
Miller, Matthew J: 3010
Miller, Matthew: 4430
Miller, Melicent: 4510
Miller, Michael E: 1090, 1410, 2220, 2505
Miller, Nancy: 7515
Miller, Richard: 8000
Miller, Sara E: 7675
Miller, Sarah J: 7115
Miller, Steven J: 1530
Miller, Vivian J: 7505
Miller-Winder, Amber: 7005, 7420
Mills, Sarah: 2090
Mills, Whitney L: 1080, 3425, 7250, 7545, 8020
Milnamow, Mary: 2265, 7155, 8025
Mimbs, Hope: 1010, 7160
Min, Hae Youn: 4505, 7070
Minard, Charles G: 1420, 2080, 2235
Minch, Kendra: 8010
Mindlis, Irina: 7270, 7630, 8020
Miner, Jennifer: 7065
Mingo, Chivon A: 2245
Minor, Bryan J: 2360
Mintz, Yonatan: 7510
Minyo, Morgan J: 1030, 1265
Mion, Lorraine C: 7435
Mir, Nabiel A: 7205
Miranda, Jacob F: 7685
Miran-Khan, Karim: 1365
Mirarchi, Kelly A: 7150
Mireles, Josh: 8050
Mirjan, Nadia: 7200
Mirza, Mansha: 8015
Mirza, Noeman: 4025, 8010
Mirza, Raza M: 7480, 7900
Mishra, Sidharth P: 7210
Misonow, Julia: 4345
Missell-Gray, Rachel: 4400
Mitchell, Barbara A: 4445, 7445, 7740
Mitchell, Calli O: 7680
Mitchell, Colter: 8060
Mitchell, Jacob: 7235
Mitchell, Jamie: 3170
Mitchell, Lauren L: 3130
Mitchell, Mary: 1470
Mitchell, Michael N: 7735, 8015
Mitchell, Uchechi A: 3180
Mitschke, Diane: 2195
Mitsova, Diana M: 4430
Mittal, Kanika: 2340
Mittelman, Mary S: 1380, 3130
Mitzner, Tracy L: 1040, 3415
Miwa, Hiroyasu: 7750
Mixon, Amanda: 8035
Miyae, Yamakawa: 7330
MIYAMA, Tomoko: 8015
Miyamae, Fumiko: 7350
MIYASHITA, Mariko: 8015
Miyawaki, Christina E: 2205, 3320, 4070, 7335, 7740
Miyazaki, Atsuko: 1205, 8015
Mizell, Nate: 7530
Mkuu, Rahma: 7440, 7935
Mo, Cen: 1365
Moaddel, Ruin: 8000
Mobley, Catherine: 2355
Mobley, Taylor M: 4035, 4375
Mochel, Amy: 7250
Moczygemba, Walter: 2105
Modig, Karin: 7510, 7915
Modrek, Sepideh: 4035, 8020
Moeller, Kelly: 1520
Moen, Maria D: 3090
Moffitt, Chad: 3175
Moffitt, Terrie E: 2220
Moghekar, Abhay: 3070
Mogle, Jacqueline: 1080, 1250, 1525, 2160, 4280, 4515, 7255, 7630
Mohamed, Leena: 8015
Mohamed Mansoor, Mishfak Abdullah: 8000
Mohammed, Hajir: 8015
Mohankumar, Rakshitha: 1325
Mohanty, Sanjay: 3335
Mohd Farhan, Nur ‘Atiqah: 2065
Mohile, Supriya: 7420, 7920
Mohr, David C.: 7545
Mohr, lynn L: 3500
Moineddin, Rahim: 7300
Mois, George: 8010, 8020, 8020
Mokuau, Noreen: 4015
Molholm, Sophie: 8000
Molina, Anthony: 1435, 2235, 2435, 4170, 4210, 7010, 7425, 8000
Molina Hermosilla, Irene: 3150
Molinari, Victor: 7355
Molly, Delzio: 3010
Molnar, Lisa J: 3455
Molton, Ivan: 7240
Monaghan-Geernaert, Pamela: 4435
Monane, Mark: 7425, 7930, 8070
Mondol, Swastika: 8020

Mongoven, Jennifer: 7065
Monin, Joan K: 1345, 2075, 2305, 3285, 4165, 7245, 7270, 8010, 8020
Monroe, Kelly: 1275
Monroe, Stephanie: 3030
Monroe-Lord, Lillie: 7030
Montano, Monty: 1330
Montayre, Jed: 7680
Monteiro, Andréa: 7040
Montepare, Joann M: 1310, 2440, 3120, 3310, 3515, 4240, 7415
Montgomery, Aoyjai P: 8000
Montori, Victor M: 8010
Montoro-Rodriguez, Julian: 7265
Montoya, Ana: 8015
Monzio Compagnoni, Matteo: 7275
Moo, Lauren R: 2430, 3110, 7135, 7420, 7490, 8010, 9020
Moody Jones, Beth: 8015
Moon, Chooza: 7735
Moon, Heehyul: 1045, 7425, 7500
Moone, Rajean: 1520, 2225, 2535, 3490
Moonen, Esmee: 8015
Mooney, Aimee: 1125, 7425
Mooney, Laken A: 8000
Mooney, Scott: 7360
Moore, Alison A: 3065, 3115
Moore, Ann Zenobia: 4170, 7720
Moore, Kari A: 8020
Moore, Miranda A: 7220
Moore, Raeanne: 7660
Moore, Ryan C: 8020
Moore, Susan L: 3575
Moored, Kyle D: 1240, 2045, 3375
Moorman, Sara M: 1140, 1280, 2001, 3410, 4400
Moorthy, Arsha: 7075
Mor, Maria: 4195
Mor, Vincent: 3240, 7030
Morales, Joseph: 3515
Morales Hernandez, Beatriz: 4475
Moran, Ryan: 7080
Morante, Mariana: 4290
Moreau, Kerrie: 1410
Moreines, Laura T: 1395
Morello-Frosch, Rachel: 4035
Moreno, Julie A: 7075
Moreno, Monica: 2305, 4200
Moreno, Sylvain: 3060
Morgan, David: 7425
Morgan, Debra G: 7135
Morgan, Jennifer: 4520
Morgan, Jennifer Craft: 4290, 8010, 8020, 8070, 4290
Morgan, Kerri: 7260
Morgan, Robert O: 3240
Morgan, Stephanie: 7435
Morhardt, Darby: 1125, 4480, 7220
Moriarty, Haley: 8015
Moriarty, Helene: 7290
Moriarty, Sheila: 7250
Morin, Brittany: 2020
Morin, Ruth: 3010
Morisaki, Naoko: 7105
Moritz, Robert: 8000
Moriyama, Yoko: 7490
Mork, Paul Jarle: 7005
Morka, Angela: 7085
Moro, Manuel: 4310
Morris, Jada L: 7080
Morris, John C: 4515, 7720, 8015
Morris, Stephen: 4195
Morrison, Christopher D.: 8000
Morrison, Jake: 4325
Morrison, Lindsay: 3465
Morrison, Wanda: 7480
Morriss, Emily P: 1495
Morrow, Isaiah: 7635
Morrow, Jason: 7530
Morrow-Howell, Nancy: 7535, 8000, 8010
Mortenson, Ben: 2410, 2530, 7300
Morton, Cynthia: 3090
Morton, Elizabeth R: 8010
Morton Padovano, Cynthia: 2330
Moser, Debra K: 3320, 7215
Moses, Ellen Sue: 4165
Mosesso, Kelly M: 7625
Mosley, Thomas H: 1000, 4440, 7060
Mosqueda, Laura: 1270, 2001, 2150, 2375, 4355, 7495
Moss, Benjamin: 4170
Moss, Daniel: 1185, 8020
Moss, David: 3285
Moss, Karen O: 2415, 7490, 7935
Moss, Mark: 3305
Motilla, Ronaldo A: 2405
Moura Maurmann, Rafael: 8000
Mourany, Lyla: 7235
Mousavi, Seyedeh Leila: 7210
Moustafa, Sara A: 1155
Mouton, Charles P: 7405
Moxley, Jerad: 7080, 8020
Moy, Marilyn: 7720
Moye, Jennifer: 1325, 2345, 2385, 3425, 8020
Mpofu, Elias: 3085, 8080
Mroczek, Daniel: 1570, 4375, 4480
Mroz, Emily L: 1345, 2305, 7055, 7310
Mroz, Tracy M: 2500, 4100, 4360, 7080, 7545, 8010
Mu, Christina: 3275
Muñoz, Elizabeth: 2330, 7225, 7485, 7925
Mudar, Raksha A: 1405, 3415, 4270, 7015, 7270, 7455, 7630, 7660, 8015, 8020
Mudrazija, Stipica: 3030, 7470
Mueller, Alexandra: 2230
Mueller, Christine: 4125
Mueller, Kim: 3425
Mueller, Megan: 8020
Mueller, Ranell L: 7610
Muench, Ulrike: 8055
Muir, Michael: 7010
Mukherjee, Bhramar: 1435
Mukherjee, Namrata: 8010
Mukherjee, Shalini: 1565
Mukhtar, Yerkyebulan: 1140
Mulcahy, John: 7425
Mulhorn, Kristine A: 2470, 7230, 7680
Mulkey, Heather: 8010
Muller, Chandra: 1095, 7230, 7350
Mulligan, Kate: 8020
Mulligan, Michael: 7625
Mumby, Rachel A: 1115
Mumma, Kara T: 3415
Mun, Kyoungmi: 7430
Muneoka, Shelley: 7055
Mungas, Dan: 7535
Muniz Terrera, Graciela: 4535, 7425, 7535, 7540, 7930
Munly, Kelly A: 7690
Munnell, Alicia H: 2420
Munoz, Elizabeth: 3095
Munoz, Richard: 2545, 8010, 8070
Murabito, Joanne M: 2160, 7360, 7735, 8010, 8015
Murali, Komal Patel: 4360
Muramatsu, Naoko: 8020
Murawski, Alaine: 7005, 7420, 7490
Murayama, Hiroshi: 4190, 7350, 8015
Murillo, Fernando I: 3420
Muroff, Jordana: 7475
Murphey, Yi L: 8025
Murphy, Blake: 3285, 8060
Murphy, Rachel A: 7535, 8020
Murphy, Sara C: 1065, 7080
Murphy, Terrence: 7040
Murray, Andrew: 1160
Murray, James: 7325
Murray, Sarah: 7665, 8015
Murthy, Venkatesh L: 7710, 8000
Murton, Andrew J: 1130
Musa, Najma A: 7480
Musci, Rashelle: 7615
Musi, Nicolas: 2505, 4210
Musich, Madison: 1135
Musil, Carol: 7265, 7660
Muskin, Ryan: 8020
Muss, Hyman B: 8000
Mussaad, Ahmed: 8015
Mustafa, Dina: 7320
Mutchler, Jan E: 1475, 7485
Muthupillai, Raja: 2080
Mutthina, Ramesh: 7425, 7910
Mwanda, Sheila: 8015
Mwangi, Eunice Muthoni: 4380
Mwendwa, Denee: 8020
Myagmarjav, Sugarmaa: 1140
Myers, Janet A: 2520

### N

Na, Annalisa: 9010
Nabong, Jennifer: 1490
Nabors, Emily B: 1005
Nadarajan, Gayathri Devi D/O: 8015
Nadash, Pamela: 1360, 4115, 7490, 7670
Nadobnik, Halina: 2565
Nadorff, Danielle Kristen: 1360, 3360, 7610
Nagarajan, Niranjani: 4380
Nagel, Corey L.: 3035, 7300, 7700
Naglie, Gary: 3185
Nagururu, Nimesh V: 2080
Nah, Suyoung: 8020
Naidoo, Nirmala: 8015
Naik, Aanand D: 2075, 4340, 7215
Naim, Sigal Pearl: 3465, 4040

Nair, Anil: 8015
Nair, Malini: 8015
Nair, Sangeeta: 2220
Nair, Tripti: 8000
Nair, Uma: 7500
Nakad, Lynn: 1530
Nakagawa, Kazuma: 8000
Nakagawa, Takeshi: 2470, 7115, 7490, 8015, 8020
Nakamura, Masahide: 8015
Nakanishi, Miharu: 2255, 7640
Nam, Stephanie J: 4535
Namasivayam-MacDonald, Ashwini: 2060, 3500
Nambisan, Priya: 7070, 7360, 7425, 7540, 7930
Namrouti, Amina: 7125
Namukasa, Juliet: 8010
Nandigam, Maanasa: 7085
Nanjiba, Nushaiba: 2395
Napoleon, Betty: 1490
Narine, Donnette R: 7350, 7475
Nash, Princess: 3240
Nashef-Hamuda, Reem: 3015, 7130
Nasiri, Rojan: 4320
Nasr, Samer: 7730
Nasrallah, Ilya: 2220
Nathan, Kobi: 8035
Nathaniel, Thomas: 8000
Natsume, Yukie: 7315
Naud, Daniel: 1100, 3320
Navos, Melanie: 7440
Naylor, Mary: 3300, 7435, 7535, 8010
Nazario-Acevedo, Jaminette M: 1505
Nduka, Chinelo: 7600
Neale Furneaux, Heather K: 7040
Nearing, Kathryn A: 1415, 7155, 8020
Nebeker, Johanthan: 8030
Nece, Patricia: 3580
Nederostek, Kate: 3100
Needham, Belinda: 1290, 2045
Neelamegam, Malinee: 2445, 7700, 8000
Ne’eman, Ari: 7000
Neer, Emory: 8010
Neff, Donna F: 2330
Neiberg, Rebecca H: 1090, 1390, 1410
Neiderman, Adrianna C: 7225, 7500
Neidre, Daria: 2295
Neill, Kathryn: 7650
Neilson, Lee: 7310
Neiman, Joseph: 4530
Neises, Kristen: 7220
Neller, Sarah A: 2395, 3385, 7085
Nelson, Christi L: 2540
Nelson, Elizabeth: 3065
Nelson, James F: 3350, 4310
Nelson, Matt: 4510, 7295
Nelson, Niccole A: 3165, 7255
Nelson, Suchitra: 3340
Nelson-Faulconer, Jennifer: 2460
Nelson-Twakor, Elizabeth: 8015
Nemati, Donya: 3050, 3235
Nemeth, Samuel: 8020, 8035
Nemmers, Natasha L: 1335, 2015, 3165, 7065
Nemoto, Kiyotaka: 8015
Nemoto, Miyuki: 8015
Neretti, Nicola: 3020
Neri, Mel: 8025
Nessen, Sarah: 8015
Nesser, Whitney: 7680, 7725
Nesvet, Matthew: 7110
Neubauer, Noelannah: 7630, 7905
Neugebauer, Benjamin: 9015
Neugebauer, Volker: 1335, 3385, 4435, 7065, 7425, 9015
Neupert, Shevaun D: 1565, 4020, 4405, 7610
Newby, Launa: 3140
Newman, Anne: 1240, 1410, 2080, 2220, 2510, 4005, 7060, 7710, 8000
Newman, Anne B: 8020
Newman, David: 7360, 7425, 7610
Newman, John C: 1460, 2530, 3210, 4170, 7075, 8000
Newman-Casey, Paula Anne: 7205
Newmeyer, Natalie: 8015
Newsham, Tina: 2130, 2325, 3265, 3495, 8010
Newsom, Jason T: 3035
Ng, Amy C C: 4390
Ng, Angus Jun Jie: 8015
Ng, Boon Peng: 8015
Ng, Crystal: 8020
Ng, Peter H F: 8020
Ng, Reuben: 1425, 2270, 3015, 7155
Ng, Yee To: 4185
Ngo, Victoria: 3110, 7490
Ngugi, Anthony: 4380
Nguyen, An T: 3300
Nguyen, Andrew H: 9020
Nguyen, Anh Thy H: 7040
Nguyen, Ann W: 1060, 2055, 3045, 4190
Nguyen, Annie L: 3115, 7495, 7930
Nguyen, Annie: 8000
Nguyen, Boi-san: 8025
Nguyen, Duy: 8010
Nguyen, Emerald: 4085
Nguyen, Hoang: 4070
Nguyen, Jade: 7510
Nguyen, Kim N: 2205
Nguyen, Kristine: 7225
Nguyen, Manh Hung: 8020
Nguyen, Nhan: 7145
Nguyen, Thien: 8015
Nguyen, Tho: 7620, 7655, 7720, 8015
Nguyen, Tiffany: 7525
Nguyen, Trang TH: 1100
Nguyen, Uyen-Sa D T: 9050
Nguyen, Victor H: 7085
Nhan, James: 8000
Nhpang, RoiSan: 7700
Ni, Pengsheng: 7420
Ni Leime, Aine: 2425
Nicholas, Lauren H: 3145
Nichols, Emma: 3335, 3540
Nichols, Linda O: 4060
Nichols, Madeline J: 4280, 7730
Nicholson, Jody: 4280
Nicklas, Barbara J: 1240, 1410, 2485, 2505
Nicklett, Emily J: 4475
Nickrent, Megan S: 8020
Nicolay, Sarah: 7470, 8020
Nicosia, Francesca M: 1245, 3010
Nidadavolu, Lolita S: 4005
Nie, Hao: 7075
Niedoba, Simone: 1555
Nielsen, Karen: 1230
Nielsen, Kelli: 7000
Nielsen, Lisbeth: 1340, 4500
Nielsen, Natalia N: 7060
Nieman, Carrie L: 1440, 4300
Niimi, Yukari: 8015
Nikahd, Melica: 2445, 4475
Nikitin, Jana: 1375, 2300, 7245
Nikzad, Amir: 7695
Nikzad-Terhune, Katherina: 3450, 4240, 8010
Ning, Yanhua: 7640
Nishi, Mariko: 7695
Nishida, Yoshifumi: 1505, 7260
Nishikitani, Mariko: 7430
Nishimura, Jun: 7295
Nishita, Christy: 3565, 8010
Nishitani, Megan: 7010
Nishiyama, Hiroyuki: 8015
Nittinger, Sophia: 7440, 7935
Nix, Caitlyn A: 4120
Nizza, Megan: 3090, 7475
Njoku, Crystal: 7150
Nketsiah, Ebow T: 8020
Nkimbeng, Manka: 1465, 2015, 3025, 7640
Nnemnbeng, Jeannys F: 8010
Noble, Cheryl: 3515
Noble, Molly E: 1160, 8010
Nofuji, Yu: 7275, 8015
Noguchi-Watanabe, Maiko: 7295, 7340, 7510
Noh, Hyunjin: 7440
Nolte, Julia: 8020
Nomura, Ayano: 7260
Nomura, Mitsunori: 4170, 7075
Nonaka, Kumiko: 8015
Noor, Mirza Ishrat: 8000
Norberto Pereira, Graziele: 7225, 7655
Norgaard, Martin: 8010
Noriega de la Colina, Adrian: 3050
Noriega-Makarskyy, Daisy: 7495, 7930
Norling, Amani M: 8000
Norman, Kristina: 7315
Norris, Grace: 1070, 2410, 7465, 7910
Norris, Hayden S: 7300
Norris, Keith C: 3475
Norstrand, Lu: 3495
Nortey, Rose T: 7070
Norton, Edward: 3145, 7490
Norton, Sara: 8010
Nothelle, Stephanie: 2085, 4300
Noureddine, Sarah: 8000
Nouri, Mohammadjavad: 3205
Nouryan, Christian: 8010, 8015
Novek, Sheila: 7040
Nowakowski, Alexandra C H: 3075
Nowels, Molly: 7110
Nowicki, Scott: 7060
Nowinski, Cindy: 4230
Noy, Roy: 8010
Noyer, Emily: 7660, 8020
Numata, Keizo: 7105

Nutakor, Jonathan A: 7020
Nwakasi, Candi: 3475, 7600
Nweke, Chizobam: 7600
Nwoke, Mary Basil: 4000
Nyang'au, Getrude K: 3055
Nyarko, Albert Junior: 8015
Nyarko, Veneranda: 8000
Nyrop, Kirsten: 8000

**O**

Obando, Viviana: 8015
Obeid, Jihad: 2355
Oberdier, Matt T: 8015
Obhi, Hardeep: 1385
Obioha, Chidiebere Wisdom: 4000
O'Brien, Charles: 8015
O'Brien, Erin: 8020
O'Brien, Jennifer L: 7610
O'Brien, Kyra: 8015
Obuchi, Shuichi: 7205, 7490, 7540
Ocanas, Sarah R: 4310
O'Connell, Jeremy D: 1500
O'Connell, Megan: 7030
O'Connor, Maureen: 9020
O'Connor, Melissa: 7055, 7150, 7360
O'Conor, Rachel: 4480
Odden, Michelle C: 8010, 8015
O'Dea, Eireann: 3200, 4445, 7155, 7445
Odeyemi, Emmanuel: 4000
Odlum, Michelle: 1465
Odo, Obinna Casmir: 3475
O'Donnel, Arden: 1345
ODonnell, Arden E: 7215
O'Donnell, Caitlin: 7000
O'Donnell, Kari: 7700, 7935, 8000
O'Donnell, Kennedy: 3565
Oduro, Elisha B: 8010, 8015
Offerman, Jens: 7155
Ofori, Edward: 8015
Ofstedal, Mary Beth: 7265, 7525
Ogawa, Elisa F: 3335
Ogawa, Kenichi: 7400, 7920
Ogawa, Madoka: 8020
Ogawa, Susumu: 1265
Ogg, Jordan D: 7320
Ogle, Destiny M: 7070, 7265
O'Grady, Brandon B: 7300
O'Grady, Thomas: 3255
Ogugu, Everlyne: 2200
Ogunade, Temi: 8020
OGUNGBILE, Adedayo J: 8075
Ogunjesa, Babatope: 7145
Oh, Chorong: 9010
Oh, Esther S: 1440, 4005, 4300
Oh, Eunna: 7640
Oh, Gahee: 3135, 7080
Oh, Kyeung Mi: 1165
Oh, Oonjee: 7270
Oh, Patricia: 7415
Oh, Sarah Soyeon: 7630
Oh, Seungjun: 8015
Oh, Yun taek: 1475, 7050, 7130
OHala, Max: 3490
O'Hanlon, Ann M: 7315
Ohene, Eric: 8075
O'Hern, Steve: 3455
Ohgi, Sayuri: 2045
Ohmer, Mary L: 1290
Ohta, Takahisa: 8015
Ojelabi, Olanike: 4355
Ojo, Eunice: 7260
Okada, Jamil: 8015
Okamura, Tsuyoshi: 7350, 7680, 8015
Okechi, Benard Chibuike: 4000
Okereke, Olivia I: 2080, 2315, 3250, 4550
Okine, Joana: 7095
Okolie, Tochukwu J: 2495, 8010
Okoye, Ifeoma J: 8065
Okoye, Safiyyah M: 3275
Okpoebo, Amanda: 8010
Okrah, Albert K: 8015
Okubo, Suguru: 7490
Okun, Sarit: 1190
Okura, Tomohiro: 8015
Oladejo, Dianne: 4480
Oladimeji, Abolade: 8000
Olanrewaju, Sherif A: 7535
Olds, Danielle: 2500
O'Leary, Bryant: 7260
O'Leary, Bryant J: 8020
O'Leary, Meghan: 7125
Olivari, Benjamin S: 4390, 7200, 7205
Oliveira, Iara B: 4305
Olivieri-Mui, Brianne: 3135, 4225, 7080, 8015
Olmo, Jeana: 7035
Olmos, Marina: 7055
Olsen, Bonnie J: 2150, 4355, 8055
Olson, Sarah: 2080
Oltmanns, Thomas: 8010
Olvera, Alfredo: 7400, 7920
Olvera, Alice Gavarrete: 2020
Olvera, Charlie: 7005, 7420, 7490
O'Malley, Kelly: 2370, 2385, 3425, 8020
O'Malley, Pearse: 3115
O'Malley, Terrance: 3090
O'Mathuna, Donal: 8020
Ome, Blessing Nneka: 4000
O'Muircheartaigh, Colm: 1110
Omura, John: 7205
Ondanat Veetil, Shyam Krishnan: 9020
O'Neil, GeVonne C: 4100
O'neil, Jahn: 7000
O'Neil, Kelly: 7420
O'Neill, Margaret: 2425
Ong, Anthony: 1570
Ong, Chong Yau: 8015
Ong, Lawrence: 8015
Ong, Liane: 7040
Ong, Lydia Q: 4405
Ong, Marcus Eng Hock: 8015
Ono, Keiko: 7055, 8010
Onyechi, Chimaoge: 2235
Operario, Don: 8015
Oppong, Kwaku Duah: 2090
Opresko, Patricia: 3020
Orange, Joseph B: 1070, 2410, 7635
Oravecz, Zita: 8020
Orchard, Carole A: 7525
Orewa, Gregory N: 1425, 1550, 3255
Orkaby, Ariela R: 8015
Orkibi, Hod: 8020
Orlovsky, Irina: 7255
Ornago, Alice M: 4315, 7010
Ornstein, Katherine: 2155, 2230, 3570, 4460, 4520, 7470
Orr, Miranda E: 3020
Orr, Timothy: 3020
Orsega-Smith, Elizabeth: 7545
Orsulic-Jeras, Silvia: 7465, 8020
Ortega, Alexandra: 8010
Ortega Hernández, María: 3190
Ortenberg, Elia K: 4085
Ortiz, Hector L: 7495
Ortiz, Jahmar: 2175
Ortolá, Rosario: 3330
Orwig, Denise: 1275, 1535, 2015, 3035, 7010, 8095
Ory, Marcia G: 4130, 4340
Osae-Oppong, Edward: 3115, 7280
Osahon, Ob W: 2235
Osborne, Danielle: 7200
Osborne, Jason W: 7145
Osei, Evans A: 8015
O'Shields, Jay: 7155
Oskarsson, Bjorn: 2200
Osmond, Claudia: 7480, 7900
Osokpo, Onome H: 3300, 7440, 7535, 7925, 8010
Osorio, Jose: 3330
Osorio, Leslie: 7530
Osso, Francesco: 1165
Ost-Mor, Sharon: 8010, 8020
Ostrem, Nicholas: 3485
Ostrom, Ethan L: 7210
Osuka, Yosuke: 8015
O'Sullivan, Kelly: 2295, 7350, 7530
O'Sullivan, Roger: 1225, 1225, 2055
Osuna, Margarita Maria M: 1320
Oswald, Austin G: 2540, 4250, 4445, 4495
Oswald, Frank: 1555, 3155
Ota, Kazuki: 8015
Ota, Miho: 8015
Otake, Eriko: 8015
Otewa, Meshack W: 2115, 3140
Otgon, Saranchuluun: 1140
Otsuki, Naoko: 7080, 7105
Ott, Brian R: 7320
Ott, Corilyn: 3240
Ou, Alison Y.T.: 8050, 2050
Ou, Yinuo: 2465, 7630
Ouellet, Jennifer: 7410, 7900
Ouyang, Meishuo: 3085
Ouyang, Zheng: 4270
Oveisgharan, Shahram: 1235
Overbeck, Kevin: 8010
Owen, Donna C: 7080
Owens, Becky: 4320
Owens, Joshua: 2020
Owens, Victoria: 8010
Owino, Jonix: 2195, 7030
Owsiany, Montgomery T: 7115, 7490
Oxley, Jennie: 3455
Oyales, Carmelina F: 2405
Oyesanya, Tolu: 4530
Oz, Naci: 7210

**P**

Pa, Judy: 7750, 8015
Pärtel Araújo, João: 7465
Paccagnella, Omar: 7035
Pace, Doug: 3535

Pack, Allison: 4480
Padala, Kalpana P: 7360, 7630
Padala, Prasad R: 7360, 7630
Pagan, Jose: 3540
Page, Kyle S: 1080, 7695
Paguirigan, Mark Ryan B: 7205
Pahlen, Shandell: 4095
Pahor, Anja: 8000
Pal, Jay: 7625
Palavicini, Juan Pablo: 1130, 7000
Palena Hall, Elizabeth: 8030
Palgi, Yuval: 4020
Palimaru, Alina: 3340
Palleschi, Mary K: 3335
Pallis, Maryssa: 4115, 7670
Palmberg, Kyle: 7455
Palta, Priya: 1000, 2525
Paluch, Rocco A: 1275
Pan, Minmin: 4440
Pan, Tiffany: 8020
Pan, Xi: 3505
Pan, Yinghao: 7425, 7930
Pan, Yu-Ten: 7055
Panda, Satchidananda: 2005, 4210, 7015
Pandey, Atul Kumar: 7470
Pandsheno, Sepehr: 4320
Panesar, Simran: 8000
Pang, Vincy Wing Sze: 7640
Panos, Raquel: 3460
Paola Gomezjurado Gonzalez, Laura: 1575
Paoletta, Camille: 7265
Paone, Deborah: 2010
Papazyan, Anna: 7735
Pappa, Sara T: 1300, 3460
Pappadis, Monique R: 4355, 7405
Parekh, Rupal M: 1105, 8010
Parella, Morgan: 3235
Pargai, Dhruv: 8010
Parikh, Alexander: 8085
Parikh, Romil R: 3090, 4245
Park, Bohye: 1140
Park, Byoung-Jun: 7460
Park, Caroline: 7480, 7900
Park, Chan Mi: 3135, 7080
Park, Chang Gi: 7080, 8015
Park, Cherrie: 7605
Park, Chung Hyuk: 7295, 8015
Park, Elyse R: 7680, 8015
Park, Esther: 8015
Park, Eunyoung: 7265, 8000, 8010, 8015
Park, Hayoung: 7360
Park, Hee Man: 8045
Park, Hyeran: 7310
Park, Hyunki: 8010
Park, Jeongmin: 7350
Park, Ji Yeong: 7420
Park, Jisung: 7005
Park, Joanne R: 1440
Park, Joo Young: 8000
Park, Joonhyeog: 7030
Park, Joon-Hyuk: 4420, 7205, 7620, 7640, 7720, 7750, 8015, 8020
Park, Jun Ho: 8020
Park, Junmin: 7605, 8020
Park, Juyoung: 1140
Park, Juyoung: 2240
Park, Juyoung: 3440, 7640
Park, Juyoung: 8010, 8015, 8020
Park, Min Kyung: 7260, 7275, 7290, 7680, 8015
Park, Min Kyung: 8015, 9010
Park, Moonkyoung: 7355, 8015
Park, Nan Sook: 1140, 3440
Park, Pil: 8015
Park, Rinseo: 4525
Park, Saeun: 7010
Park, Seeun: 8065
Park, Serim: 8015
Park, Sojung: 1295, 3200, 4265, 7030, 7290, 7630, 7665, 8010
Park, Soobin: 3200, 7150, 7310, 7630, 8010
Park, Soobin: 8010, 1540
Park, Sooyoun: 2295
Park, Sooyoung: 7150
Park, Sophie: 4355
Park, Suah: 7735
Park, Suhyun: 7295, 8015
Park, Sujeong: 3360
Park, Sung S: 1475, 2425, 7490
Park, Sung Ji: 1380
Park, Yanghyun: 3275
Park, Yujin: 3125
Parker, Kelly RB: 1525, 8020
Parker, Lauren: 4130, 8015
Parker, Patricia: 7055
Parker, Tassy: 2335
Parker Oliver, Debra: 7270, 7490, 7665
Parker-Autry, Candace: 4350
Parkes, Christopher: 7360, 7630
Parkhideh, Arianna: 3150
Parkhitko, Andrey: 8000
Parrish, Elise: 2500
Parrott, Kathleen: 8010
Parsons, Michael: 2300
Paruchuri, Nishita: 8020
Pascoal, Tharick: 3375, 9000
Pascoe, Kelley M: 1480, 7200
Pascual-Leone, Alvaro: 7355, 7425, 7635, 7930
Pasdo, Allison: 1265
Passamonte, Michela: 7275
Passos, Joao: 3020, 4210
Patano, Arienne: 8020
Patel, Aditi H: 8020
Patel, Anup Dharmendrakumar: 9050
Patel, Himalaya: 2410
Patel, Isha: 8020
Patel, Kushang: 2450, 7655
Patel, Maulik R: 1265, 7075
Patel, Mehul: 8020
Patel, Nikil: 7320
Patel, Purvi: 2145
Patel, Raj: 4100
Patel, Rhianna: 7720
Patel, Roshni: 7200, 7205
Patel, Sweta: 2185, 7295
Pathiravasan, Chathurangi: 7360, 8015
Patil, Shilpa: 7700
Patille, Rachelle: 2560
Patino, Joshua: 8015
Patrician, Patricia A: 8000
Patrick, Julie Hicks: 1360
Patskanick, Taylor: 1095, 3245, 7090, 7610
Patterson, Davis: 2500
Patterson, kayla: 4420
Patterson, Logan: 1190
Patterson, Mary: 8015
Patterson, Sarah E: 3165
Patti, Gary J: 1025
Pattiam Giriprakash, Pavithran: 8055
Patusco, Rachael: 7455
Patyal, Pankaj: 7465, 8000
Pau, Massimiliano: 1235
Paudel, Anju: 3230
Paul, Anirban: 8000
Paul, Constanca: 8015
Paul, Robert H: 4225
Paulsen, Adam J: 4300
Pauly, Theresa: 4410, 7510, 7915, 8020
Paun, Olimpia: 2215, 3500
Pavlik, Jan: 8000
Payne, Emma: 2410
Payne, Laura L: 7545
Payonk, Cassie: 8015
Peacock, Kimberly: 2295
Peak, Katherine D.: 4160, 7270
Pearman, Ann: 7225
Pecina, Susana: 7455
Pecker, Lydia: 8000
Peckham, Allie: 3015, 4010, 9000
Pedersen, Maja: 3005
Pedersen, Mette Merete: 4075
Peduzzi, Peter N: 3340
Peel, Emily: 7215
Peeples, Amanda D: 3110, 7135, 9025
Peeples, Landon B: 1210
Pei, Heming: 4550, 8010, 8085
Pei, Lijun: 8085
Pei, Xingtong: 1475
Pei, Yalian: 8020
Pei, Yaolin: 1380, 3305, 3505, 4030
Peinado-Martinez, Ignacio: 3150
Peine, Alexander: 3315
Pelcher, Lindsay: 9025
Pelkmans, Jordan L: 2015
Pellerin, Jim: 4110
Pemmasani, Deepishka: 3050, 3235
Pena Dias, Jenny: 7285
Pence, Brandt D: 8000
Pendergrast, Claire: 4105, 7145
Peng, Changmin: 1360, 8050
Peng, Huamao: 7725, 7745
Peng, Siyun: 2120
Peng, Wenting: 1235, 1365, 2040, 2255, 2530, 4055, 7080, 7155
Peng, Xinyuan: 3480, 7725
Penger, Susanne: 1555
Peniche, Jorge: 1320
Pennavaria, Adam: 8010
Penning, Nehle: 8010
Pepin, Marc: 7080
Pepin, Renèe: 8020
Perales-Puchalt, Jaime: 8000
Peralta, Sawyer: 8000, 8000, 8000
Perazza, Lais: 8015
Percival, Nicole: 7465
Perepelkin, Jason: 4440
Perepezko, Kate M: 2190, 3575
Perera, Rohini: 7420
Perera, Subashan: 4215
Perez, Adriana: 7000
Perez, Hector: 7620

Perez, Rene: 4475
Perez, Sasha: 3105
Perez-Aguilar, Giselle: 1245
Perez-Jolles, Monica: 8020
Perez-Rodriguez, Rodrigo: 3150
Perissinotto, Carla: 2520
Perkhounkova, Yelena: 7650
Perkins, Anthony J: 8020
Perkins, Cade: 7005
Perkins, Connie J: 7065
Perkins, Fatima: 1265
Perkins, Molly M: 8020
Perkins, Rebekah: 3465
Perone, Angela K: 2245, 3365, 7540
Perreault, Elisabeth: 4325
Perry, Brea: 2120, 8025, 9015
Perry, Marieke: 7640
Perry, Tam E: 1295, 2155, 2560, 3170, 4265
Perryman, Collin: 7455
Persson, Annelie: 8020
Perzynski, Adam T: 7235, 7690
Perzynski, Adam: 8000
Peschin, Sue: 8020
Peters, Kelly: 4375
Peters, Volletta: 3010
Peterson, Colleen M: 3130, 7520, 8020
Peterson, Courtney: 2005
Peterson, Daniel: 8015, 9015
Peterson, Krysta: 3380
Peterson, Lindsay: 1230, 2185, 2370, 3485, 3495, 7435, 7455
Peterson, Matthew: 8015
Peterson, Tina L: 7065
Petito, Lucia: 9030
Petroff, Lisa: 7720
Petrovic, Milenko: 8015
Petrovsky, Darina: 3500
Pettigrew, Corinne: 2525, 3070, 3555, 7015, 7200
Pettis, Jennifer L: 2485, 8000, 8015
Pevie, Noah: 8010
Pfeifer, Valeria: 8020
Pflieger, Lance: 8000
Pfoh, Elizabeth: 7235, 8000
Pfund, Gabrielle N: 1570, 8000
Pham, Anh: 8010
Pham, Jason: 8025
Pham, Julie Q: 8020
Pham, Thuy: 8000
Pham, Vivian: 7525
Phang, Howard J: 1435, 7010, 8000
Phatak, Vaishali: 2205, 3285
Phelan, Elizabeth: 1060, 4470, 7655
Phianhasin, Lalipat: 8000
Philippi Jr., H. George: 8010
Phillip, Jude: 4005
Phillips, Casey: 1465
Phillips, Christine: 1185, 7735, 8000, 8015, 8020
Phillips, Karon: 1315
Phillips, Kristin: 7320
Phillips, Mirelle: 4370
Phillips, Natalie A: 4300
Phillips, Patrick C: 7010
Phillips, Stuart: 4260
Phillips, Susan: 2060
Phillipson, Chris: 1180, 4275
Phinney, Alison: 3370, 4435, 7040
Phung, Quynh: 1190
Piaseu, Noppawan: 8015, 8015
Piazza, Jennifer R: 7000, 7475
Picchiello, Matthew C: 8000
Piccinin, Andrea M: 8010
Pichardo, Abel: 1335
Pichora-Fuller, M. Kathleen: 4300, 7225, 7635
Pickering, Andrew M: 8020
Pickering, Carolyn E: 1510, 2215, 4355, 7040
Pickett, Andrew C: 3190, 7005, 8020
Piechota, Amanda: 7245
Piedra, Lissette M: 8010
Pien, Chien-Jung: 7080
Pierce, Matthew J: 7030, 7455
Piersol, Catherine: 4340
Pike, James R: 1000, 1055, 4440, 7140
Pike, Kenneth C: 1400
Pilkinton, Patricia: 8015
Pillemer, Karl: 2150, 2300, 4365, 7270
Pilling, Luke C: 9015
Pimentel, Camilla B: 2430, 7110, 8010
Pinardi, Elena: 4315, 7010
Ping, Yongjing: 2065, 3025
Pino, Lilia D: 1490
Pinto, A Alex: 4300
Pinto, Jayant: 7060
Pirone, Judi: 2450
Pisu, Maria: 7495
Pitawanakwat, Karen: 2395
Pituch, Keenan: 7015, 7260, 8020
Pitzer, Kyle: 1165, 7490
Plasencia, Rosa: 4245
Plassman, Brenda: 2090, 3280, 9030
Platt, Katherine: 2555
Pleasants, Hanna: 7060
Pless Kaiser, Anica: 2385
Plotzke, Michael R: 2180
Plummer, Narcissa: 4225
Plunkett, Laura: 7110
Plys, Evan: 1345, 4370, 8000, 8020
Poblete, Ronald: 4235
Podber, Naomi: 4505
Podosek, Alexandra: 7475
Poe, Abigail: 7615
Poettcker, Karli R: 8020
Poghosyan, Lusine: 8010
Pohl, Janet: 7260
Pohnert, Anne M: 1490
Polacek, Laura: 2385
Polenick, Courtney A: 1200, 7440, 8020
Poliakov, Oleksandr: 7505
Poljanska, Ewa: 4310
Polk, Donna: 3345
Polsky, Daniel: 4460, 7470
Polychronopoulou, Efstathia: 8010
Pomeroy, Mary Louise: 1225, 2230
Pond, Brittney: 4025, 7665, 8025
Ponsiglione, Alfonso Maria: 2240, 7305
Ponstein, Klarissa: 7685
Pontiff, Mattie: 2135
Poon, Leonard: 1385
Pope, Natalie E: 3340
Pope, Natalie D: 7065, 7215
Popejoy, Lori L: 1530, 8010
Popelsky, Braden K: 7300, 7720
Popescu, Mihail: 8015
Popivker, David: 8020
Popov, Vladimir: 7075
Porter, Ben: 4120
Porter, Laura: 4470
Porter, Martha: 7740
Porter, Michelle M: 1480, 2440, 3185, 3310, 4000
Porter Starr, Kathryn N: 2485, 8015
Portz, Jennifer D: 2085, 4370, 7065
Pospisilova, Marie: 2425
Post, Emily K: 7235, 7685
Pot, Anna: 7255
Potter, Claire: 2425
Powell, Danielle: 4300, 7140
Powell, Georgia: 1105
Powell, Kimberly: 8015
Powell, Laura: 8000
Powell, Leonard: 8010
Powell, Wizdom: 4165
Powell-Cope, Gail: 2145
Power, Shannon R: 8010
Powers, Dave: 2085
Powers, Melissa: 4040
Powers, Sara M: 1030, 7465, 8020
Pradhan, Rohit: 1425, 1550, 7510
Pranoto, Inez K A: 7320
Prasad, Anyah: 3065, 3075, 8080
Praska, Nicole A: 7510, 7640
Preble, Edward: 1370
Preece, Natasha: 9000
Preisser, John S: 1520
Pressler, Susan: 8000
Preston, A'mie: 7360
Pretzer-Aboff, Ingrid: 8010
Preuss, Benedikt: 2140
Prevratil, Michael: 8020
Price, Andrea: 4510
Price, Ashley N: 7205
Price, Brenda: 1295
Price, Catherine C: 1445
Price, Quentin: 8000
Price, Sherry: 7315
Prichett, Laura: 2230
Prieto, Lucas R: 7345
Prina, Matthew: 2365, 3125
Prine, Halle: 8000
Pritchard, Kevin T: 3135
Pritchard, Sharon: 7635
Prizment, Anna: 7010
Probst, Karen: 7655
Procter-Gray, Elizabeth: 3245, 7260, 7695, 7935
Procter-Gray, Elizabeth: 8010
Prophater, Lorna: 7465
Prosperi, Mattia: 8015
Prosser, Dina: 7010
Prud'hommeaux, Emily: 4235
Pruskowski, Jennifer A: 2345, 7645
Prusynski, Rachel A: 4100, 7080, 8010
Psenka, Riley: 7680
Pu, Debbie: 2145
Pu, Fan: 8055
Puangtong, Inchai: 7460
Pucciarelli, Gianluca: 7245
Pudelek, Kelly: 8010

Puga, Frank: 2375, 7285, 7425, 7615, 7700, 7930
Pugh, Mary Jo: 2545
Pulkki, Jutta: 2460
Punke, Elizabeth LA: 7440, 7490
Puterman, Eli: 3210
Putnam, Michelle: 4135, 4475, 7260
Puts, Martine: 4175, 8015
Putzier, Alexandra: 7075
Puurveen, Gloria: 7510, 7915
Pynoos, Jon: 1005

**Q**
Qato, Dima M: 9020
Qi, Andy: 8000
Qi, Bingbing: 8010
Qi, Lu: 7460, 7715
Qi, Xiang: 1380, 3305, 3505, 4030, 7630, 8000, 8025
Qi, Yan: 1355
Qian, Wenqing: 7525
Qian, Yiqing: 2230, 3570
Qian, Yong: 9015
Qian, Yuting: 3370, 7490
Qin, Tian: 8025
Qin, Weidi: 1060, 2020, 3045, 3245, 4190, 4430, 7490
Qin, Yue: 2001, 3225
Qin, Zeyu: 7330
Qiu, Qian: 2450
Qiu, Xiao: 1535
Qiu, Xichenhui: 7625, 7710, 8000
Qiu, Yiming: 7695
Qu, Doria: 8020
Qu, Shan: 7065, 7270, 8015
Qu, Xiaomin: 3505
Quach, Emma: 7110, 7420
Quach, Ha-Linh: 1255, 2065
Quach, Lien: 8015, 9050
Quashie, Nekehia T: 2515, 8000
Quiñones, Ana: 3035, 3540, 4160, 7270, 7300, 8010
Quiñones-Cordero, Maria: 3045
Quiben, Myla: 7330
Quigley, Molly: 7355
Quiles, Penelope: 7000
Quillen, Ellen E: 3045
Quinlan, Olga: 2370
Quinn, Ryan: 3385
Quintero, Laura: 2060

**R**
Rababa, Mohammad: 7320
Rabalais, Jennifer: 4450
Rabin, Laura A: 2160
Rabinowitz, Jill: 1255, 3070
Rachmuth, Lisa: 2001
Raciti, Audrieanna E: 7510, 7910
Radeck, Leon: 1040
Rademaker, Alfred: 9040
Rafoul, Bahaa: 3085
Ragab, Ahmed: 2160, 7735, 8010
Ragavan, Maya: 3045
Raghavachari, Nalini: 1025
Rahemi, Zahra: 3175
Rahman, Ashikur: 4100
Raj, Minakshi: 2060, 4270, 7270, 7660
Rajabali, Saima: 8000
Rajendra, Anil: 3330
Raji, Mukaila: 2090, 4095, 7405, 8010
Rakel, Barbara: 7205
Rakotoarivelo, Harivony: 4550
Ralser, Markus: 7210
Ralston, Margaret: 4040
Ram, Nilam: 1575, 4525
Ramaiyer, Malini: 4045
Ramamurthi, Hema: 8095
Raman, Rema: 8000
Ramdass, Jeff V: 7490
Ramirez, Maggie: 2125
Ramirez Gomez, Liliana: 4130, 7270, 7660
Ramirez Mantilla, Mariana: 8000
Ramirez-Matias, Julliana: 2310
Ramirez-Zohfeld, Vanessa: 7005, 7420, 7490
Ramos, Carmen: 8000
Ramos, Manuel: 8010
Ramos, Sofhia V: 4310, 8020
Randa-Beaulieu, Cari: 1555, 3205, 7510, 7915
Randall, Kevin: 7150
Rane Levendovszky, Swati: 8000
Rangan, Apoorva: 1575
Rank, Jennifer: 3380
Rantanen, Taina: 1285
Rao, Smitha: 2070, 2195, 3485
Rapp, Stephen R: 3180
Rappaport, Noa: 8000
Rataj, Alison C: 2000, 3075, 4270, 4450, 7320
Rathakrishnan, Sharmini: 3105
Rathbun, Alan M: 7010, 7440
Ratnayake, Aneeka: 4225
Rauer, Amy: 4525, 7070
Rauktis, Mary E: 1290
Rauzi, Michelle R: 1275, 2135, 7155
Raval, Aasha D: 8035, 8070
Ravdin, Lisa: 7630
Ravel, Jacques: 7060, 7210
Ravi, Vishnu: 8015
Ravindran, Malcolm S: 1015, 2185
Rayan-Gharra, Nosaiba: 7400, 7920, 8010
Raye-Brown, Sharnette: 7405
Raymo, James: 7265
Raza, Sakeena: 7070
Razo, Christian: 7040
Ream, Molly: 2385
Reardon, Claire: 1480
Reavis, Kelly M: 7310
Rebadulla, Hannah: 8070
Rebok, George W: 4490, 9000
Recenti, Marco: 2240, 7305
Recker, Amy: 3240
Reckrey, Jennifer: 3105, 3570, 4480, 8010, 8070
Reczek, Rin: 7225
Redd, Tara: 7425, 7910
Reddy, Ann: 1530, 3240, 8010, 8015
Redmond, Leslie: 7480
Reed, Alexandra M: 7200
Reed, David: 1520
Reed, May J: 8000
Reed, Nicholas: 1055, 2525, 4300, 7140
Reeds, Dominic: 1275
Reese, Alexandria C: 3375
Reese, Elaina: 4070
Reese, Jordan: 4130, 4340
Reese-Melancon, Celinda: 7065, 7225, 8020
Reeves, Mathew: 2525
Regan, Claire: 8010
Regier, Natalie G: 8000
Regitz-Zagrosek, Vera: 7030
Regos-Stewart, Dalia: 7200
Rehkopf, David: 1575, 4035, 7350, 8020
Rehman, Haseeb: 8020
Reid, Colleen: 4430
Reid, Katrina: 7105
Reid, Kieran: 3350
Reid, M. Carrington: 7080, 7270, 7630, 8020
Reif, John: 2300
Reifsnyder, JoAnne: 2140
Reiko, Kanaya: 7330
Reilly, Elizabeth: 8010, 8015
Rein, David: 1115
Reinhartz, Alice: 4490
Rejeski, W. Jack Jack: 1410
Rekant, Julie S: 7720
Relerford, Raven: 7005
Remillard, Elena T: 3415, 9025
Ren, Cheng: 7030
Ren, Haopu (Lily): 1115, 2410, 7750, 8015
Ren, Jinnan: 7070, 8020
Ren, Lily: 3205
Ren, Yuanyi: 7605
Rendell, Peter: 8020
Renn, Brenna N: 1325, 7235, 7685
Rensema, Elspeth: 2450
Renzetti, Lucrezia: 4100
Research Team, TACAD: 7270
Resendes, Kelly: 2370
Resnick, Barbara: 3050, 3255, 3555, 4090, 4205, 7040, 7720, 8020
Resnick, Susan M: 2510
Reuben, David: 3340
Rey, Sergio: 4095
Reyes, Jimmy A: 3550, 4205
Reyes, Joel: 8020
Reyes, Mariana A: 7015, 7200
Reynolds, Addam S: 4400
Reynolds, Chandra A: 4095, 7225, 8020
Reynolds, Courtney: 1145, 7405
Reynolds, Tera: 1070
Reynolds, Zahra: 4225
Rezapour, Rezvaneh: 4220
Rhee, Min-Kyoung: 1140
Rhoads, Kristoffer: 4285
Rhodes, Annie S: 4290, 4510
Rhodes, David: 8015
Rhodes, Ramona: 8015
Rhodes, Shanae L: 9010
Riang'a, Roselyter M: 4380
Rianon, Nahid: 3135
Ribeiro, Oscar: 7745
Ricciardi, Carlo: 2240, 7305
Rice, Elise: 1345
Rice, Ian: 9025
Rice, Jordyn: 1365
Rice, Ketra: 1235
Richard, Patrick: 3030
Richards, Emily A: 2045
Richards, Kathy C: 3240

Richards, Nicole: 7005, 7540
Richards Adams, Ingrid K: 7490, 7935
Richardson, Daniel: 8035
Richardson, Julie: 7655
Richardson, Katy: 7440
Richardson, Lena Rebecca: 2560
Richman, Alice: 7530
Richmond-Rakerd, Leah: 7695
Richter, David: 1215
Rickenbach, Elizabeth: 2130
Riding, Bethany: 7480, 7900
Riegel, Barbara: 3385, 7440, 7925
Riffin, Catherine: 8020
Rigdon, Joseph: 1090
Rigotti, Nancy A: 8015
Riklon, Sheldon: 8000, 8015
Riley, Jessica: 2430, 8010
Rimar, Alexander: 4100
Riminchan, Anna: 2565
Ring, Lia: 8010
Rink, Andrea: 7410, 7900
Ripperger, Hayley: 4315
Rippon, Isla R: 1225
Risacher, Shannon: 8000
Riser, Tiffany: 2010, 4360, 7445, 7935
Ristl, Christina: 2300
Ritchey, Katherine C: 2135, 3435, 4285, 7205
Ritchie, Christine: 1345, 2315, 3250, 4370, 9020
Ritsema, Chad: 4325
Ritter, Aaron: 3010
Ritter, Ashley Z: 3550, 7290
Rivas, Daniel: 8020
RIvas, Jennifer: 7695
Rivera-Hernandez, Maricruz: 1075, 1195, 8010, 8020
Rivers, Mia: 7000
Rizzo, Samantha M: 7640
Rizzo, Victoria: 8010
Rizzuto, Debora: 4315
Roa Diaz, Stephanie: 4170, 8000, 8000
Roache, Tavia: 8020
Robbins, Ronna: 2505
Roberson, Donna: 7510
Robert, Stephanie: 7160, 7660
Roberto, Karen A: 3190, 7660
Roberts, Amy R: 1270
Roberts, Angela: 9040
Roberts, Jackson A: 8085
Roberts, Jimmie E: 8015
Roberts, Mitchell: 8015
Roberts, Pamela S: 3300
Roberts, Scott: 1085
Roberts, Shelby: 3430, 7005, 7425, 7910
Roberts, Summer: 7415
Roberts, Tonya: 2550, 3090, 7510, 7705, 8010
Robertson, Frank: 1020
Robertson, Neil: 3325
Robillard, Ashley: 2325
Robillard, Julie M: 2565, 7430
Robins, Amy: 3570
Robinson, Kristen: 2070, 7205
Robinson, Laura: 9010
Robinson, Max: 8000
Robinson, Randee: 1400
Robinson, Renee: 8010
Robinson, Stephanie: 7720
Robinson-Lane, Sheria: 7030, 7665
Robison, Julie: 2495, 7740
Roche-Dean, Maria V: 1045, 1465, 4475
Rochon, Elizabeth: 4370
Rochon, Paula A: 3520, 8010
Rockette, Ra'Shida S: 7000
Rockwood, Kenneth: 7275, 8000
Rodill, Zena: 3075
Rodríguez-Artalejo, Fernando: 3330
Rodríguez-Blázquez, Carmen: 4045
Rodrigues, Annette E: 8010
Rodrigues, Ricardo: 2060
Rodriguez, Jacob: 7010
Rodriguez, Misha: 3550
Rodriguez, Rachel L: 2280
Rodriguez, Robert A: 8010
Rodriguez, Tania M: 7135
Rodriguez Colmenares, Nury A: 7425, 7930
Rodriguez Tiburcio, Alan: 8010
Rodriguez-Galan, Marta: 7445
Rodriguez-Manas, Leocadio: 3150
Rodriquez, Jason: 7490
Roe, Lauren S: 7060
Roecke, Christina: 1220, 4185
Roeland, Eric J: 7020, 7205
Rogalski, Emily: 9040
Rogers, Geoff: 2150
Rogers, Megan: 4510
Rogers, Wendy A: 1040, 3415, 4130, 4270, 7360, 7660, 8000, 8010, 8015, 8020, 8035, 9025
Roh, Soonhee: 7425, 7500
Rojas-Saunero, L. Paloma: 1545, 4375
Rojina, Janet A: 1480, 7200
Rolbiecki, Abigail: 8000, 8010
Rollandi, Isabel: 4365
Romañach Álvarez, Lourdes S: 7485, 7925
Roman, Anna: 1190
Roman, Carly: 8010
Romanelli, Meghan: 4495
Romero Saenz, Janette: 7285
Rompilla, David B: 3355
Rong, Jian: 7360, 8015
Ronis-Tobin, Victor: 8010
Rook, Erin: 3490
Rooks, Ronica N: 1415, 8000
Rooney, Alicia L: 3215
Roopsawang, Inthira: 7205, 7440
Root, Eve Z: 7055, 7475
Roque, Leslye: 7080
Roque, Nelson: 7750, 8000, 8020, 9020
Rorai, Vanessa O: 2155, 3170
Rosa, Paola: 8015
Rosales, Anrea: 2565
Rosales-Soto, Giovanni: 7305
Rosano, Caterina: 1055, 1240, 2220, 2510, 7640, 8000
Rosas, Ivan: 4170
Rose, Andrew: 7150
Rose, Grace: 7720
Rose, Jeff: 1295
Rose, Karen M: 7490, 7660, 7935, 8020
Rose, Liam: 7080
Rose, Tara: 8015
Roseman, Jeffrey M: 2160
Rosen, Anthony E: 1270
Rosen, Sonja: 2520, 7320, 7350
Rosen, Tony: 4195, 4355, 7405
Rosenberg, Dori E: 1265, 7720, 8010, 9020
Rosenblatt, Lucy: 4165
Rosenbloom, Michael H: 3510
Rosendaal, Nicole: 3535
Rosenstein, Benjamin EM: 2535, 7645
Rosland, Ann-Marie: 4195
Ross, Allison D: 8000
Ross, Lesley A: 1185, 1250, 2055, 2330, 7735, 8000, 8015, 8020
Ross, Sarah: 1065
Rosselli, Mónica: 7360, 7425
Rosser, B.R. Simon: 2225, 3490
Rossi, Martina: 7305
Rossnagel, Emma: 3205
Rosso, Andrea L: 1240, 2510, 3375, 4215, 7300, 8000, 8015
Rosteius, Katharina: 4345
Rotblatt, Lindsay J: 7005
Rote, Sunshine: 1495, 3440, 8020
Rotenberg, Shlomit: 1205, 8085
Roth, David L: 1255, 3130, 7225
Roth, Jennifer C: 4060
Rothenberg, Craig: 1130
Rothermund, Klaus: 1375, 4020
Rothgang, Heinz: 2140
Rouse, Sasha S: 2065
Rousseau, Julie: 2340, 7405
Routh, Brianna N: 8020
Rowan, Nicholas R: 2080
Rowe, John: 7350
Rowell, Londi: 3235
Roy, Arka: 4190
Roy, Brita: 7535
Roy, Jason: 4485
Roy, Mishy: 3510
Royo, Sofia: 3280
Royse, Sarah K: 3375
Rozario, Philip: 8010
Rozenberg, Aliza: 7060
Ruan, Iris Xiaoyu: 7640
Ruan, Lei: 7080
Rubin, Leah: 8020
Rubinstein, Stephanie R: 7460, 8020
Rubio, Olivia C: 3150, 4420
Rubovits, Eve: 7270
Rubtsova, Anna: 8015
Rudek, Michelle: 8000
Rudoler, David: 7295
Rudolph, Catherine S: 7350
Ruggiano, Nicole: 4060, 7095, 8000
Ruggles, Steven: 4035
Ruiz, Diana: 7360, 7675, 8010, 8070
Ruksakulpiwat, Suebsarn: 8000
Rule, Payton D: 7745, 8000

Rundek, Tatjana: 8085
Ruopp, Marcus: 8020
Rupp, Hayley: 3585
Ruppar, Todd: 3500
Rupper, Randall: 4520
Rupprecht, Fiona S: 1375, 2300, 7245
Rush, Jonathan: 1525, 4180
Rushing, Baylie: 7720
Ruskin, Andrea: 7475
Rusmevichientong, Pimbucha: 7000
Russell, Andrea: 4480
Russell, David: 7740
Russell, Kristy: 3100
Russell, Luke T: 4165
Russo, Frank A: 7225
Ruth, Terrance: 3275
Rutledge, Emily: 7630, 7905
Rutledge, Jocelyn A: 4410
Ryan, Alice: 7010, 7440, 8000
Ryan, Healey: 7465
Ryff, Carol D: 8000
Ryu, Byeongju: 1295, 4140, 4265, 7290
Ryu, HyeRim: 1080, 7695
Ryu, Jungsu: 7535
Ryvicker, Miriam: 4360

**S**

Sabatini, Serena: 1285
Sabbatini, Amber: 8010
Sabol, Jacqueline: 7410
Sacco, Alessandra: 8000
Sacco, Paul: 7020
Sachs, Greg: 4440
Sadarangani, Tina: 4130
Sadeq, Nasreen: 3495
Sadeqi, Farima: 8000
Sadler, Richard C: 4430
Sadruddin, Sabeen: 7270
Saenz, Joseph: 1320, 2515, 4455, 8000, 9000
Saffel-Shrier, Susan: 7315
Sagehorn, Mallory A P: 3375, 7420
Sagong, Hae: 7095, 7275
Saha, Troyee: 7155
Sahab, Lujain: 7060
Sahawneh, Fursan: 4000
Sahli, Michelle W: 7155
Sahni, Shivani: 2080
Said, Adam: 8000
Saikia, Nandita: 1195
Sailors, Olivia: 8015
Saint Onge, Jarron: 4330
Saito, Taeko: 7400, 7920
Saito, Yasuhiko: 3290, 7205, 7265
saiyed, sahebi: 8020
Sakai, Tomomichi: 7275
Sakal, Collin: 8020
Sakamoto, Mariko: 3370, 7510
Sakata, Kei: 7130
Salama, Mohamed: 1155
Salari, Sonia L: 7030
Salas, Joanne: 7275
Salbach, Nancy M: 7300
Salcido De Pablo, Pamela: 7400, 7920
Sales Martinez, Sabrina: 7540, 7735
Saliba, Debra: 4100, 9040
Saligan, Leorey: 2445
Salinas Navarro, Sheila A: 7725, 8010, 8020
Salisbury, Dereck L: 7015, 7450, 8020
Salive, Marcel: 1390
Salminen, Lauren: 7495, 7930
Salmon, David: 1485
Salner, Andrew: 2295
Saltvedt, Ingvild: 7060
Salvo, Erin: 2150, 7405
Salzman, Talia: 2510
Samelli, Alessandra G: 1320
Samper-Ternent, Rafael: 1320, 2075, 3540, 7215
Samson, Andrew: 7040
Samuel, Linda: 8000
Samuelson, Brady: 7275, 7275
Samus, Quincy M: 2015, 2155, 3215, 4100, 7005, 7465
San Roman, Irene: 1395
Sanabria Véaz, Karla: 7155
Sanches Yassuda, Mônica: 8015
Sanchez, David: 7405
Sanchez, Travis: 8015
Sanchez, Victoria: 7140
Sanchez-Contreras, Monica: 7075
Sanchez-Reilly, Sandra: 8010, 8015
Sanders, Edie C: 8000, 8020
Sanders, Lisbeth: 8020
Sanders, Margaret: 7475, 7900
Sandhu, Sahir: 7210
Sandoval, Jacqueline A: 8010
Sandoval, Moises H: 3290
Sandoval Garrido, Felipe A: 7605
Sandri, Tina: 8010
Sands, Laura: 2495, 4110, 4315, 7680, 8010
Sands, Talyah: 3430, 1260
Sanford, Claudia: 1295, 4265
Sanford, Jon A: 8010, 8020
Sangrar, Ruheena: 8085
Sanner, Haley K: 7285
Santaella, Paulina: 8010
Santaella-Tafolla, Paulina: 7510
Santana, Layla Katharine: 7455
Santangelo, Kelly: 7075
Santilli, Josie N: 1185, 2130
Santioni, Margaret: 7720
Santora, Kenneth: 1340, 2210
Santos, Suzanne T: 4465
Santos-Lozada, Alexis R: 1350, 4050, 7350
Santoso, Kate: 7645
Santoyo-Olsson, Jasmine: 3175
Sanz Simon, Sharon: 7350
Sapir, Adi: 3465, 4040
Sapkota, Krishna P: 3055
Sappington, Erica: 8015
Sapra, Mamta: 4060
Saraboon, Natthayawadee: 7440
Sardina, Angie L: 8020
Sargent, Lana: 3565, 4510
Sarkar, Nilanjan: 7155, 7435
Sarma, Anya: 2410
Sasai, Hiroyuki: 7205, 7540
Sasdi, Olivia: 7215
Sasser, Jennifer R: 1175, 7415, 8010
Sathish, Tanish: 8000
Sathler Tavares Batistoni, Samila: 8015
Sato, Kana: 4135
Satoh, Kana: 4135
Sauer, Jennifer H: 7125
Sauerteig-Rolston, Madison R: 3405
Saunders, Michele J: 3505
Saunders, Pamela A: 2275, 3365
Saunders, Stephanie: 7655
Sausman, Kathryn: 8015
Savage, Rachel D: 8010
Savard, Grace M: 3375, 8020
Savary, Patricia E: 8015
Saville, Neika N: 2340
Savin, Douglas: 8010
Savla, Jyoti: 3190, 4060, 7660, 8020
Savundranayagam, Marie Y: 1070, 2410, 7465, 7635, 7910
Saw, Anne: 7490, 7935
Sawyer, Linda: 8015
sayles, anijah: 7000
Saylor, Martha Abshire: 2230
Scales, Kezia: 2500, 3570, 4290
Scambray, Kiana A: 1495, 9015
Scandalis, Lina: 8000
Scarpelli, Michael: 7695
Sceppa, Carmen: 2110
Scerpella, Daniel L: 4130
Schüz, Benjamin: 4345
Schaaf, George W: 3045
Schaefer, Adam: 2300
Schaefer, Caroline: 3135
Schaefer, Cathy A: 1545
Schaefer, Sydney Y: 7200, 7325, 9015
Schafer, Donna E: 2250
Schafer, Marissa: 3400
Schafer, Markus: 3410
Schaff, Branden: 4120
Schaffer, Sigourney: 4320
Scharlach, Andrew E: 1195
Schechtman, Kenneth B: 1275
Scheffer, Julian A: 3285, 8040
Schenck, Sabine: 7440, 7490
Schepens Niemiec, Stacey: 3165
Schere, Laura D: 8020
Scherr, Susan: 8020
Scherrer, Jeffery: 7275
Scheunemann, Leslie P: 3045, 4530
Schexnayder, Julie: 7285
Schierer, Allison: 7005
Schilling, Birgit: 2435, 3045, 4170
Schilling, Oliver K: 1285
Schiloski, Kylie A: 1525, 7455
Schilt, Kristen: 1110
Schiltz, Nicholas K: 1490
Schilz, Jodi: 8015
Schindel Martin, Lori: 4445
Schlag, Karen E: 4355, 7405
Schlomann, Anna: 1040
Schmidt, Laura: 8015
Schmidt, Steven: 1285, 3170, 7155
Schmiedeler, Anthony: 1135
Schmitter-Edgecombe, Maureen J: 2360
Schneider, Augusto: 8000

Schneider, Claudia: 8000
Schneider, Kevin: 2530
Schneider, Stefan: 2465, 3335
Schneider, Sue: 3575
Schneider, Sydnie: 1335, 3385, 4435, 7065, 7425
Schoenborn, Nancy: 4220
Schoenfeld, Elinor R: 3265, 3415
Schoenhofen Sharp, Emily: 1215
Scholes, Shaun: 2365, 3125
Scholler, Emily: 8015
Schomburg, Lutz: 7210
Schousboe, John: 4055, 7630
Schrack, Jennifer: 8000
Schrack, Jennifer A: 1240, 1255, 2035, 2525, 3070, 7540, 8015
Schrager, Sarina: 4160
Schramm, Eric: 7360
Schrauf, Robert: 3550
Schriner, Samuel E: 7010
Schroepfer, Tracy A: 4080
Schubert, Carla R: 4300
Schuh, Madyson: 4510
Schuller, Jeanne: 2010
Schulman-Green, Dena: 4360
Schultz, January: 7040
Schultz, Leslie: 2300
Schumacher, Alexander T: 1215
Schumacher, John: 2535, 3495, 8010
Schumacher, Linus: 3325
Schumann, Annette: 1070, 2410, 7635
Schumm, Phil: 1110
Schurr, Matthew: 7235, 7685
Schutte, Debra: 8020
Schwarte, Liz: 7200
Schwartz, Abby J: 7530
Schwartz, Andrea W: 8015
Schwartz, Anna: 1175
Schwartz, Heather: 8020
Schwartz, Tatum A: 3065
Schwarze-Taufiq, Tiara: 7320
Schwingel, Andiara: 7145
Scior, Katrina: 8000
Scott, Cathy B: 8000
Scott, Gregory: 7310
Scott, Julia A: 7200
Scott, Julia C: 7695
Scott, Stacey B: 4185, 4280, 4410, 8020
Scott, Stephanie L: 7540, 7735
Scott, Victoria: 1535
Scott-Poe, Deneisha S: 7005
Scow, Laura: 2525
Scrivano, Rachel: 2130
Scroggins, Laila: 7000
Scully, Madeleine: 7680
Seago, Elayna: 8020
Seal, Susan D: 4040
Seaman, Aaron: 1395, 7465, 7685
Seaman, Ilona: 1490
Seaman, Jennifer B: 7200
Seaton, Thomasina: 8000, 8020
Sebastiao, Emerson: 7260
Seblova, Dominika: 4375
Seckin, Gul: 7360
Sedaghat, Sanaz: 7010
Sedey-Seitz, Cory: 1415
Sedikides, Constantine: 7255
Sedore, Christine: 7010
Seetharaman, Kishore: 1555, 3205
Segal, Daniel L.: 7115
Segal-Gidan, Freddi: 1430
Segawa, Dai: 7420
Segel-Karpas, Dikla: 1565, 8010
Segerstrom, Suzanne C: 7245, 8020, 9010
Segundo, Joahana: 9000
Seidel, Elizabeth: 8020
Seidenfeld, Justine: 2090
Seigel, Zachary W: 4370
Seino, Satoshi: 7275
Seitz, Aaron R: 8000
Sekhar, Rajagopal V: 1420, 2080, 2235
Sekhon, Vishaldeep: 1255, 7225
Seliger, Stephen L.: 3330
Seligman, Benjamin: 4055, 7275, 7300
Selmanoff, Mollie: 4530
Selovin, Fabio: 1375
Seltzer, Judith A: 4050
Selzler, Katherine J: 7325
Semba, Richard: 7010
Semelka, Charles T: 1390
Sen, Ranjan: 7210
Senadheera, Chatura: 8000
Seo, Jinsil: 8015
Seo, Jisu: 7275, 7640
Seo, Minhwa: 3050
Seong, Minju: 7535
Seplaki, Christopher: 7030, 7420, 7920
Ser, Sarah E: 8015
Sereika, Susan M: 7200
Serina, Peter: 1530, 8010
Serna, Myrna: 8010
Serra, Monica C: 2345, 2505, 7720
Serrano-Rubio, Luis: 7735
Sesek, Greg: 8010
Seshadri, Gokul: 2460, 7010
Seshadri, Sudha: 7500, 7930
Setoguchi, Soko: 4485
Severance, Jennifer J: 1065, 3510, 7080
Sevi, Barış: 4535, 7540
Seward, Morgan: 1345, 8000, 8020
Seward, Tanya: 7065
Sexson, Kathryn: 7065
Sexton, Athena: 1185
Shaaban, C. Elizabeth: 3375
Shade, Marcia: 1500, 4205
Shadmi, Efrat: 4075, 7265, 7400, 7920
Shaffer, Tasha M: 8020
Shafir, Irit: 1565
Shah, Avani A: 7450
Shah, Manish N: 3010, 4530
Shah, Ravi V: 7710, 8000
Shah, Sameer B: 7310
Shahid, Kainat: 9025
Shahriar, Kamal Md: 7315
Shaid, Elizabeth: 3300, 7535, 8010
Shaji, Chacko K: 2235
Shakir, Madiha: 7425, 7910
Shakya, Shamatree: 7615
Shakya, Sunita: 8010
Shaleha, Rinanda Rizky Amalia: 7750, 8000
Shalev, Idan: 7010, 7455, 7490
Shallcross, Amanda J: 1140
Shamas, Tracy: 7475
Shan, Liang: 2375
Shan, Xiaorong: 4035
Shan, Yong: 2090, 4095
Shands, Rachel: 1520
Shang, Shaomei: 7430
Shang, Yongxin: 3165
Shankar, Janki: 2050
Shannon, Kay: 7520
Shannon, Zoe Y D: 7140
Shardell, Michelle: 1535, 3035, 3330, 4385, 7010, 7210, 7285, 7440, 8015
Sharma, Anisha: 7065, 8020
Sharma, Anjali: 8010, 8015
Sharma, Dinesh: 3330
Sharma, Hari: 8075
Sharma, Isha: 7470
Sharma, Rashmi: 7530, 7905
Sharma, Shakshi: 7010, 8000, 8015, 8015, 8015
Sharninghausen, Jody: 7085
Sharon, Tal: 7025
Sharp, Bethanie: 8020
Sharp, Lisa: 7615
Sharpp, Tara J: 1050
Sharrett, Richey: 4440
Shastri, Vinita: 7480, 7900
Shatswell, L. Elizabeth: 7530
Shaughnessy, Marianne: 2280, 2345
Shavit, Yochai Z: 1575
Shaw, Amy L: 4355, 7405
Shaw, Clarissa: 1530, 7400, 7650
Shaw, Pamela A: 8010
Shayya, Ashley: 3255
Shealy, Carlisle: 2275, 2495
Shean, John: 3430, 7425, 7910
Sheardova, Katerina: 8000
Shearon, Jennifer: 7720
Shearston, Jenni A: 4035, 4375
Sheehan, Connor: 8000
Sheets, Kerry: 4055, 7630
Shelar, Shatakshi: 7660
Sheldon, Kira G: 8035
Sheldon, Marisa: 2070, 2195, 3080
Shellman, Juliette M: 7335
Shen, Bin: 4535
Shen, Huei-Wern: 7445, 7700
Shen, Jenny: 8035
Shen, Jiahao: 4195
Shen, Joseph: 8015
Shen, Karen: 1395, 8070
Shen, Lingyi: 3505, 9015
Shen, Nicola: 8015
Shen, Xinyue: 3480
Shen, Yi: 1175
Shen, Yiyi: 7015
Shen, Yuqi: 1250
Shende, Shraddha: 1405, 7015, 7455
Sheng, Xiaoming: 4520
Shenk, Dena: 8010
Shenoy, Shivani: 8035, 8070
Shenoy, Vineet: 8015
Shepherd-Banigan, Megan: 2090, 7005
Sherman, Aurora M: 8020
Sherwood, Chet C: 8000
Shi, Huidong: 7285
Shi, Jing: 7640
Shi, Junrong: 8000
Shi, Kewei: 7305
Shi, Sandra: 3135, 7080
Shi, Yang: 4110

Shi, Yanhong: 7450, 7930
Shiba, Koichiro: 7355
Shibeika, Sahar: 1475
Shibuya, Kyohei: 8015
Shida, Takashi: 8015
Shields, Stephen L: 3215
Shields, Timothy M: 3375
Shieu, Bianca: 7510, 8010
Shiferaw, Tsion: 8020
Shih, Regina A: 3340, 3570, 4245
Shih, Yao-Chi: 7095
Shilvock, Abigail R: 8015
Shim, Eun-Jung: 2365
Shim, Soyeon: 3050
Shin, Donghee: 7535
Shin, Esther: 7290, 7665, 8010
Shin, Haeun: 7460
Shin, Hyeyeon: 8020
Shin, Hyung Eun: 8000, 8010, 8015
Shin, Jinhee: 7080, 8010
Shin, Jiyoung: 7640, 7680
Shin, Juh Hyun: 7295, 8015
Shin, So Young: 7295
Shin, Soogyung: 7005
Shin, Sun-ui: 7265, 8000
Shin, Yeonsoo: 7275
Shin, YooGyung: 7275, 8015
Shin, Yumi: 1375, 7275
Shippee, Tetyana P: 1520, 2225, 3090, 3490, 4245, 4395, 4445
Shiraishi, Natsue: 8015
Shires, Deirdre: 7345
Shirk, Steven D: 3110, 7490
Shiroma, Kristina: 2265, 4330
Shiu, Chian-Huei: 7110
Shivappa, Nitin: 2080
Shoji, Kristy: 1080
Shore, Amanda: 4100
Shorr, Ronald I: 2145
Shovali, Tamar: 1185, 4240
Showalter, John: 7425, 7930
Showalter, Kathryn: 3275, 7405
Shrestha, Aman: 3055, 3330
Shrestha, Hom L: 8055
Shrestha, Lucy: 8055
Shrestha, Srishti: 2525, 4440, 7060, 7630
Shrira, Amit: 1565, 2001, 4020
Shrivastava, Jyotsna: 8000
Shtrahman, Matthew: 7210
Shukla, Rohit: 7210
Shulman, Lisa M: 8010
Shulyaev, Ksenya: 3015, 4075, 7265, 7400, 7920, 8090
Shuman, Sari B: 1260
Shuman, Stephen K: 3305
Shuman, Valerie: 4215
Shune, Samantha: 2060, 3500
Si, Wanwan: 7295, 7740
Siamdoust, Shahrzad: 9030
Siby, Ninette: 7530
Sicinski, Kamil: 3225
Sidani, Souraya: 4255
Siddiqui, Aziza: 7485
Sidle, Erin: 8010, 8015
Siebecker, Megan: 7135, 7670, 7695, 7700
Siedner, Mark J: 4225
Siedner, Mark: 8015
Siegel, Elena: 2200
Siegfried, Alexa: 1425
Sigurdardottir, Sigurveig: 2040
Silberman, Susan Lynn: 7110
Silbermann, Rebecca: 7020
Sillito, Carrie LeFevre: 7030
Silva, Jennifer: 8015
Silva, Susan: 7270
Silva Arbex Borim, Flávia: 8015
Silva de sá, Mel: 7225, 7655
Silvasstar, Josh: 7065
Silver, Michelle: 7130
Silver, Roxane Cohen: 7030
Silverman-Martin, Wendie: 8000
Silverstein, Hannah: 4000
Silverstein, Merril: 3030, 3190, 3360, 7235, 7610, 8085
Silverstein, Nina M: 1305, 3265, 3310, 7065, 7240
Silveyra, Eduardo: 7700
Sim, Hyebeen: 8010
Simard, Pascale: 8020
Simmons, Cassandra: 2060
Simmons, Sandra: 8035
Simning, Adam: 8020, 8055
Simon, Bala: 3430
Simon, Darby M: 7660, 8020
Simon, Judy: 8015
Simon, Kennedy N: 2330
Simon, Lena G: 2330
Simon, Lena: 8000, 8020
Simone, Patti M: 7200
Simonsick, Eleanor M: 1255, 3050, 3070, 3330, 7090, 7630, 7710, 7720, 8015
Simpson, Deborah: 7480
Simpson, Elizabeth: 1360, 4305, 7670
Simpson, Kit: 1075
Sin, Mo-kyung: 2160, 3555, 9025
Sin, Nancy L: 4405
Singer, Dianne: 1085
Singer, Jonathan: 1335, 3385, 4435, 7005, 7065, 7425, 9015
Singh, Amaya: 9040
Singh, Amit: 7210
Singh, Nandini: 2520
Singh, Roshni: 7675, 8010, 8070
Singh, Satya N: 4065
Singleton, Andrew: 8000
Singleton, Meki: 2180, 8020
Singsank, Katie T: 7240
Sinke, Christopher: 4490
Siperstein, Gary: 7490
Siqueira, Vitor: 7260
Siren, Anu: 3455
Sirey, Jo Anne: 4365
Sison, Stephanie M: 8015
Sison, Stephanie Denise M: 3135, 7080
Sisti, Anthony: 3240
Siu, Albert: 7535, 8010
Siudzinski, Jayne: 8010
Sivakumar, Nikita: 4005
Sivers-Teixeira, Theresa: 8055
Sixsmith, Andrew: 3060
Sixsmith, Judith: 1015, 8010
Sjöberg, Linnea: 3035
Skalla, Lesley A: 2180
Skarphol, Tricia: 1520, 2225, 4445
Skemp, Eleanor: 8000
Skinner, Jonathan: 3145
Skinner, Kelly: 2360
Skipper, Antonius D: 7150, 8010
Skirbekk, Vegard: 2425
Skoblow, Hanamori F: 3055
Skovgaard, Asmus Cosmos: 7010
Skowronski, Jordan: 8020
Skrajner, Michael: 7465
Skrzynski, Carillon J: 3115
Slaug, Björn: 1285
Slaven, James: 7465
Sliwinski, Martin J: 4405, 4515, 7225, 7455, 8020
Sloan, Caroline: 7470
Sloan, Kylie: 7410
Sloane, Philip: 1520, 2105
Slossberg, Rebecca K: 4100
Sloup, Sharon: 3240
Slowey, AJ: 8020
Small, Brent: 7070, 8000
Smichenko, Juliana: 4075, 7400, 7920
Smiley, Adrianne: 1270
Smilovsky, Kyle: 3315
Smith, Alexander K: 4480
Smith, Alexandra: 3055
Smith, Amanda: 2370
Smith, Amelia: 3090
Smith, Brian N: 2385
Smith, Brian: 8000
Smith, Brigid: 3485
Smith, Caroline: 7000
Smith, Claire: 7130
Smith, Eleanor: 3270
Smith, Eric: 7030
Smith, Gregory C: 1190, 3360, 7265
Smith, Hannah: 9040
Smith, Jacqui: 1210, 2025, 4525, 7050, 7130, 7335, 7525, 7630, 7690, 7725
Smith, Jamie M: 4300, 4360, 4460
Smith, Jason: 3220, 4440
Smith, Jennifer: 1435
Smith, Jennifer L: 3355, 7050, 7085, 7720, 8010
Smith, Julie: 7480
Smith, Kate: 1070, 3240
Smith, Kelly M: 1230
Smith, Kelly: 8000
Smith, Ken R: 4520
Smith, LaTonya: 7350, 7540, 7910
Smith, Laura T: 3575
Smith, Lenora W: 8015
Smith, Lindsey: 3535, 8010, 8010, 8010, 8020
Smith, Louisa H: 4225
Smith, Matthew Lee: 1190, 7145, 8000, 8020
Smith, Patrick: 9010
Smith, Sarah K: 2530
Smith, Tracey: 7205
Smith Hinders, Julie: 7350
Smith Monroe, Suzanne C: 3380
Smith Slingerland, Kerri: 8010
Smithroat, Noemi: 3215
Smithwick, Megan: 7225
Smyles, Hanna: 7100
Smyre, Patricia M: 1315
Sneed, Rodlescia S: 1540, 2425, 4070, 7265, 7735
Snigurska, Urszula A: 8015
Snipe, Brandon: 4420
Snitz, Beth E: 7720, 9000
Snodgrass, Josh: 8015

Snow, A. Lynn: 1070, 1080, 1210, 3240, 7250, 7685
Snyder, Scott: 7680, 7725
So, Rina: 3125
Sodhi, Jaspreet K: 7300
Sodums, Devin J: 7040
Soerensen, Mette: 7010
Soke, Lisa A: 8015
Sokolova, Alexandra O: 7020, 7205
Sokolsky, Oleg: 7145
Sol, Ketlyne: 1045, 1495, 3400, 7535
Soldan, Anja: 2525, 3070, 3555, 7015
Sole, Jorge L: 7405
Solis-Herrera, Carolina: 1460
Solomon, Annie: 8010
Solomon, Philip: 8015
Solorzano, Emily: 3485, 7045
Solway, Erica: 1085
Somerville, Ceara: 7485
Sommers, Delaney D: 7150
Sommet, Nicolas: 4455, 7535
Son, Claudia: 1325
Son, Jung Yoen: 7435
Son, Rynnie Cin Ee: 8020
Sone, Chikako: 8015
Song, Guojie: 7605
Song, Huiru: 8010
Song, Jieun: 8000, 8020
Song, Lixin: 7535, 8015, 8050, 8085
Song, Max: 4220
Song, Minju: 3185, 7005
Song, Qian: 3495, 7235, 8015
Song, Rhayun: 7275, 7640
Song, Shulin: 8000
Song, Woojin: 8020
Song, Xiaowei: 7275
Song, Yeonsu: 2345, 7660, 7735
Song, Yixiao: 7640
Song, Yuting: 1245, 4195
Song, Yuxiao: 1130
Soni, Albin: 2410, 8010
Sonnega, Amanda: 3540, 7050, 7525, 8020
Sonnega, John R: 8020
Sonnenfeld, Mandi: 4340
Sonnentag, David: 4530
Sonney, Jennifer: 7465
Sopheab, Heng: 8015
Sörensen, Silvia: 3045, 4400, 7240, 8020
Sorra, Joann: 7545
Sosnowski, David: 4005
Sothers, Hanna: 8000
Sou, Ka Lon: 2100
Soulie, Brianna: 1010, 7160
Soumyanath, Amala: 7320
Sourbati, Maria: 2565
Sousa, Carlos: 8010
Sousa, Sarah: 2360
Southerland, Jodi L.: 8000, 8020
Souza, Anita M: 1400
Spangler, Hillary: 3585, 7275, 7535
Sparks, Sarah: 3385, 9015
Spartano, Nicole: 7500, 7930
Spears, Isaiah: 8010
Spears, Katie S: 3100, 4390, 7065
Speers, Alex: 7320
Sperber, Nina: 8015
Spetz, Joanne: 3105, 8055
Spilsbury, Karen: 2140, 4255, 7295, 7520
Spini, Dario: 4455, 7535
Spira, Adam P: 1255, 3070, 7615
Spiro, Avron: 2385
Spiro III, Avron: 4375
Spofford, Victoria: 8015
Spradley, Laura: 7650
Sprague, Briana N: 7625
Sprague, Karalin: 3490
Spring, Lauren: 2495, 7420
Springer, Jenna: 7735
springer, Shmuel: 1245, 2240
Springstein, Tabea: 3355
Squillace, Marie: 4305
Squires, Allison P: 7535
Sridhar, Aditya: 4005
Srikanth, Velandai: 4100
Srisuk, Wasana: 8015
Srivastava, Ankita: 7455
Srivastava, Sanjana: 4420
Srivastava, Shobhit: 7605
St. John, Phil: 7060
St. Louis, Renée M: 3455, 8020
Stahlmann, Melanie: 1220
Staley, Mark: 2490
Stallard, Eric: 1025
Stalter, Lily: 2190
Stam, Elisabeth J: 3065, 3530, 4270
Stamatakis, Emmanuel: 1245
Stamenkovic, Ceda: 8000
Stanger, Madeline: 3115
Stangl, Morgan: 4110, 7235, 7700
Stankovic, John A: 8020
Stanley, Jennifer T: 4000, 4295
Stanmore, Emma: 2530
Stansbury, Kim L: 3275
Stark, Susan: 4475, 7260, 7655, 8000, 8015
Staton, Ashley: 4510
Stavrakopolous, Dawn: 2370
Stawski, Robert S: 4280, 4410
Stearns, Melanie A: 1135, 1405, 3360, 7500
Stechuchak, Karen M: 2145
Steckenrider, Janie: 7345
Steege, Linsey M: 4075, 8015
Steele, Joel S: 3285
Stefanacci, Richard: 4535
Stefanucci, Jeanine: 3175
Steffens, David C: 2495
Stein, Gary L: 2225
Stein, Ryan G: 7720, 8015
Steinman, Audrey J: 2215
Steinman, Bernard A.: 1005, 7140
Steinman, Kenneth J: 1270, 4110
Steinman, Lesley: 1470, 2010
Steinmetz, Jaimie D: 7040
Stellman, Jeanne M: 2385
Stellman, Steven D: 2385
Stephan, Abigail T: 1185, 2245, 7735, 8015, 8020
Stephan, Yannick: 7350
Stephen, Kelly: 2145
Stephens, Caroline E: 4520
Stephens, Elizabeth: 8000
Stephens, Elizabeth B: 8000
Stephens, Jacquelyn: 3355
Stephens, Kevin: 8015
Stephenson, Jane: 8000, 8020
Stepniak, Catherine: 3165
Steptoe, Andrew: 2365, 3050, 3095, 4440, 4500, 7005
Sterling, Madeline: 8015
Stern, Julia: 1215
Stern, Yaakov: 2425
Sterns, Harvey L: 2250, 2440
Stessman, Jochanan: 7060
Stessman-Lande, Irit: 7060
Stetten, Nichole: 8010
Stevens, Alan B: 3340, 4130, 4340, 7680
Stevens, Erin: 2445
Stevens-Lapsley, Jennifer E: 1275, 2135, 7155
Steward, Andrew T: 8090
Stewart, Anne L: 2460
Stewart, Heather G: 7405
Stewart, Heaven: 8020
Stewart, Ian: 3260
Stewart, Meagan: 7070
Sthur, Nicole: 8000
Stickel, Ariana M: 7000, 7455, 7540
Still, Catherine: 1455, 4530, 7270
Stinchcombe, Arne: 7100
Stites, Shana: 1280, 8020
St-Martin, Genevieve: 7465
Stocks, Jane: 8020
Stockwell, Robert: 8000
Stodola, Allyson: 8010, 8020
Stoeckle, Rebecca J: 1260
Stoffel, Devon: 8060
Stojanovic, Marta: 7720
Stokes, Jeffrey: 1360, 2205, 3065, 3075, 3495, 3530, 3545, 4270, 7245, 8015
Stolle-Wahl, Claudia: 2140
Stoltenberg, Peggy: 3575
Stone, Alison: 8010
Stone, Katie L,: 1255, 2290
Stone, Kimberly: 7535
Stone, Robyn: 4265, 4395, 7135
Stone, Sarah M: 9020
Stone, Sasheen T.: 7425, 7500
Stone-Bury, Lisa E: 7115
Stout, Jeffrey R: 4420, 7205, 7640, 7735, 8015, 8020
Stout, Michael B: 4310
Stoyles, Sydnee: 7020, 7205
Stradford, Joy: 8015
Stradtman, Megan: 7205
Strand, Bjørn Heine: 2425, 7005
Strath, Larissa: 1445
Stratton, Lauren: 2105, 4200, 8010
Straughan, Paulin: 7050
Strayer, Andrea: 1455, 4530, 7270
Streed, Carl: 4225
Strickland-Hughes, Carla M: 7250, 8020
Strickland-Yanick, Leslie M: 8015
Stringer, Wilma: 3170
Stringfellow, Mary Kris: 8010
Stripling, Ashley: 7460
Strobach, Tilo: 4490
Strohl, Katyayani: 7530, 7905

Strommen, Jane: 7725
Strong, Jessica V: 7065, 7150
Strong, Randy: 4310
Strotmeyer, Elsa: 1090, 1410, 7060, 8015
Stroud, Jared: 8020
Strough, JoNell: 8000
Struble, Laura M: 7435
Stuart, Nicole S: 4405
Stubbs, Brianna J: 8000
Stuckenschneider, Tim: 8015
Studenski, Stephanie L: 2510
Stuifbergen, Alexa: 7145, 7435
Stump, Timothy: 4195
Sturdevant, Diana L: 2550
Sturmlechner, Ines: 3160
Stypinska, Justyna Monika: 2565, 3315
Su, Mengxuan: 7355
Su, Tai-Te: 7300
Su, Wei-Hsuan: 7010
Su, Yan: 2530, 8015
Su, Yan-Jhu: 1405, 4270, 7070, 7095, 7240, 7735, 8015
Suanet, Bianca: 2025
Suarez, Jethro Raphael M: 3055, 7620, 7640, 7720, 7735, 7750, 8015, 8020
Subbalakshmi, Koduvayur: 4180
Suberry, Assaf: 1190, 2030
Sucic, Joseph F: 7300
Suda, Hitoshi: 4135
Sudore, Rebecca L: 2255, 3175
Sue Mangalindan, Ruby: 8000
Suemoto, Claudia Kimie: 1320
Suen, Jonathan J: 1440
Suganuma, Ippei: 7420
Sugar, Judith A: 2250
Suggs, Andy: 7700
Sugimori, Haruka: 4050
Sugiyama, Mika: 7350, 7640
Suh, Heather: 2550, 7005, 7245
Suitor, J. Jill: 3165, 7265
Sukoff Rizzo, Stacey J: 8000
Suliburk, James: 2080
Sullivan, Barbara: 3340
Sullivan, Daniel: 8020
Sullivan, Darcy: 2500
Sullivan, Dennis: 7480, 8015
Sullivan, Jennifer L: 3425
Sullivan, Jennifer: 8020
Sullivan, Kevin J: 1000
Sullivan, Suzanne S: 7055, 7435, 7915
Sultana, Shabiha: 7285
Summapund, Jenny: 3340
Sun, Changxian: 7545, 7915
Sun, Eric: 2310
Sun, Fei: 1355, 2355, 3270, 8020
Sun, Haosen: 1405, 3410, 8025
Sun, Huaman: 7350
Sun, Kevin: 2190
Sun, Na: 1535, 4385, 8010
Sun, Nianzi: 8020
Sun, Nuo: 2165
Sun, Peter C: 8010
Sun, Shuimei: 7010
Sun, Tongxin: 7035, 7265, 7520,: 8010
Sun, Winnie: 3010, 7295, 8010
Sun, Xin: 3025, 7605
Sun, Ying: 8015
Sun, Yue: 2160, 7750
Sun, Yue Yang: 2030, 3480
Sunada, Junko: 7295
Sundby, Jonathan M: 8020
Sundstrom, Kim: 7630
Sung, Pildoo: 7145
Sunil, Surya: 8015
Sun-Mitchell, Shan: 1455, 4045
Supiano, Katherine P: 4520, 7425, 7640, 7905
Supiyev, Adil: 4535, 7535, 7540
Surachman, Agus: 1350
Suriaga, Armiel: 3460
Sussman, Tamara: 2560, 4195, 7055
Sutin, Angelina R.: 7245, 7350
Sutphin, Brandy: 3430
Sutphin, George L: 4005, 7010
Suzuki, Haruno: 7745
Suzuki, Hiroyuki: 1265, 7680
Suzuki, Lisa: 8085
Svec, Joseph: 1070, 1165, 1335, 4060, 7145
Svedberg, Pia: 2365
Sverdrup, Karen: 7005
Svitak, Aleta: 7455
Svynarenko, Radion: 3385
Swafford, Riley J: 8015
Swaim, Kathryn E: 4405
Swanson, Breanne M: 7350
Swanson, Helena: 2440
Swanson, Leslie M: 1365
Swanson, Sarah R: 7660
Swinkels, Joukje: 1120
Swint, Shelton: 8000
Sy, Maimouna: 4370
Syed, Ayesha: 2105
Syed, Orchee: 4500
Syed, Quratulain: 1200, 2345
Sylvers, Dominique: 7350
Syme, Maggie: 7355
Syros, Reiley: 4205
Szanton, Sarah L: 1365, 2010, 3150, 3500, 4420, 7445, 7935, 8000
Sze Him Isaac, Leung: 7490
Szerszen, Anita: 7025
Szmulewitz, Russell: 7205
Szymanski, Benjamin R: 7695
Szymanski, Gabrielle: 2480
Szymkowski, Amanda: 7480
Szypulski, Evan: 8010

**T**

T, Muhammad: 3505
Taani, Murad H: 7070, 7425, 7540, 7930
Taffet, George E: 1420, 2080, 2235, 3210, 7450, 8000
Taha, Hash Brown: 9000
Tahmaseb, Jasmin T: 8090
Tai, Daria: 7720, 8015
Tai, Joe: 1015
Takagi, Emiko: 7265
Takahashi, Tomoya: 1265
Takase, Mai: 7420, 8015
Takeda, Kazuya: 7270, 8000
Takeuchi, Masumi: 2470, 7360
Takeuchi, Satoko: 7105
Takeya, Yasushi: 7330, 8055
Talamantes, Michael: 7650
Talavera-Garza, Liza: 7425, 7935
Talmage, Alexis: 1160
Talton, Jennifer: 1410
Talwar, Sonia: 4200
Tam, Roger: 1150, 3335
Tambor, Ellen: 1260
Tamburri, Nicholas: 7525
Tan, Annabel: 8015
Tan, Brian: 7675
Tan, Cai Ning: 8015
Tan, Chia Jie: 7200
Tan, Edina YQ: 7205
Tan, Micah: 7050
Tan, Nasya S: 7140
Tan, Xiangchun: 7060
Tanaka, Tiffany Suchada: 2460
Tanaka, Toshiko: 4170
Tanbonliong, Gretchen: 3580, 7110
Tang, Fei: 7675
Tang, Fengyan: 1170, 1290, 1380, 3245, 3440, 4365, 8020, 8050
Tang, Foong Yee Vivien: 8010
Tang, Jennifer: 3200
Tang, Lingzi: 8000
Tang, Siyuan: 4055
Tang, Vera Mun Yu: 4050, 7640
Tang, Victoria: 4045
Tang, Weihong: 7010
Tang, Wen: 8015
Tang, Xiaotong: 2185
Tang, Xueting: 9000
Tang, Yimin: 2530
Tang, Yiting: 1560
Tangen, Gro Gujord: 7005
Tanglai, Wirampa: 1505
Taniguchi, Yu: 7275
Tanji, Tarin T: 7055
Tanley, Jordan: 3180
Tanner, Jeremy A: 4225
Tanner, Kamaryn: 7010
Tanner, Laura: 1320
Tanoue, Leslie: 7055
Tantipinichwong, Nicha: 7320
Tapola, Ella: 2460
Tappen, Ruth M: 3460, 7360, 7425, 7610
Tarabochia, Dawn: 7260, 8020
Tarantini, Stefano: 8015
Tarbox, Nathan: 2265
Tarpey, Thaddeus: 7465
Tarraf, Wassim: 1495, 2120, 7455, 7540, 7630, 8010, 9015, 9050
Tate, Alex: 7215
Tate, Channing E: 8020
Tate, Judith A: 7155, 7435, 7660
Tate, Rachel: 8055
Tate, Rio S: 8000
Tate, Robert: 7060
Tavares, Karina: 8015
Tawodzera, Obert: 3550
Tay, Djin: 4520
Tayade, Arti: 7155
Tayari Ashtiani, Reza: 3410
Taylor, Brent: 4055
Taylor, Christopher: 7200
Taylor, Corliss: 3150
Taylor, Harry: 1225, 2055, 3045, 3180, 4190
Taylor, Janelle S: 3065

Taylor, Janiece: 1365, 2230, 8000, 8015
Taylor, Jean O: 7425, 7465, 7905
Taylor, Miles G: 1280, 3540, 3555
Taylor, Morgan K: 1525, 7455, 7610
Taylor, Philip: 1080, 1360, 4290
Taylor, Tanya: 3240
Taylor, Zachary J: 1265
Taylor, Zachary E: 4410
Tchkonia, Tamara: 8000
Teachman, Gail: 7465, 7910
Teaster, Pamela B: 1200, 2275, 2495, 4135, 8010
Tebay, Jordan: 7440, 7500
Tedder, Jamie: 8015
Tee, Silvia: 7480, 7900
Teerawichitchainan, Bussarawan Puk: 4050
Teichman, Samantha: 2395
Teixeira, Laetitia: 7745
Tejada Arias, Karla: 7410, 7900
Teles, Soraia: 8015
Tell, Eileen J: 7490
Telonidis, Jacqueline: 4520, 8020
Templeman, Maureen E: 7065
Teng, Chiao-Hsin: 8035
Tennenbaum, Louis: 8020
Tennyson, Robert L: 7210, 8020
Teno, Joan: 3535
Tenquist, Miriam: 1450
Teodoro Maciel, Leticia: 7225, 7655
Teply, Abby L: 7440, 7490
Tepper, Lynn M: 8010
Terashima, Mikiko: 3170
Terhorst, Lauren: 7330
Terkpertey, Eric: 2220
Terracciano, Antonio: 7245, 7350
Terry, Christine: 1265
Terry, James Greg: 2220, 8000
Terveer, Katy: 1415, 8065
Tesar, Sarah: 8010
Teshome, Abraham Z: 7515, 7610
Testad, Ingelin: 8015
Tester, Griff: 8050
Tha, Soe Han A: 2520
Thacker, Ayush: 4370
Thalil, Muhammad: 1255, 7605, 8000
Thayer, Erin K: 7405, 8055
Thayer, Rachel: 7225, 7500
Theeke, Laurie A: 1465, 7350, 7540, 7910
Thiamwong, Ladda: 2300, 3055, 4315, 4420, 7025, 7200, 7205, 7260, 7620, 7640, 7655, 7720, 7735, 7750, 8000, 8015, 8020
Thibault, Emma: 7500, 7930
Thibodeaux, Brett: 8000
Thiel, Susie: 1480
Thierry, Amy D: 2330, 3000, 3180
Thin-Elk, Serene: 7425, 7500
Thingstad, Pernille: 7060
Thoma, Mary C: 8015
Thomas, Amanda S: 1095
Thomas, Amy: 3435, 7205
Thomas, Kali: 2155, 2185, 3535, 4460, 8010
Thomas, Karuna S: 8000
Thomas, Kelsey R: 7005, 8010, 8015
Thomas, Lisa: 7360
Thomas, Neil W D: 3285
Thomas, Nikita: 3150
Thomas, Zoe: 8020
Thomas Hebdon, Megan C: 4330
Thomas Tobin, Courtney S: 3475, 7540
Thomeer, Mieke B: 7225
Thompson, Alisha R: 3275
Thompson, Anya: 7150
Thompson, Atalie C: 1090, 2220
Thompson, Carl A: 4255
Thompson, Devin A: 8020
Thompson, Edward H: 7150
Thompson, Genevieve: 7055
Thompson, Hilaire: 4220, 7655
Thompson, Karl: 7425, 7930
Thompson, Katherine: 2340, 8020
Thompson, LaDora V: 8015
Thompson, Lena: 3005, 3345, 3485, 3585, 4015, 4320, 8070
Thompson, Louisa I: 4515, 7040
Thompson, Meghan: 2450
Thompson, Roy A: 1530, 7535
Thompson, Veronica: 9025
Thorne, Adrienna: 2265
Thorne, Christopher B: 3330
Thorpe, Carolyn: 4195
Thorpe, Joshua M.: 2180
Thorpe, Roland J: 2020, 2055, 3180, 3475, 4485, 7455, 7700, 1250, 2190, 4490
Thuemling, Teresa N: 2410, 4530
Thunell, Johanna: 2500
Thyagarajan, Bharat: 2460, 7010, 7060
Tian, Felicia F.: 1195
Tian, Junyan: 2330, 8000
Tian, Kun: 8010
Tian, Qu: 3070
Tian, Shubo: 1355
Tian, Teresa: 2510, 3330, 7090, 7720, 8000, 9015
Tian, Xin: 1090
Tian, Yuke: 1520
Tibbitts, Deanne: 7020
Tibirica, Lize: 3115
Tice, Abigail: 8010, 8015
Tiede, Ben: 8015
Tiemeier, Henning: 4550
Tien, Hsaio: 7275
Tien, Phyllis: 8010
Tillmann, Jacob: 7320
Timmerman, Christa: 3515
Tingvold, Laila: 1195
Tingvold, Maiken: 7610
Tintle, Nathan: 7615
Tipton, Mary E: 3485
Tiwari, Anjali: 7630
Tnesh, Basma B: 7530
To, Louis: 2150, 4365
Toba, Kenji: 7680
Tobón, Diana P: 2510
Tobey-Moore, Leah: 7325, 7650
Tobin, Karin: 2230
Tobyne, Sean: 7425, 7635, 7930
Tocchi, Christine: 1105
Toda, Eiji: 7410
Toh, Wei Xing: 7155
Toledo, Anna B: 8020
Toliver, Joy: 1500
Toll, Kara M: 4370
Tom, Sarah: 2425
Tomarevska, Olena S: 7505
Tomasini, Sergio Luiz V: 3155
Tompkins, Catherine J: 2415, 3510, 7270
Tong, Heling: 2240
Tong, Hongmei: 2050
Tong, Jialiang: 1355
Tong, Juncheng: 1215, 2030
Tong, Yao: 4225
Tong, Yuying: 3360
Tonkikh, Orly: 2200
Tonui, Betty: 7505
Toohey, Ann M: 3465
Topping, Michael S: 3225
Torke, Alexia: 4440, 7465
Toro-Serey, Claudio: 7425, 7930
Torres, Jacqueline M: 2020, 8015
Torres, Karen: 1480, 7200
Torres, Sandra: 1360, 3550
Torres, Stacy: 1140
Toseef, Mohammad Usama: 7530, 8010, 9050
Toshiko, Tanaka: 8000
Tosun, Duygu: 7005
Toth, Federico: 7515, 7915
Toto, Pam: 2190
Totten, Annette M: 4160
Touchinsky, Susan Martin: 7160
Towe, Maria: 8010
Towell, Michelle: 7750
Tower, Leslie: 8000
Towns, Juell: 3140
Townsend, Lisa: 8020
Towsley, Gail L: 1160, 2395
Toyama, Itsuko Kanamoto: 7505
Toyokawa, Noriko: 3560
Trabant, Marieke: 7075
Tracy, Brian L: 3115
Tracy, Eunjin: 3055
Tracy, Karen K: 8015
Traeger, Lara: 7680
Trainum, Katie: 7435
Tramujas Vasconcellos Neumann, Lycia: 3155, 8010
Tran, Dylan T: 3475
Tran, Melissa K: 1275, 2135, 7155
Tran, My Duyen L: 8095
Tran, Nathaniel M: 3075
Tran, Phoebe: 1060
Tran, Toan: 8020
Tran, Viet-Thi: 8010
Trane, Ralph Moeller: 3225
Trapido, Edward J. J: 8020
Traver, Anthony C: 1295, 2070, 2195
Travers, Jasmine: 8010, 8035, 8070
Travison, Thomas: 2040
Traywick, LaVona: 4540
Treat, Anny: 3255, 7510, 7545, 7915
Tremblay, Roma: 8015
Tremont, Geoffrey: 7630
Tretyakova, Natalia Y: 2165
Trevisan, Caterina: 3035
Trexberg, Kaitlin M: 7490

Trimble, Shawn X: 4370
Trimboli, Tracy: 3265
Trinitas, Joseph: 4080
Trinkaus, Eva-Maria: 2320, 4285
Trinquart, Ludovic: 7360
Triolo, Federico: 3035
Tripathi, Alok: 7285
Trittschuh, Emily H: 7675
Trivedi, Ranak: 2190, 2545, 3110, 7135
Trojanczyk, Carrie: 2335
Troost, Jonathan: 7065
Troosters, Thierry: 2170
Trost, Alexander: 4100
Trottier, Lise: 1100
Troutman, Brooke: 1105, 7265
Troutman-Jordan, Meredith: 1270
Trubenstein, Paige: 4095
Trubits, Em F: 7270
Trudeau, Scott A: 1005, 3395
Trujillo Tanner, Corinna M: 7035
Trumbo, Jami: 1440
Truskinovsky, Yulya: 7030
Tsai, Chih-TIng (Gina): 7300
Tsai, Feng-Jen: 7735
Tsai, Jenny Hsin-Chun: 1400
Tsai, Kai-Li: 7360
Tsai, Pao-Feng: 7275
Tschoe, Marianne: 7490
Tse, Dwight: 4020, 7725, 8020
Tseng, Chien-Yu: 1255
Tseng, Tung-Sung: 8020
Tseng, Winston: 7035
Tsimpida, Dialechti: 1170, 1360, 3195
Tsitsipatis, Dimitrios: 7305
Tsosie, Nathania: 2335
Tsotsoros, Cindy: 1495, 2130, 8015
Tsou, Hsin-Ying: 7055
Tsuchiya, Kazumi: 3180, 4190
Tsui, Iona: 1195
Tsui, Ronald: 4365
Tsukada, Noriko: 4135, 7295, 7405
Tsuker, Sophia: 2015, 3165, 7065
Tsukiashi, Motoki: 8015
Tsuzuki, Takamasa: 7315
Tsygankov, Denis: 8000
Tu, Wanzhu: 4195, 7465
Tu, Xueting: 4300
Tu, Yufang: 7055, 7150, 7360
Tucker, Brittany: 3270
Tucker, David: 8015
Tucker, Gretchen G: 1070, 2015, 4195
Tucker, Julia E: 1405, 4400
Tucker, She'Lon: 7435
Tucker-Roghi, Gina C: 2535, 3395
Tuckowski, Angela: 4005
Tuffier, Stéphane: 3125
Tuft, Samantha E: 1145, 7405, 7470
Tuladhar, Lirisha: 3055, 3565
Tulloh, Anna: 2260, 4345, 7725
Tumin, Dmitry: 7080
Tunalilar, Ozcan: 1230, 2185, 4510, 9045
Tunningley, Joan: 8010
Tuokko, Holly: 3185
Tupper, Emily: 1125, 7425
Tupper, Susan: 4195
Turcotte, Samuel: 8020
Turgeon, Jacques: 7425, 7630
Turiano, Nicholas A: 7070, 7115
Turkelson, Angela: 4525, 7660, 8020
Turner, Anne M: 7425, 7465, 7905
Turner, Chris: 8000
Turner, Grace: 3245
Turner, Jennifer R: 1525, 4280, 7255
Turner, Natalie: 3315, 8010
Turner, Pamela R: 3575
Turner, Rachael: 7065, 8020
Turner, Robert W: 1125
Turner, Seymour: 8020
Turner, Shelbie: 2305, 7270
Turner, Sidney: 3505
Tursi, Mark: 8015
Twardzik, Erica: 2525, 3070, 8065
Tyagi, Pranjal: 7360, 7730, 8010
Tyler, Emily R: 7545
Tzemah-Shahar, Roy: 1435, 7300, 7600, 7695, 9040
Tzeng, Huey-Ming: 2090
Tziarli, Dimitra: 8020

U

Ubaque, Lina Paola Buitrago: 8015
Uchida, Hayato: 7420
Uddin, Jalal: 3490
Udeh, Kingsley: 3475, 8010
Udenta, Mesomachi: 7000
Udo, Charles: 7700
Uehara, Tamiko: 4135
Ueki, Kaori: 8015
Ujano-De Motta, Lexus: 8010
Ukraintseva, Svetlana V: 1025, 7010, 7075, 7200, 7210, 7715
Ulitsa, Natalie: 7420
Umar, Tooba: 8015
Umberson, Debra: 1540
Umegaki, Hiroyuki: 7275
Umoh, Mfon: 2230
Undem, Teri: 7090, 7745
Unger, Kathryn: 7655
Unick, George: 8010
Unni, Vivek: 7310
Unroe, Kathleen: 1160, 4195, 7110, 8010, 8015, 9040
Unsworth, Julia: 8010
Upal, Shaneen: 7495
Upton, Rachel D: 7205
Ura, Chiaki: 7350, 7680
Urbanski, Dana: 1175, 3130
Urias, Uriel: 8010, 8015
Uribe, Alondra: 7700
Urrutia-Pujana, Leixuri: 7540
Usami, Satoshi: 7640
Utz, Rebecca: 3500, 4520, 7425, 7905

V

Vaccaro, Joan A: 2330
Vaddi, Prerana: 8000
Vafaei, Afshin: 2060, 7465, 7910
Vafiadis, Dorothea K: 3580, 7110
Vahedi, Vanessa: 7630, 7905
Valdivia, Nathaly: 1425
Vale, Michael T: 1010
Valenti, Korijna: 2225
Valentine-Graves, Mariah: 8015
Valenzano, Dario Riccardo: 3470, 4210
Vallejo, Rose: 8015
Valletta, Martina: 4315, 7010
Van, Mary: 7465, 7910
Van Boekel, Leonieke: 3425
Van Bogart, Karina: 4180, 4515
Van Camp, Bennett: 8000
Van den Block, Lieve: 2255
van den Broek-Altenburg,, Eline: 8000
van der Steen, Jenny T: 2255, 7640
van der Willik, Odette: 3045, 4210
van Dijk, Aileen: 8015
Van Eck, Richard: 8020
Van Gameren, Mateja: 1010
Van Haitsma, Kimberly: 1160
Van Houtven, Courtney H: 1475, 2090, 2180, 4530
Van Ooteghem, Karen: 2170
Van Orden, Kimberly A: 3110, 4120, 4180
Van Raamsdonk, Jeremy M: 8020
Van Scov, Lauren J: 1080
Van Son, Catherine R: 7155, 7465
Van Vleet, Bryce A: 7025, 7070, 7090
Van Vleet, Samuel: 7750, 8015
van Wijngaarden, Jeroen: 7445
van Zaalen, Yvonne: 3425
Vance, David E: 7285
VanCleef, Tricia R: 7625
Vandawalker, Melissa: 1015, 7420
Vanderboom, Catherine: 1475, 4415
Vanderlaan, Courtney: 1175
VanderWeele, Tyler: 1545, 4550
VanderWerf, Jess A: 2490, 7340
VandeVrede, Lawren A: 8070
VandeWeerd, Carla: 8015
Vang, Cindy: 4445, 7600
VanHaitsma, Kimberly: 1080
VanPutten, Rachel: 3110
Varadhan, Ravi: 1205, 7620
Vargas, Andrea-Paula R: 7720
Varley, Allyson: 3330
Varma, Hiren: 7200
Varma, Vijay R: 2045
Varshney, Ishan: 2340
Vasilevskis, Eduard E: 4530
Vasquez, Elizabeth: 3460, 8000, 8010
Vass, Emma L: 7000
Vasylenko, Maksim: 2325
Veeramah, Krishna: 8020
Vega, Irving E: 2020
Veilleux, Megan: 8020
Velazquez, Ana E: 7700
Velazquez, Enilda M: 8020

Velez-Ortiz, Daniel: 8020
Veliz, Philip: 7360
Velk, Lace J: 7440
Vellani, Shirin: 4255, 9040
Vellone, Ercole: 7245
Venditti, Elizabeth M: 7300
Venegas, Maria D: 7135
Venema, Dawn: 8015
Venkata, Pruthvinath Jeripity: 8015
Venkatesh, Arjun: 1130
Venna, Venugopal: 1460
Ventakesh, Manasa: 2190
Ventura, Juan D: 7700
Venugopal, Surya: 7310
Verbeek, Hilde: 2140, 4345
Verdery, Ashton: 4050
Verdin, Eric: 8000
Vereijken, Beatrix: 2170
Vergara, Kristine E: 7000
Vergenz, Laura: 7200
Vergez, Sasha: 4360
Verghese, Joe: 7750
Verma, Ambika: 7465, 8000
Verma, Mansi: 1535
Versey, H. Shellae: 1295
Verstaen, Alice: 7675
Vetrano, Davide Liborio: 3035, 3330, 4315, 7010, 7060
Vetter, Valentin M: 7030, 7210
Veturi, Yogasudha: 7455
Viceconte, Madeleine: 4205
Vichos, Theodoros: 8000
Vick, Judith: 2090, 2180, 4530
Vickburg, Jonathan: 2520
Victor, Christina R: 1225, 2055
Vidal, Jennifer Landeta: 2555
Videla, Emma: 7030
Vieira, Candice: 8085
Vieira, Edgar Ramos: 7610
Viel, Isabella C: 7720
Vig, Elizabeth K: 3065
Vijinski, Jon Parr: 7040
Villalba-Mora, Elena: 3150
Villar, Michelle: 7360
Villareal, Dennis T: 1330
Villemagne, Victor L: 3375
Vincent, Thomas: 4490
Vincent Duro, Marlon: 8020
Vinson, Latrice D: 1205
Virgin, Nicole: 8020
Visaria, Abhijit: 3290, 8015
Visser, Mandy: 2255
Visvanathan, Pallavi: 1140
Visvanathan, Renuka: 7275
Vivek, Sithara: 7010
Vivoda, Jonathon M: 3455
Vluggen, Stan: 8015
Vo, Quynh: 8010
Voegtline, Kristin: 2080
Vogelsang, Eric: 4400
Vogelsmeier, Amy A: 1530, 9025
Voils, Corrine I: 2190
Volek, Jeff: 8000
Volpi, Elena: 1275, 7400, 7920, 8015, 8015, 8050
Von Ah, Diane: 2445
von Bastian, Claudia C: 4490
von Gunten, Armin: 1220
von Hacht, Markus: 7260
Vonderheide, Clara: 2315
Vos, Carol M: 7435
Voss, Michelle: 7735
Voyich, Jovanka M: 7210
Vranceanu, Ana-Maria: 1345, 2315, 3250, 4370, 8000, 8020, 8025
Vrinceanu, Tudor: 4490
Vrlkjan, Brenda: 3185
Vu, Thi: 8020
Vu, Thi Hoang: 7245, 7270
Vyavahare, Sagar: 7210
Vytniorgu, Richard: 1015

**W**

Wada, Mineko: 7270, 8000
Wada, Tadashi: 4135
Waddell, Wendy: 7440
Waddington, Emily: 7465
Wadsworth, Sally J: 4095, 7225
Wagenknecht, Lynne: 1390, 7015
Wagg, Adrian: 8000
Wagle, Sampada: 4115, 8015
Wagner, Brandon: 7350
Wagner, Johanna: 8000
Wagner, Melanie: 2025
Wagner, Richard K: 8000
Wagner, Todd: 7080
Wagshul, Mark E: 1150
Wagster, Molly: 1340
Wahl, Anne L: 7640
Wahl, Hans-Werner: 1040, 1285, 1555, 7680
Waite, Elinor E: 7255
Waite, Linda J: 1110, 3410
Wakui, Tomoko: 7490, 7750, 8020
Waldinger, Robert J: 4375
Waldron, Danielle: 3530
Walker, Jenny A: 4390, 7065, 7200, 7205
Walker, Kealie J: 3035
Walker, Keenan: 1000, 7010, 7015
Walker, Teresa J: 7235, 7685
Walkner, Tammy J: 1395, 7665
Walkup, Michael P: 1390
Wall, Lucas A: 7440
Wallace, Brandy H: 7700
Wallace, Bruce: 3285
Wallace, Cara L: 4080, 7055, 7520
Wallace, Gail: 4490
Wallace, Meredith: 2290
Wallhagen, Margaret: 7140, 7745
Wallsworth, Christine: 2530, 7465, 7910
Walsemann, Katrina: 1280, 7040
Walser, Tracie: 3385
Walsh, Christine A: 2395, 2560
Walsh, Judith: 4230
Walsh, Maureen A: 7075
Walsh, Sarah: 8045
Walston, Jeremy D: 1205, 2570, 7305, 8000
Walters, Monica E: 1495, 2045, 9015
Walton, Allison: 8020
Walton, Mary: 1185
Waltz, Thomas J: 3110
Wan, Adrian H. Y.: 7725
Wan, Debby: 2150, 4365
Wan, Lu: 4315
Wan, Qiaoqin: 8015
Wan, Rachel: 7465
Wandee, Chanchanok: 8020
Wang, Adrienne M: 7075
Wang, Anni: 7245
Wang, Can: 8020
Wang, Chen-Pin: 7735
Wang, Chih-Hsuan: 7275
Wang, Claire L: 2145, 3200, 4010, 4320
Wang, Connor: 1130
Wang, Cuiling: 2160
Wang, Danny: 7425, 7615, 7930
Wang, Dingyue: 4450, 7270
Wang, Dongmin: 3050, 7680
Wang, Emily: 7535
Wang, Ethan B: 1440
Wang, Fei: 1060
Wang, Fei: 4310
Wang, Feng: 7010
Wang, Ge: 7285
Wang, Gene: 3285, 8040
Wang, Gwenneth: 4360
Wang, Hang: 3070
Wang, Haoqing: 4310
Wang, Haowei: 7140
Wang, Haowei: 8020
Wang, Helen: 7510
Wang, Hongning: 8020
Wang, Hsiu-Jung: 8010
Wang, Huijuan: 7670
Wang, Inga: 7205
Wang, Jia: 7640, 7690
Wang, Jiangxia: 7015
Wang, Jianye: 3350
Wang, Jing: 1380, 2000, 3305, 4030
Wang, Jing: 7315
Wang, Jing: 8010, 9000
Wang, Jing: 9030
Wang, Jingxuan: 1545
Wang, Jinjiao: 8035
Wang, Jiuzhou: 4430
Wang, Jouchieh: 7040
Wang, Julian: 7200, 8015, 9015
Wang, Junqiao: 9000
Wang, Juwen: 4455
Wang, Kaipeng: 4115, 7055, 8020
Wang, Katherine: 1380
Wang, Kuan-Yuan (Ken): 8015
Wang, Kun: 3030, 7155, 8010
Wang, Libin: 7010
Wang, Liru: 7355
Wang, Lu: 4220
Wang, Mei-Chen: 8010
Wang, Mengru: 3510
Wang, Mengya: 2060
Wang, Moyao: 8015
Wang, Mu-Cyun: 7080
Wang, Nan: 7275
Wang, Natalie: 4300
Wang, Ning: 4180
Wang, Peng: 8020
Wang, Qinchuan: 7305
Wang, Qingwei: 7490, 7515, 7925, 8095, 9045
Wang, Qun: 7625
Wang, Rui: 7280
Wang, Ruifeng: 7620
Wang, Shanshan: 7490
Wang, Shuangshuang: 7270
Wang, Shuo: 7010
Wang, Shuomin: 1235, 2255, 2465, 4055
Wang, Siqi: 4470
WANG, Siqiang: 8075
Wang, Siqing: 8010
Wang, Suyi: 7355

Wang, Tianhao: 1235
Wang, Weijun: 7245
Wang, Wenjin: 7040, 8020
Wang, Xi: 7540
Wang, Xia: 3270
Wang, Xiao (Joyce): 3535
Wang, Xiaochuan: 7105
Wang, Xiaomeng: 7235
Wang, Xin: 7610
Wang, Xuan: 7460, 7715
Wang, Xuedong: 8015
Wang, Xuzhi: 7360, 8015
Wang, Yan: 1445
Wang, Yanxin: 4055
Wang, Yanyan: 1090
Wang, Yaping: 7350
Wang, Yi: 4110, 7235, 7445
Wang, Yi: 7630
Wang, Yi: 7700
Wang, Yilin: 1355
Wang, Ying: 7420, 7920
Wang, Yiqi: 3480
Wang, Yixuan: 7055
Wang, Youhua: 8000
Wang, Yue: 3050
Wang, Yueting: 1485, 7145
Wang, Zheng: 4245
Wang, Zhi-wen: 2160, 4070, 7235, 7355, 7750
Wang, Zhong: 8090
Wang, Zih-Ling: 2090, 2115, 2450, 7465
Wanigatunga, Amal A: 2035, 3070, 8015
Wanigatunga, Sarah K: 3070
Wanlass, Jase: 7035
Ward, Angela: 3100
Ward, Chesney J: 7745
Ward, Heather A: 4440
Ward, Mark: 1290
Ward, Rachel: 3335
Warne, Donald K.: 7425, 7500
Warner, Lisa M: 2520
Warner, Lynn: 1465
Warren, Connor: 9025
Warren, John R: 1095, 4035, 4375, 7230, 7350
Warren, Teresa S: 7015
Warren-Walker, Alina: 8015
Warry, Wayne: 2335, 2395
Washburn, Stephanie: 8000
Washington, Ashley: 7135
Washington, Karla T: 1165, 2085, 4080, 4220, 7270, 7490, 7665, 8000, 8010
Washington, Tiffany R: 4175
Washington, Tracey: 1080
Wasik, Sophia: 2520
Watanabe, Kengo: 8000
Watanabe, Kentaro: 7750
Watanabe, Yuichi: 3200, 4190
Watanabe, Yuki: 7420
Waterhouse, Jodi L: 1415, 3465, 8020
Waters, Leland: 3510, 4290, 4470
Watkins, Michele: 1295, 4265
Watman, Deborah: 3105, 4480, 8010
Watts, Amber: 4315
Waugh, Kellie: 1260
Weathers, Brandy: 8000, 8000
Weaver, Ashley A: 2505
Weaver, France: 8045
Weaver, Raven H: 1190, 2180, 2295, 2325, 7350, 7530
Webb, Fern J: 2330
Webb, Jason: 7020
Webb, Sara: 7685, 8015
Webel, Allison: 2450
Weber, Daniela: 7315
Weber, Emma: 7660, 8020
Weber, Madeline K: 7205
Webster, Amy: 2145, 8015
Webster, Melinda J: 4130, 4340
Webster, Micah T: 8000
Webster, Noah J: 1045, 1495, 2025, 3040, 7720, 9000
Weech-Maldonado, Robert: 1425, 1550, 3255
Weese, Richelle: 7040
Wehage, Mattea: 7050
Wehry, Susan: 8010, 8015
Wei, Amy: 2205
Wei, Changning: 7625, 7710
Wei, Duo Helen: 3515, 7425, 7905
Wei, Hui: 2465
Wei, Jeanne Y: 7010, 7210, 7465, 8000, 8015
Wei, Kai: 7010, 7235
Wei, Wenxing: 1060, 7530
Wei, Yehua Dennis: 7420
Weil, Joyce: 1015
Weimar, Lisa S: 7735
Weinberg, Gil: 8010
Weiner, Bryan: 8010
Weir, David R: 2515
Weiser, Sheri: 8020
Weiskittle, Rachel: 7510, 7640, 8020
Weiss, Charlotte R: 1290, 7435
Weiss, Jenna: 4165
Weiss, Jordan: 1575
Weiss, Samantha: 9030
Weissberger, Gali H: 2001, 7495, 7930
Weith, Joanne: 7480, 7900
Weitman, Cassidy: 7030
Weitzel, Kelsey J: 3575
Weiyun, Chen: 7435
Wek, Aleksandra: 2085
Weldrick, Rachel: 2395, 2560
Welhaf, Matthew S: 4515
Wells, Jenna L: 3355, 8020, 8040
Welsh-Bohmer, Kathleen A: 3280
Wen, Chiung-Jung: 7275
Wen, Juncheng: 7035
Wen, Ming: 7230, 7420
Wen, Wan: 2265, 4420, 7505, 7720
Wen, Xi: 9030
Wende, Marilyn: 1045
Wendel-Hummell, Carrie L: 2500
Weng, Hui-Ching: 7460, 8000
Wenhan, Zhang: 1475
Wenzel, Arlett: 7110
Werbeloff, Merle: 2060
Werner, Nicole E: 1455, 2085, 2410, 4370, 4530, 7005, 7135, 7270, 8020
West, Jessica S: 7625
Westcott, James S: 1415
Westerlind, Bjorn: 8015
Westlake, Frances: 2135
Westmore, Megan: 2260, 4345, 7725
Westra, Jordan: 7405
Wethington, Elaine: 3040
Wettstein, Gal: 2420
Wettstein, Markus: 1375, 4065, 4500, 7035
Weuve, Jennifer: 4380
Weyrauch, Karen: 7325
Wezeman, Jackson: 7075, 8000
Whaikid, Phatcharaphon: 8015
Whalen, Stacey: 8010
Wharton, Whitney: 2225
Wheaton, Felicia V: 3235
Wheeler, Pariya: 7285
Wherry, Sarah J: 2345
Whetung, Cliff: 2335, 2470
Whitaker, Janice: 3380
Whitaker, Kara: 7735
Whitbourne, Susan Krauss: 3310, 3465
White, Bess: 7205
White, Carole L: 2295
White, Christian: 8010
White, Julie: 8010
White, Katie: 2070
White, Kina: 2480, 3100, 7240
White, Lane: 8010
White, Laura: 8010
White, Lillian A: 7005
White, Lindsay LY: 7000
White, Rebecca E: 3060
White, Stephanie: 8040
Whiteside, Phoebe G: 9025
Whitfield, Keith E.: 2190, 7455
Whitman, Kathrine: 8000, 8020
Whitmer, Rachel: 7535
Whitmore, Kim: 7660
Whitney, Robin: 2200, 8010
Whitson, Heather: 8015, 8020
Whitten, Justin M: 7260, 8020
Wicklein Sanchez, Heather: 7415, 7900
Wickline, Sierra: 9030
Wickson-Griffiths, Abigail: 4195, 7055
Wickwire, Emerson M: 1255
Widener, Michael: 4430
Widjaja, Nathan: 1095
Wiedrick, Jack: 7310
Wiemers, Emily: 4050, 7030
Wiersma Strauss, Anna: 4050
Wiese, Lisa A: 2360, 7200, 7260
Wiggins, Arika: 7440, 7935
Wihry, David C: 8010, 8015
Wijk, Helle: 4010
Wilber, Kathleen: 2150, 2375, 3525, 4150, 7725, 8010
Wild, Ellen: 4415
Wild, Tess: 8020
Wilder, Jocelyn: 4505
Wilder, Jocelyn R: 9020
Wildschut, Tim: 7255
Wiley, Christopher: 2165
Wilgenburg, Brian: 7210
Wilk, Adam: 7530
Wilkie, Rachel Z: 3245
Wilkins, James M: 3135
Wilkinson, Lindsay R: 8000
Wilks, Hannah: 4515
Wilks, Scott E: 3275
Willard, Meredith A: 7070
Willcox, Bradley: 1385, 8000

Willgerodt, Mayumi: 4470
Williams, Adrian: 7070
Williams, Amanda L: 8070
Williams, Brie: 3420, 7535
Williams, Cara: 8010
Williams, Cedric: 8020
Williams, Cierra: 8015, 8020
Williams, Claire M: 7725, 8010
Williams, Crystal Warren: 8070
Williams, Dylan: 7005
Williams, Ginger: 3575
Williams, Ishan: 7005
Williams, Ivan: 4050
Williams, Kelvin L: 1315
Williams, Kirk B: 7205
Williams, Kristine N: 1530, 4475, 7635, 7650
Williams, Micah: 8000
Williams, Mikaela: 2395
Williams, Shellie: 7250
Williams, Victoria J: 3225
Williamson, Christin: 1415
Williamson, Jeff D: 1090
Williamson, Marsha: 8015
Williman, Marty: 8020
Willis, Crysta: 1265
Willis, Matthew L: 7225
Willis, Sherry L: 4490
Willroth, Emily C: 7745
Wills, Celia E: 2415
Wilson, Annalee: 7650
Wilson, Chandra: 3100
Wilson, Deborah: 2010
Wilson, Jeff: 8000
Wilson, Kimberley J: 8010
Wilson, Sanjaya: 8010
Wilson, Stephanie J: 8020
Wilson, Tracey: 8015
Wilson, Traci L: 2070, 7465, 7700
Winberry, Joseph W: 3075, 8010
Windham, B. Gwen: 1000, 2525
Winesberry, Nehemiah Jude: 2460
Wing, Rena R: 1390
Wingo, Anna: 7055
Wingood, Mariana: 7720
Wininger, Claire: 8020
Winship, Jodi M: 4205
Winstead, Vicki: 1510
Winters, Emily D: 7005
Winters-Stone, Kerri M: 7020, 7205
Wirth, Maria: 1375, 4020
Wise, Jenni: 8020
Wisnivesky, Juan: 7630
Wister, Andrew: 1100, 4445, 7155, 7445, 7720, 7740, 8045
Witmeier, Kelsey N.: 8020
Wittenberg, Eve: 7275, 7280
Wittenberg, Grace F: 8010
Wittenberg, Nir: 8020
Wittich, Walter: 4300, 7635
Wittie, Michael: 8030
Witzel, Dakota D: 1525, 2385, 4280, 4405, 4410
Wladkowski, Stephanie P: 4080, 7055, 7520
Woehrmann, Anne: 7050
Wohns, Nicolai: 4285
Wojczynski, Mary K: 1025, 7010, 7060
Wolf, Jack: 4245
Wolf, Karsten D: 2140
Wolf, Michael S: 4230, 4480
Wolfe, Joseph D: 7225
Wolff, Jennifer L: 2085, 2230, 3570, 4300
Wolf-Ostermann, Karin: 2140, 4345
Wolfson, Christina: 7030
Wolinsky, Steven: 7285
Wolk, Megan W: 8010, 8020
Womack, Dana: 7440
Won, Chang Won: 8000, 8010, 8015, 8015
Won, Sarah: 8000
Wong, Alice C.: 8050
Wong, Alina Clarise: 2100
Wong, Chelsea: 4225, 8015
Wong, Dipsy H. S.: 8020
Wong, Edwin: 8010
Wong, Jason: 2120, 3360, 3410, 3480, 4505
Wong, Joey: 2530, 3205, 7465, 7750, 7910, 8010
Wong, Ka Kit: 7540
Wong, Karen: 7510
Wong, Karen Lok Yi: 1115, 7465, 7910, 8010
Wong, Kayla: 7085
Wong, Lily: 2410, 2530, 7465, 7750, 7910
Wong, Loong Mun: 3545
Wong, Meghan: 8000
Wong, Rebeca: 1320, 4500, 7140, 7235
Wong, Roger: 1425, 2185, 9015
Wong, Sarah: 8015
Wong, Yun Leng: 1485, 7145
Wong, Yunjie: 1015, 2185
Woo, Seoyoon: 7440
Wood, Julie K: 7325, 8015
Wood, Kristie A: 7270
Wood, Leila: 1510, 4355, 7405
Wood, Michele M: 7000
Wood, Stacey: 1370
Woodhouse, Mark: 1390, 1520
Woods, Ashley: 8015
Woodward, Amanda T: 8010
Woodworth, Emily: 1345
Woolever, David: 7635
Woolson, Sandra: 2180, 8015
Worschech, Florian: 4490
Worthington, Amber K: 7480
Wouters, Eveline JM: 3425
Wright, Bernadette: 8010
Wright, Eric R: 8020
Wright, Kathy D: 2415, 2550, 7490, 7660, 7935, 8020
Wright, Leslie A: 1415
Wright, Matthew R: 3065
Wright, Molly A: 7210, 7455
Wright, Morgan M: 2225, 3490
Wright, Rebecca: 7055
Wright, Regina: 2330
Wrigley-Field, Elizabeth: 4035
Wu, Barry: 7410, 7900
Wu, Bei: 1380, 2095, 2205, 3305, 3505, 3540, 4030, 7535, 7630, 8000, 8010, 8025, 9000, 9030
Wu, Chao-Yi: 8000
Wu, Chenkai: 4005, 4055, 7275
Wu, Deqing: 7715
Wu, Dongmei: 7235
Wu, Haorui: 3585
Wu, Heng: 7275
Wu, Horng-Shiuann: 8000
Wu, Huiping: 1355
Wu, Jiajun: 1575, 4420
Wu, Jia-Rong: 3320
Wu, Jingchuan: 8020
Wu, Jingxuan: 7725, 7745
Wu, Judy Z: 7275
Wu, Junhui: 7430
Wu, Kaili: 7470, 7905
Wu, Kevin Chien-Chang: 7460
Wu, Kuan-Ching: 7465, 8015
Wu, Mingche: 2085
Wu, MJ Mingche: 4300
Wu, Peter C: 7305
Wu, Rachel: 7135
Wu, Serena: 7525
Wu, Shaohui: 4360
Wu, Sherry: 1470
Wu, Shih-Cyuan: 7510, 7525, 7915
Wu, Tiffany: 8015
Wu, Xucheng: 8015
Wu, Yafei: 2255
Wu, Yanyan: 8000
Wu, YingHua: 8000
Wu, Yingyan: 4380
Wu, Yiwen: 4020
Wu, Yuxia: 3270, 7740
Wu, Zixuan: 7490, 7925, 8095, 9045
Wunderlich, Tiffany: 3485, 7685
Wyatt, Riley: 8020
Wylie, Molly: 7135
Wyman, Mary F: 2190
Wyte-Lake, Tamar: 3485, 4160, 7045

### X

Xaio, Xaviera: 2190
Xi, Yueming: 1405
Xia, Lu: 7305
Xia, Nan: 7080
Xia, Weiyi R: 4485
Xia, Wenran: 7445
Xia, Xiaohu: 8000
Xia, Xin: 1090
Xiang, Xiaoling: 2410, 4115
Xiao, Chunhong: 4045, 7285, 8000, 8010
Xiao, Huimin: 7670
Xiao, Michelle: 8015
Xiao, Yexuan: 7235, 7630
Xicota, Laura Vila: 1025
Xie, Bo: 1355, 2245, 2355
Xie, Jinchen: 2125
Xie, Kathy: 2220
Xie, Rui: 2300, 4420, 7025, 7200, 7205, 7260, 7620, 7655, 7720, 8015, 8020
Xie, Serena Jinchen: 2125
Xie, Wenyi: 3585, 7535
Xie, Yuqing: 1090
Xie, Zongxing: 3415
Xing, Yurong: 7545, 7915
Xiong, Xueying: 7035

Xiong, Yuan: 7345
Xu, Chunjuan: 1230
Xu, Emily L: 3105
Xu, Hanzhang: 2205, 3505, 7205, 7625, 8015
Xu, Jiahui: 7350
Xu, Jianhong: 4005, 4055, 7275
Xu, Jianhui (Frank): 4460
Xu, Jingjing: 2125
Xu, Kaidi: 7620
Xu, Ling: 1105, 2050, 7005, 7265, 8010
Xu, Lucy: 2310
Xu, Mengjiao: 1245
Xu, Mingming: 1475
Xu, Qin: 8000
Xu, Qingwen: 1115, 7540
Xu, Richard: 8000
Xu, Shicheng: 8020
Xu, Shu: 7140, 8015, 8020
Xu, Sophie Zhijing: 8010, 9030
Xu, Ting: 8000
Xu, Wenqian: 7430
Xu, Xiao: 7265
Xu, Ying: 7235, 7610
Xu, Yvonne Yiru: 7040
Xu, Ziyao: 1535
Xu, Ziying: 4170
Xue, Dandan: 7005
Xue, Qian-Li: 1205, 2010, 2465, 3150, 8015
Xue, Tingzhong (Michelle): 2205
Xue, Wei- Lin: 7405

### Y

Yabluchanskiy, Andriy: 8015
Yadav, Hariom: 3470, 7210, 8000, 8015
Yadav, Shweta: 8000
Yaffe, Kristine: 2220, 8015
Yahirun, Jenjira: 8010
Yaisikana, Mo'e: 7540
Yakong, Vida N: 4175
Yam, Hawking: 3130
Yamada, Cody: 2225
Yamada, Yuji: 7260
Yamaguchi, Satoshi: 7640
Yamaguchi, Yuko: 8015
Yamahana, Reiko: 7295
yamakawa, Miyae: 8055
Yamamoto, Hiroshi: 7400, 7920
Yamamoto, Ryohei: 7080, 7105
Yamamoto, Yasuhiro: 8015
Yamashita, Takashi: 1505, 1535, 2470, 3035, 7350, 7475
Yan, Changmin: 4205
Yan, Elsie: 2150, 4365, 7530, 8020
Yan, Jinhua: 7075
Yan, Mengzhao: 1005, 3385, 7725, 8010
Yan, Shibin: 1015
Yan, Xiting: 4170
Yan, Yuanjiao: 7450, 7930
Yan, Zi: 3025
Yancura, Loriena A: 8020
Yang, Annabelle R: 8010
Yang, Betsy: 2345, 7480, 7900
Yang, Chengfengyi: 8015
Yang, Chih-Hsiang: 8000
Yang, Chun-Ting: 3135
Yang, Eunjin: 7750, 8020
Yang, Frances M: 4475, 7650
Yang, Huili: 1235, 7440
Yang, Iris S: 8020
Yang, Jen-Hao: 7305
Yang, Kaite: 7235
Yang, KwangSoo: 7360, 7425
Yang, Liu: 7330
Yang, Mia: 3215
Yang, Michelle: 7400, 7920, 8015
Yang, Minhee: 7655, 7715
Yang, Po-Lung: 7080
Yang, Qing: 7625
Yang, Seonkyeong: 2145
Yang, Shuhui: 7425, 7930
Yang, Siqi: 4115, 7660, 8015
Yang, Wei: 1475
Yang, Xiali: 2500
Yang, Xiaoping: 7670
Yang, Xuebing: 8015
Yang, Yang: 1395, 3570, 8070
Yang, Yanping: 7010
Yang, Yijian: 4195, 8015
Yang, Yingqi: 8000
Yang, You Lee: 3370
Yang, Yuanyuan: 8010
Yang, Yue: 8015
Yang, Yulin: 2020, 7225
Yang, Yuxuan: 7335
Yang, Zhen: 7075
Yang, Zhongfang: 3505, 8010
Yang, Ziying: 7040
Yank, Veronica: 4045
Yankeelov, Pamela: 4470, 7340, 7730
Yanover, Chen: 8015
Yao, Kaichen: 8010
Yao, Li: 7505, 7910
Yao, Shanshan: 7710, 8000
Yao, Yao: 7300, 8015
Yarrarapu, Siva Naga Srinivas: 8010
Yasar, Sevil: 4300
Yashin, Anatoliy: 1025, 7075, 7200, 7210, 7300, 7715
Yashkin, Arseniy: 3010, 7075, 7200, 7300, 7620
Yasin, Reham: 7275
Yassine, Hussein: 8020
Yasumoto, Saori: 2470, 8020, 8020
Yauch, John: 7510, 7915
Yauk, Jessica: 2370, 3365, 7435
Ye, Fan: 3265, 3415
Ye, Haiger: 2565
Ye, Jing: 4435
Ye, Minzhi: 3360
Ye, Qiaofeng: 7010
Ye, Yan: 7280
Yedavalli, Anjali: 7270
Yee, Claire I: 3285
Yeh, Jarmin: 8025
Yen, Pei-Shan: 7080
Yenko, Ira: 1325
Yentes, Jennifer: 8000
Yeo, Audrey: 8015
Yeo, Hyesu: 7545
Yeom, Hyun-E: 7265, 8000, 8010, 8015
Yeoman, Carl J: 7210
Yeong, Joe: 7010
Yeung, Dannii Y.: 2520, 4390, 7745, 8000
Yeung, Lydia: 8015
Yi, Grace: 8000
Yi, Mo: 4070
Yi, Stella S: 8095
Yi, Zhiqi: 8020
Yigit, Oykum E: 7160
Yildiz, Mustafa: 1510, 2215, 7040
Yim, Cynthia: 8075
Yin, Cheng: 3085
Yin, Jiamin: 7030
Yin, Jianing: 7520
Yin, Lijuan: 8020
Yin, Michael: 8010
Yin, Qingqing: 7300
Yin, Xuecheng: 3165
Yin, Zenong: 8050
Ying, Gelan Lana: 2020
Ylitalo, Kelly R: 1365, 7300, 7720
Yockey, Robert: 7080
Yoder, Maria: 2140
Yokoyama, Sae: 8020
Yokoyama, Yuri: 7275, 7695, 8015
Yon, Yongjie: 1195
Yonashiro-Cho, Jeanine: 2001, 2375, 7405
Yoneda, Tomiko: 1570, 7205
Yoo, Aejung: 3545
Yoo, Haeun: 8000
Yoo, Leeho: 7005
Yook, Geun Sil: 8010
Yoon, Caroline: 2500
Yoon, Dokyung: 8000
Yoon, Jieun: 8015
Yoon, Jong Sung: 2360
Yoon, Joonsik: 8000
Yoon, Seolah: 7275, 8015, 8015
Yoquinto, Lucas: 3145
Yorgason, Jeremy: 2385, 7035
Yoshida, Hiroto: 7680
Yoshikawa, Aya: 8000
Yoshimoto, Kukuna: 4015
You, Jin: 3480
Young, Colin: 4235
Young, Dominic: 7265
Young, Heather M: 2200, 8010
Young, Jessica: 7320
Young, Khristina: 8000
Young, Lisa: 1530
Young, Meghan: 3380
Young, Vanessa: 7735
Young, Yuchi: 3255, 7055, 7460, 7925
Yount, Naomi: 7545
Yous, Marie-Lee: 7510
Yousefzadeh, Matt: 3160
Yow, W.Quin: 1405, 2100, 7455, 7925
Yu, Chang: 7225
Yu, Chen-Wei: 3355
Yu, Chin Wing: 7300
Yu, Doris Sau Fung: 1560, 7005, 7040, 8015
Yu, Fang: 7015, 7450, 8020, 8035
Yu, Heshuo: 7055
Yu, Hsiao-Wei: 7525
Yu, Jiao: 2020, 4430, 7630, 8055
Yu, Kexin: 7005, 8000
Yu, Oliver Hang Kin: 8020
Yu, Wanting: 2040, 4215, 4535
Yu, Wenshan: 7335, 7525
Yu, Xinfang: 3405
Yu, Xuexin: 1290, 2515, 9000

Yu, Yue: 7630
Yu, Zhan: 1115, 7540
Yuan, Jialin: 7670
Yuan, Rong: 8000
Yuan, Yaqun: 4440, 7060, 7090, 7630
Yuan, Yin: 7275, 7710
Yuan, Yiyang: 7275, 8015
Yuan, Yue: 3165
Yue, Hao: 4535
Yue, Yiwei: 1255, 3070
Yuelling, Thomas J: 8020
Yueter, Sheila: 7200
Yuh, Ahhyun: 7145
Yun, Hyunkyung: 1115, 1230, 8075
Yun, Seon Kyung: 8010
Yunfan, Wu: 7330
Yung, Hiu Kwan Esther: 8075
Yurekli, Hulya: 7160
Yurkiv, Halyna: 4270
Yus, Roberto: 1070
Yusuf, Khalid: 8010
Yuwen, Weichao: 2125

Z

Zabielaite, Meda: 8000
Zaborenko, Callie: 4455
Zaccaria, Daniele: 1220
Zadok, Erez: 3265
Zafar, Anum: 1380, 4485
Zaguri-Greener, Dalit: 4045
Zahodne, Laura: 1045, 1290, 1495, 2045, 3040, 7535, 8025
Zakrajsek, Jennifer S: 3455, 8020
Zaller, Nickolas: 3420
Zaman, Eman: 7105
Zamarripa, Estefania: 8015
Zambrano Garza, Elizabeth: 7535, 8020
Zammitti, Melissa G: 1020, 7630
Zampino, Marta: 8015
Zamrini, Edward: 2160
Zan, Hua: 8020
Zanasi, Francesca: 3025
Zang, Emma: 2120, 2305, 4505, 7485
Zanier, Nicole: 3455, 8020
Zaninotto, Paola: 2365, 2460, 3125
Zanjani, Faika: 3565, 8000
Zúñiga, Franziska: 4255
Zarit, Steven H: 3525, 4010, 4165, 4335, 7005
Zaritsky, Rebecca: 4485
Zarzaur, Ben L: 3335
Zaslavsky, Oleg: 2530, 7465, 8015
Zauszniewski, Jaclene A: 7265, 7660
Zavala, Daisy V: 4185, 8020
Zea, Miranda: 4130, 7270, 7660
Zecevic, Aleksandra A: 1050, 7200
Zei, Paul: 3330
Zeinali, Nahid: 8040
Zeitler, Katie: 7465, 7905
Zeki Al Hazzouri, Adina: 1545
Zelinski, Elizabeth: 7460, 8000, 8020
Zell, Angela: 7105, 7640
Zeng, Derong: 7200, 8010
Zeng, Shengyin: 7080
Zeng, Xiaoyi: 7655
Zeng, Ziwei: 4195
Zettel-Watson, Laura: 7475
Zhai, Xiangyu: 1560, 8015
Zhan, Rongfang: 8080
Zhan, Shiyin: 8015
Zhang, Anna Yan: 7460
Zhang, Bo: 1570
Zhang, Cuntai: 3350, 7075, 7080
Zhang, Haihong: 1235, 3330
Zhang, Hengyuan: 7080, 7420
Zhang, Jiaan: 8000, 8020
Zhang, Jianguo: 7080
Zhang, Jiawei: 3125
Zhang, Jing: 3325
Zhang, Jing: 7280
Zhang, Jingwen: 1450
Zhang, Meina: 7735
Zhang, Meng: 3245, 7695, 7935
Zhang, Nan: 8010
Zhang, Peiling: 7425, 7930
Zhang, Peiyuan: 4195, 7020, 7055
Zhang, Pengyuan (Kelvin): 2515, 4380
Zhang, Qingqing: 3330
Zhang, Qiong: 7035, 7265
Zhang, Ruiqi: 2255
Zhang, Ruohan: 1575, 4420
Zhang, Shenghao: 4420
Zhang, Shiyang: 7540, 8020
Zhang, Shuo: 4030
Zhang, Sifeng: 7420
Zhang, Sixun: 3330
Zhang, Ting: 7515
Zhang, Wei: 3305, 8015
Zhang, Wencheng: 4455, 7535
Zhang, Wenjun: 4030
Zhang, Wenyu: 7410
Zhang, Wuyang: 2035, 7140
Zhang, Xiaomin: 7010, 7210, 7465, 8000
Zhang, Xin: 7605, 8000
Zhang, Xing: 2515
Zhang, Xiping: 8000
Zhang, Xuefeng: 8000
Zhang, Xuemin: 3270
Zhang, Yalu: 8010
Zhang, Yan: 7080
Zhang, Yanrong: 2465
Zhang, Yaqing: 7430
Zhang, Yichen: 8000
Zhang, Yichi: 3135
Zhang, Yiming: 7320
Zhang, Youjuan: 8095
Zhang, Yuan S: 2515, 4380, 4445
Zhang, Yuankai: 7360, 8015
Zhang, Yuchen: 7200
Zhang, Yufeng: 4535
Zhang, Yukai: 2030, 3480
Zhang, Yuping: 7425, 7930
Zhang, Zhang: 9030
Zhang, Zhe: 7420
Zhang, Zhenmei: 3270
Zhang, Zhenxiang: 3330
Zhang, Zheyuan: 7720
Zhang, Ziwei: 3490
Zhang, Ziying: 4345
Zhang, Ziying: 7040
Zhao, Dan: 8000, 8010
Zhao, Fengzhi: 7490, 7925
Zhao, Hui: 2460, 7065
Zhao, Jin: 3270
Zhao, Karrie: 8015
Zhao, Lirong: 8000
Zhao, Mengke: 3360
Zhao, Qianhua: 9000
Zhao, Shangang: 7000
Zhao, Shibei: 7110, 7420
Zhao, Wei: 1580
Zhao, Xinyi: 4115
Zhao, Yong: 7750, 8015
Zhao, Yuanchang: 3385
Zhen, Zhihong: 3505
Zheng, Anqing: 7225, 8020
Zheng, Diane: 8010
Zheng, Lidan: 8020
Zheng, Mingyang: 8010
Zheng, Qianyan: 7655
Zheng, Shimin: 8020
Zheng, Xujuan: 7710
Zheng, Yaguang: 1380, 7630, 8000, 8010
Zheng, Yu: 2040, 7080
Zhong, Chuwen: 3245, 7695
Zhong, Lily: 8015
Zhong, Selena: 1110, 8010
Zhong, YunYing: 2300, 7025, 8015, 8020
Zhou, Dan: 8015
Zhou, Jia-jia: 1215
Zhou, Jiayi: 1455
Zhou, Juliet: 4375
Zhou, Junhong: 4215, 4535
Zhou, Kangyi: 8020
Zhou, Leyi: 3365, 7030, 7540
Zhou, Lulin: 7020
Zhou, Nan: 1180, 7420
Zhou, Shally: 8020
Zhou, Shandong: 8010
Zhou, Shuai: 8090
Zhou, Tianqi: 3360
Zhou, Yu: 1560
Zhou, Yuanjin: 1060, 7655
Zhou, Yunshu: 1115
Zhou, Zexi: 4180, 7045, 7735
Zhu, Dantong: 7275
Zhu, Haoran: 7280
Zhu, Hongge: 3270
Zhu, Jiafeng: 1205
Zhu, Jie: 8020
Zhu, Junmin: 2255
Zhu, Ling: 7520, 7910
Zhu, Pengli: 7275, 7710
Zhu, Ruowei: 7320
Zhu, Shida: 7080
Zhu, Shijun: 8015, 8020
Zhu, Weiwei: 1265, 8010
Zhu, Xi: 2215
Zhu, Xiang: 8000
Zhu, Xiaofan: 4110
Zhu, Xiaoqian: 4440
Zhu, Yiwen: 3035
Zhu, Yujun: 7665, 8010
Zhu, Yun: 8000
Zhu, Zheng: 3305, 3505, 4030
Zhuang, Jia: 7615
Zhuang, Jie: 7615
Zhuang, Tianchi: 8000
Zieschang, Tania: 8015
Zimbinski, William J: 7455
Zimmer, Chantelle: 3465
Zimmer, Rachel: 8015

Zimmer, Zachary: 4050, 8020
Zimmerman, Elizabeth: 1490
Zimmerman, Luke: 1070
Zimmerman, M. Bridget: 7205
Zimmerman, Scott C: 1545
Zimmerman, Sheryl: 1520, 2105, 2480, 3000, 8010, 9040
Zimmermann, Jaroslava: 1285
Zisberg, Anna: 3465, 4040, 4045, 4075, 7265, 7400, 7920, 8010, 8020
Zlatar, Zvinka Z: 7720
Zmuda, Joseph M: 7010, 7060, 7625
Zoaktafi, Mojgan: 2360
Zólyomi, Eszter: 2060
Zonderman, Alan B: 1250, 2055, 2330
Zonderman, Alan: 8000
Zonker, Christina: 1125, 7425
Zonsius, Mary C: 1490
Zou, Baiming: 7535
Zou, Ning: 1355, 2355
Zubatsky, Max J: 8020
Zuber, Mohammed: 7200
Zuelsdorff, Megan: 2335
Zuendel, Matthias: 2140
Zullig, Leah L: 7685
Zuniga, Franziska: 4465
Zuo, Dongmei: 7060
Zuraw, Matthew: 7005, 7135
Zurek, Alina: 8010
Zurek, Grzegorz: 8010
Zwakhalen, Sandra MG: 4255
Zweibaum, David: 9015
